# Meat consumption and risk of incident dementia: cohort study of 493,888 UK Biobank participants

**DOI:** 10.1093/ajcn/nqab028

**Published:** 2021-03-22

**Authors:** Huifeng Zhang, Darren C Greenwood, Harvey A Risch, David Bunce, Laura J Hardie, Janet E Cade

**Affiliations:** Nutritional Epidemiology Group, School of Food Science and Nutrition, University of Leeds, Leeds, UK; Leeds Institute for Data Analytics, Faculty of Medicine and Health, University of Leeds, Leeds, UK; Department of Chronic Disease Epidemiology, Yale School of Public Health, New Haven CT, USA; School of Psychology, Faculty of Medicine and Health, University of Leeds, Leeds, UK; Division of Clinical and Population Sciences, Leeds Institute of Cardiovascular and Metabolic Medicine, School of Medicine, University of Leeds, Leeds, UK; Nutritional Epidemiology Group, School of Food Science and Nutrition, University of Leeds, Leeds, UK

**Keywords:** dementia, Alzheimer disease, vascular dementia, meat consumption, processed meat, UK Biobank

## Abstract

**Background:**

Worldwide, the prevalence of dementia is increasing and diet as a modifiable factor could play a role. Meat consumption has been cross-sectionally associated with dementia risk, but specific amounts and types related to risk of incident dementia remain poorly understood.

**Objective:**

We aimed to investigate associations between meat consumption and risk of incident dementia in the UK Biobank cohort.

**Methods:**

Meat consumption was estimated using a short dietary questionnaire at recruitment and repeated 24-h dietary assessments. Incident all-cause dementia comprising Alzheimer disease (AD) and vascular dementia (VD) was identified by electronic linkages to hospital and mortality records. HRs for each meat type in relation to each dementia outcome were estimated in Cox proportional hazard models. Interactions between meat consumption and the apolipoprotein E *(APOE)* ε4 allele were additionally explored.

**Results:**

Among 493,888 participants included, 2896 incident cases of all-cause dementia, 1006 cases of AD, and 490 cases of VD were identified, with mean ± SD follow-up of 8 ± 1.1 y. Each additional 25 g/day intake of processed meat was associated with increased risks of incident all-cause dementia (HR: 1.44; 95% CI: 1.24, 1.67; *P*-trend < 0.001) and AD (HR: 1.52; 95% CI: 1.18, 1.96; *P*-trend = 0.001). In contrast, a 50-g/d increment in unprocessed red meat intake was associated with reduced risks of all-cause dementia (HR: 0.81; 95% CI: 0.69, 0.95; *P*-trend = 0.011) and AD (HR: 0.70; 95% CI: 0.53, 0.92; *P*-trend = 0.009). The linear trend was not significant for unprocessed poultry and total meat. Regarding incident VD, there were no statistically significant linear trends identified, although for processed meat, higher consumption categories were associated with increased risks. The *APOE* ε4 allele increased dementia risk by 3 to 6 times but did not modify the associations with diet significantly.

**Conclusion:**

These findings highlight processed-meat consumption as a potential risk factor for incident dementia, independent of the *APOE* ε4 allele.

See corresponding editorial on page 7 and article on page 154.

## Introduction

Dementia is a major public health concern with around 50 million cases globally and an incidence of nearly 10 million new cases per annum (1, 2). It comprises Alzheimer disease (AD), which contributes to 50–70% of dementia cases, vascular dementia (VD), which contributes to ∼25%, and other forms of dementia (2, 3). Dementia development and progression are associated with both genetic and environmental factors, including diet and lifestyle (4, 5). Lifestyle-related and dietary factors associated with dementia are potentially modifiable and thus represent targets for primary prevention (6).

Meat consumption has gained increasing interest in relation to health, since high consumption of processed meat and probably red meat were found to be consistently associated with an increased risk of colorectal cancer (7). In recent decades meat consumption has doubled or even tripled globally, especially in developing countries (8). This dietary transition has been associated with increasing AD prevalence in Japan, Peru, Cuba and other low- and middle-income countries in both ecological and cross-sectional studies (9, 10). A study of cognitively healthy individuals in Sweden showed that low consumption of meat and meat products was associated with better cognitive performance in clinical dementia screening tests and greater total brain volume after a 5-y follow-up period (11). Our previous review on meat consumption and cognitive disorders including dementia showed that most meat-related studies were embedded in complex dietary patterns with considerable heterogeneity, and the evidence of associations between risk of dementia and specific types or amounts of meat consumption was limited (12).

A consistent association has been established between carriage of the apolipoprotein E (*APOE*) ε4 allele and elevated risk of dementia or AD (13). Previous stratified analyses by *APOE* ε4 status showed that unfavourable lifestyle factors (e.g., less healthy dietary pattern, less physical activity, smoking, and social isolation) were associated with higher risk of dementia in *APOE* ε4 noncarriers but not in carriers (14). The discrepancy between carriers and noncarriers indicates that *APOE* genotype may modify associations between lifestyle factors and dementia risks, and might be explained by a potential masking of weak associations from lifestyle factors by the strongly associated *APOE* ε4 allele. However, at present whether *APOE* ε4 allele carriage interacts with lifestyle factors, such as diet, influencing risk of dementia remains unclear.

In the present study we examined the hypothesis that high consumption of meat increases the incidence of dementia in the general population, which may be more pronounced among *APOE* ε4 noncarriers.

## Methods

### Study design

The UK Biobank is a largescale population-based cohort study of half a million participants aged 40–69 y recruited from across the United Kingdom between 2006 and 2010 (15). The Biobank recruited participants using National Health Service patient registers and conducted the baseline assessments across 22 assessment centers in England, Scotland, and Wales which included a touchscreen questionnaire, verbal interview, physical measures, and biosample collection. At recruitment, participants electronically signed consent forms and completed various touchscreen questionnaires and measurements. All available resources are listed on the UK Biobank website (http://www.ukbiobank.ac.uk/resources/). Ethical approval was granted for the UK Biobank by the North West–Haydock Research Ethics Committee (REC reference: 16/NW/0274). The UK Biobank dataset for this project included 502,493 participants.

### Meat consumption measures

At the recruitment assessment-center visit, each participant was asked to complete a brief touchscreen FFQ with 47 dietary items covering main foods, food groups, and drinking habits (16). The meat-related questionnaire items (fish not included) examined in the current study were the following: processed meat (such as bacon, ham, sausages, meat pies, kebabs, burgers, chicken nuggets), unprocessed poultry, unprocessed beef, unprocessed lamb/mutton, and unprocessed pork. Consumption of unprocessed beef, lamb, and pork were summed to provide the “unprocessed red meat” type, and all meat items listed above were combined into “total meat.” Frequencies of consumption consisted of 6 categories and were assigned values for frequency per week (never eaten = 0, eaten <1 time/wk = 0.5, 1 time/wk = 1, 2–4 times/wk = 3, 5–6 times/wk = 5.5, and ≥1 time daily = 7). We categorized intake frequencies for each meat type into 5 groups as follows: processed meat (0, 0.1–0.9, once, 2.0–4.9, and ≥ 5.0 times/wk), unprocessed poultry (0, 0.1–0.9, once, 2.0–4.9, and ≥ 5.0 times/wk), unprocessed red meat (0, 0.1–1.0, 1.1–1.9, 2.0–2.9, and ≥ 3.0 times/wk), and total meat (0, 0.1–3.0, 3.1–4.9, 5.0–6.9, and ≥ 7.0 times/wk). These categories were determined based on data distribution to provide similar-sized groups (additional details in **Supplemental Methods 1**).

As an enhancement to the baseline touchscreen brief FFQ, the Oxford WebQ dietary questionnaire (17), which assesses a more detailed dietary intake over the previous 24 h was added to the assessment centers from April 2009 to September 2010. After that the WebQ questionnaire was administered online once every 3–4 mo and repeated for a total of 4 rounds over a 16-mo period from February 2011 to June 2012 for 24-h dietary assessments. The Oxford WebQ asked participants to select the number of portions for each item they consumed over the previous 24-h period with instructions specifying 1 standard portion size such as 1 sausage, 1 rasher of bacon, or 1 serving of beef. The daily intakes in grams were calculated by multiplying reported numbers of portions by standard portion sizes (16). Similar foods were then combined together into distinct meat types to match the baseline touchscreen questionnaire. A subgroup of participants (*n*  = 126,844) who completed at least two 24-h dietary assessments were included in this study (18) (see comparisons between participants without or with 1+, 2+, and 3+ completions of the Oxford WebQ in **Supplemental Table 1**); values from multiple assessments were averaged for each participant with 2+ completions. We then calculated the mean intakes from the 24-h dietary assessments within each category of meat types from the touchscreen brief FFQ. The corresponding mean daily intakes in each category were used in combination with frequency from the touchscreen questionnaire as continuous variables to examine the effect sizes per specific increment of meat intakes (25 g/d for processed meat and unprocessed poultry; 50 g/d for unprocessed red meat and total meat). These increments correspond to usual average portion sizes for regular eaters of these products, especially in men in the UK Biobank (19), and are consistent with other study presentations of results (20). The mean daily intakes in each meat category were also used to test the *P*-trend across 5 categories of each meat type, as well as to correct for the potential regression dilution bias in the touchscreen brief FFQ reported in previous studies (16, 21) (more details seen in Supplemental Methods 1).

### Ascertainment of dementia

Prevalent and incident dementia cases within the UK Biobank were ascertained through data linkage to hospital inpatient admissions and death registries. Self-reported dementia cases at recruitment were additionally classified as prevalent cases. The electronic linkage to hospital inpatient data and death registry records includes primary or secondary events across healthcare systems in England, Scotland, and Wales. Date of diagnosis was set as the earliest date of dementia codes recorded regardless of source used. According to the International Classification of Diseases (ICD), AD was defined as code 331.0 in edition 9 and codes F00 and G30 in edition 10; VD was defined as codes 290.4 in edition 9 and codes F01 and I67.3 in edition 10; all-cause dementia was defined as all of the above codes plus ICD-9 codes 290, 291.2, 294.1, 331.0–331.2, and 331.5, and ICD-10 codes A81.0, F02, F05.1, F10.6, G31.0, G31.1, and G31.8. The updating date of linkages to hospital inpatient admission and death registries was 31 March 2017 in England, 31 October 2016 in Scotland, and 29 February 2016 in Wales in this study. Participant survival time in person-y was calculated from the date of dietary assessment until date of dementia diagnosis, date of loss to follow-up, date of death, or updating date of linkages.

### *APOE* genotyping

Genotypes of nearly one-half million participants in the UK Biobank were assayed using 2 very similar genotyping arrays manufactured by Affymetrix: the BiLEVE Axiom array for ∼50,000 participants and the UK Biobank Axiom array for the remaining ∼450,000 participants; genotyping quality control was performed by UK Biobank centrally (22). Data from UK Biobank participants with unusually high heterozygosity and missingness (>5%) and disagreement between reported sex and genetic sex were excluded in genotype-related analyses (23). In addition, we used genetic kinship to other participants (Biobank field ID 22,021) as a covariate to limit confounding from population relatedness (24). The APOE haplotypes (ε2/ε3/ε4) were directly genotyped and determined by 2 genetic variants, rs429358 and rs7412. Participants with 1 or 2 ε4 alleles were defined as *APOE* ε4 carriers and otherwise as *APOE* ε4 noncarriers. After quality control procedures, *APOE* genotypes were available on 405,126 UK Biobank participants and were included in *APOE* genotype related analyses.

### Statistical analysis

Participants with prevalent dementia, and those with incomplete data on meat-related variables were excluded before analyses. Given the possibility that underlying dementia may cause changes in dietary behaviors in advance of diagnosis, we excluded incident dementia cases that occurred in the first-y period from baseline dietary data collection to dementia diagnosis to limit the possibility of reverse causality (25). A more stringent 3-y cut-off was also applied as a sensitivity analysis (see the flowchart in **Supplemental Figure 1**).

Baseline sociodemographic, lifestyle, and main dietary characteristics were summarized and stratified by dementia status (incident dementia and no dementia). Among incident cases, all-cause dementia, AD, and VD were treated as separate outcomes. The associations between incident dementia and reported consumption of processed meat, unprocessed poultry, unprocessed red meat, and total meat were fitted in Cox proportional hazards regressions with the duration of follow-up in years as the timescale and the second lowest category of meat intakes as the reference; HRs with 95% CIs were reported for all analyses.

Three models were applied in our analyses: unadjusted models, minimally adjusted models, and fully adjusted models. The minimally adjusted model was adjusted for age at baseline, gender, self-reported ethnicity (White, Asian, Black, mixed, other/unknown), socioeconomic status (low, moderate, or high deprivation), educational level (with university/college degree or not), determined by a directed acyclic graph (26) (**Supplemental Methods 2**). The fully adjusted model was additionally adjusted for region (England, Wales, Scotland), BMI (in kg/m^2^; <25, 25–29.9, and ≥30), physical activity level (low, moderate, and high), smoking status (never, past, and current), typical sleep duration (<7, 7–8, >8 h/d), stroke history, family history of dementia, and dietary factors including total consumption of vegetables and fruits, total fish, tea and coffee, and alcohol. Processed meat, unprocessed poultry, and unprocessed red meat were also mutually adjusted for in the models. More details on covariates can be seen in **Supplemental Methods 3**. For covariates where participants answered “do not know” or “prefer not to answer,” these responses were classified as missing. An “unknown” category was created to replace missing values for each covariate; the effect of replacement of missing values was assessed by a sensitivity analysis conducted in participants with complete data on all covariates.

To investigate potential modifying effects of the *APOE* ε4 allele on risk of dementia from meat consumption, stratified analyses by *APOE* ε4 carrying status were conducted and additionally *P*-interaction between each meat type and *APOE* ε4 status was tested. As a sensitivity analysis, the main analyses were repeated among participants aged ≥60 y at baseline since individuals aged >60 y have a higher risk of incident dementia (27). Statistical analyses were conducted using Stata/IC, version 16.1 (Stata Corp LP).

## Results

During a mean follow-up of 8 ± 1.1 y, excluding cases arising in the first year of follow-up (*n* = 77), 2896 incident cases of all-cause dementia occurred, of which 1006 were AD and 490 were VD. Baseline characteristics stratified by dementia status are provided in **Table 1**. Dementia cases were generally older, more economically deprived, less educated, more likely to smoke, less physically active, more likely to have stroke history and family dementia history, and more likely to be *APOE* ε4 carriers. More men than women were diagnosed with dementia in the study population. Participant characteristics across 5 categories of reported consumption of processed meat, unprocessed poultry, unprocessed red meat, and total meat are shown in **Supplemental Tables 2, 3, 4**, and **5** respectively. Generally, compared with those in the lowest category, participants in higher categories of reported consumption of processed meat and total meat were more likely to be men, less educated, smokers, and overweight or obese, and had lower intakes of vegetables and fruits and higher intakes of energy, protein, and fat (including saturated fat).

**TABLE 1:** Baseline characteristics of participants stratified by dementia status in the UK Biobank cohort study^1^

	All participants (*n* = 493,888)	Incident dementia (*n* = 2896)	No dementia (*n* = 490,992)
Age at baseline, y	56.5 ± 8.1	63.7 ± 5.5	56.5 ± 8.1
Duration of follow-up, y	8.0 ± 1.1	5.9 ± 2.1	8.0 ± 1.1
Gender			
Men	224,691 (45.5%)	1625 (56.1%)	223,066 (45.4%)
Women	269,197 (54.5%)	1271 (43.9%)	267,926 (54.6%)
Ethnicity			
White	466,835 (94.5%)	2757 (95.2%)	464,078 (94.5%)
Asian	10,737 (2.2%)	44 (1.5%)	10,693 (2.2%)
Black	7454 (1.5%)	52 (1.8%)	7402 (1.5%)
Mixed	2951 (0.6%)	13 (0.4%)	2938 (0.6%)
Others/unknown	5911 (1.2%)	30 (1.0%)	5881 (1.2%)
Region			
England	438,178 (88.7%)	2510 (86.7%)	435,668 (88.7%)
Wales	20,505 (4.2%)	121 (4.2%)	20,384 (4.2%)
Scotland	35,205 (7.1%)	265 (9.2%)	34,940 (7.1%)
Townsend deprivation index			
Low deprivation	164,443 (33.3%)	858 (29.6%)	163,585 (33.3%)
Moderate deprivation	164,409 (33.3%)	876 (30.2%)	163,533 (33.3%)
High deprivation	164,426 (33.3%)	1160 (40.1%)	163,266 (33.3%)
Unknown	610 (0.1%)	2 (0.1%)	608 (0.1%)
Educational level			
Without college/university degree	327,638 (66.3%)	2245 (77.5%)	325,393 (66.3%)
With college/university degree	161,496 (32.7%)	582 (20.1%)	160,914 (32.8%)
Unknown	4754 (1.0%)	69 (2.4%)	4685 (1.0%)
Smoking status			
Never	269,599 (54.6%)	1273 (44.0%)	268,326 (54.6%)
Past	170,941 (34.6%)	1233 (42.6%)	169,708 (34.6%)
Current	51,734 (10.5%)	371 (12.8%)	51,363 (10.5%)
Unknown	1614 (0.3%)	19 (0.7%)	1595 (0.3%)
Physical activity level			
Low	75,335 (15.3%)	478 (16.5%)	74,857 (15.2%)
Moderate	162,588 (32.9%)	882 (30.5%)	161,706 (32.9%)
High	160,784 (32.6%)	779 (26.9%)	160,005 (32.6%)
Unknown	95,181 (19.3%)	757 (26.1%)	94,424 (19.2%)
BMI, kg/m^2^			
Normal/underweight (<25)	162,906 (33.0%)	893 (30.8%)	162,013 (33.0%)
Overweight (25–29.9)	208,812 (42.3%)	1184 (40.9%)	207,628 (42.3%)
Obese (≥30)	119,702 (24.2%)	775 (26.8%)	118,927 (24.2%)
Unknown	2468 (0.5%)	44 (1.5%)	2424 (0.5%)
Sleep duration			
<7 h/d	120,987 (24.5%)	750 (25.9%)	120,237 (24.5%)
7–8 h/d	332,852 (67.4%)	1687 (58.3%)	331,165 (67.4%)
>8 h/d	37,564 (7.6%)	415 (14.3%)	37,149 (7.6%)
Unknown	2485 (0.5%)	44 (1.5%)	2441 (0.5%)
With stroke history	7397 (1.5%)	177 (6.1%)	7220 (1.5%)
With family history of dementia	57,728 (11.7%)	558 (19.3%)	57,170 (11.6%)
*APOE* ε4 carrying status			
Noncarriers	290,382 (58.8%)	1177 (40.6%)	289,205 (58.9%)
Carriers	115,873 (23.5%)	1182 (40.8%)	114,691 (23.4%)
Missing	87,633 (17.7%)	537 (18.5%)	87,096 (17.7%)
Total meat			
Never	20,473 (4.1%)	94 (3.2%)	20,379 (4.2%)
≤3 times/wk	77,261 (15.6%)	459 (15.8%)	76,802 (15.6%)
3–5 times/wk	90,065 (18.2%)	509 (17.6%)	89,556 (18.2%)
≥5 times/wk	162,570 (32.9%)	875 (30.2%)	161,695 (32.9%)
≥7 times/wk	143,519 (29.1%)	959 (33.1%)	142,560 (29.0%)
Vegetables/fruits			
<2 servings/d	28,960 (5.9%)	194 (6.7%)	28,766 (5.9%)
<4 servings/d	133,350 (27.0%)	638 (22.0%)	132,712 (27.0%)
4–6 servings/d	190,853 (38.6%)	1032 (35.6%)	189,821 (38.7%)
>6 servings/d	128,487 (26.0%)	893 (30.8%)	127,594 (26.0%)
Unknown	12,238 (2.5%)	139 (4.8%)	12,099 (2.5%)
Total fish			
≤1 times/wk	126,980 (25.7%)	678 (23.4%)	126,302 (25.7%)
1–2 times/wk	107,219 (21.7%)	520 (18.0%)	106,699 (21.7%)
≥2 times/wk	150,200 (30.4%)	865 (29.9%)	149,335 (30.4%)
≥4 times/wk	106,331 (21.5%)	791 (27.3%)	105,540 (21.5%)
Unknown	3158 (0.6%)	42 (1.5%)	3116 (0.6%)
Alcohol			
<1 time/wk	150,575 (30.5%)	1075 (37.1%)	149,500 (30.4%)
1–2 times/wk	127,529 (25.8%)	664 (22.9%)	126,865 (25.8%)
3–4 times/wk	114,501 (23.2%)	536 (18.5%)	113,965 (23.2%)
Daily or almost daily	100,944 (20.4%)	610 (21.1%)	100,334 (20.4%)
Unknown	339 (0.1%)	11 (0.4%)	328 (0.1%)
Tea/coffee			
≤3 cups/d	108,836 (22.0%)	663 (22.9%)	108,173 (22.0%)
≤5 cups/d	161,965 (32.8%)	918 (31.7%)	161,047 (32.8%)
≤7 cups/d	132,660 (26.9%)	698 (24.1%)	131,962 (26.9%)
>7 cups/d	88,987 (18.0%)	593 (20.5%)	88,394 (18.0%)
Unknown	1440 (0.3%)	24 (0.8%)	1416 (0.3%)

^1^Continues variables displayed as means ± SDs, and categorical variables are displayed as numbers (percentages). *APOE*, apolipoprotein E.

**FIGURE 1:**
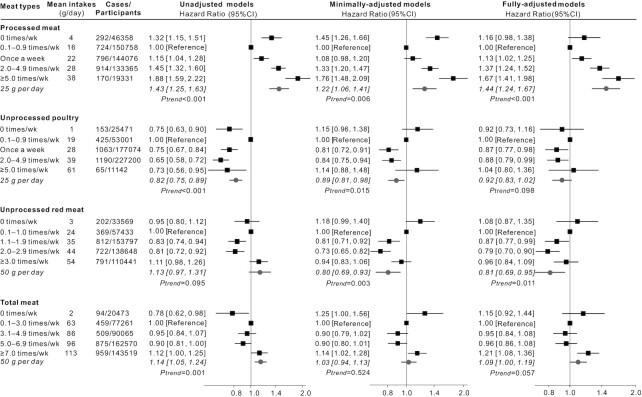
HRs (95% CIs) for the associations between incident all-cause dementia and meat consumption in UK Biobank (*n* = 493,888). The black squares and horizontal lines represent HRs and 95% CIs respectively in Cox proportional-hazards regressions. The distribution of ticks on the *x* axis is exponential. Participants were categorized based on the data distribution of baseline meat intakes. Mean daily intakes in each category were calculated from the multiple 24-h dietary assessments which were used to test the linear trend per increment. Minimally adjusted models adjusted for age, gender, ethnicity, education, socioeconomic status. Fully adjusted models additionally adjusted for region, smoking status, physical activity, BMI, sleep duration, stroke history, and family history of dementia, and dietary covariates including vegetables and fruits, total fish, tea and coffee, alcohol drinking, processed meat, unprocessed poultry, and unprocessed red meat were also mutually adjusted for.

The associations between each meat type and each dementia outcome were analyzed in three adjustment models. For the incident all-cause dementia (**Figure 1**), there was a significant linear trend for each additional 25 g processed meat consumed per day (HR: 1.44; 95% CI: 1.24, 1.67; *P-*trend < 0.001). Unprocessed red meat appeared to be protective, with a HR of 0.81 for each additional 50 g intake per day (95% CI: 0.69, 0.95; *P*-trend = 0.011) in the fully adjusted model. The linear-trend was not statistically significant for unprocessed poultry in relation to risk of all-cause dementia. For total meat, there was a borderline increased risk of incident all-cause dementia (HR: 1.09; 95% CI: 1.00, 1.19; *P*-trend = 0.057).

**FIGURE 2:**
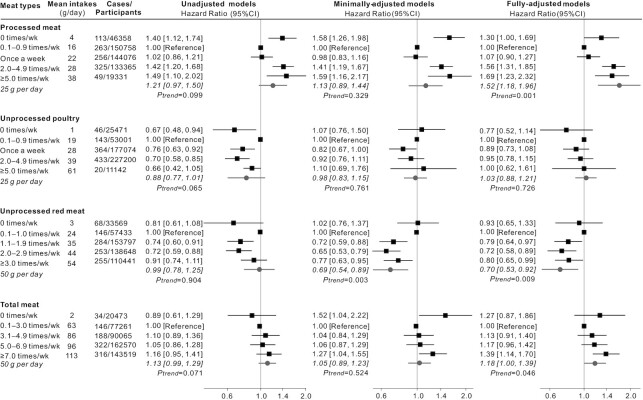
HRs (95% CIs) for the associations between incident Alzheimer disease and meat consumption in UK Biobank (*n* = 493,888). The black squares and horizontal lines represent HRs and 95% CI, respectively, in Cox proportional-hazards regressions. The distribution of ticks on the *x* axis is exponential. Participants were categorized based on the data distribution of baseline meat intakes. Mean daily intakes in each category is calculated from the multiple 24-h dietary assessments which were used to test the linear trend per increment. Minimally adjusted models adjusted for age, gender, ethnicity, education, and socioeconomic status. Fully adjusted models additionally adjusted for region, smoking status, physical activity, BMI, sleep duration, stroke history, family history of dementia, and dietary covariates including vegetables and fruits, total fish, tea and coffee, alcohol drinking; processed meat, unprocessed poultry, and unprocessed red meat were also mutually adjusted for.

**FIGURE 3:**
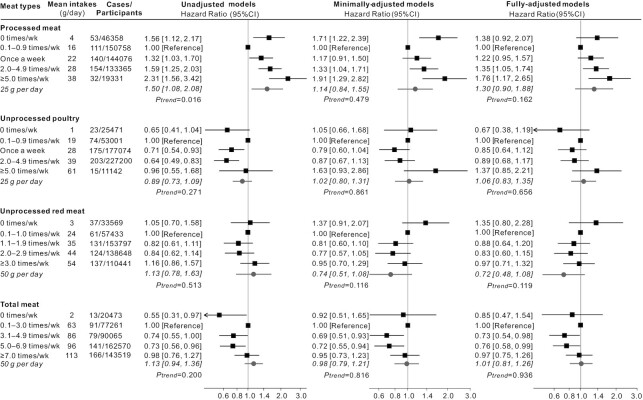
HRs (95% CIs) for the associations between incident vascular dementia and meat consumption in UK Biobank (*n* = 493,888). The black squares and horizontal lines represent HRs and 95% CI respectively in Cox proportional-hazards regressions. The distribution of ticks on the *x* axis is exponential. Participants were categorized based on the data distribution of baseline meat intakes. Mean daily intakes in each category is calculated from the multiple 24-h dietary assessments which were used to test the linear trend per increment. Minimally adjusted models adjusted for age, gender, ethnicity, education, socioeconomic status. Fully adjusted models additionally adjusted for region, smoking status, physical activity, BMI, sleep duration, stroke history, and family history of dementia, and dietary covariates including vegetables and fruits, total fish, tea and coffee, alcohol drinking; processed meat, unprocessed poultry, and unprocessed red meat were also mutually adjusted for.

In terms of incident AD (**Figure 2**), a similar picture to all-cause dementia was seen. Higher consumption of processed meat was associated with increased risk of AD (HR: 1.52 per additional 25 g/d; 95% CI: 1.18, 1.96; *P*-trend = 0.001). Higher consumption of unprocessed red meat was associated with reduced risk of AD (HR: 0.70 per additional 50 g/d; 95% CI: 0.53, 0.92; *P*-trend = 0.009). Regarding the risk of incident VD (**Figure 3**), there were no statistically significant linear trends identified, although for processed meat, the highest consumption categories were associated with increased risk. For all dementia outcomes, 0 times/wk consumption of each meat type appeared to be different from other higher frequencies (Figure 1, 2, and 3); however, most HRs in this category were not significant in the fully adjusted models.

The stratified analyses by *APOE* ε4 carrying status and *P* values for interaction between *APOE* ε4 carriage and meat consumption are shown in **Table 2** and **Supplemental Table 6**. Compared with *APOE* ε4 noncarriers, carriers had increased risks of developing all-cause dementia by ∼3 times, AD by ∼6 times, and VD by ∼5 times, independent of any type of meat consumption. However, there were no statistically significant interactions between *APOE* ε4 carriage and meat consumption in the fully adjusted models. Increased risks of incident all-cause dementia were observed per 25 g/d increments of processed meat in both *APOE* ε4 carriers and noncarriers. However, *APOE* ε4 carriers but not noncarriers had reduced risks of incident all-cause dementia and incident AD per 50 g/d increment of unprocessed red meat.

**TABLE 2:** Risks of all-cause dementia under different meat types among APOE Ɛ4 noncarriers (*n*  = 289,589) and carriers ( *n*  = 115,537) respectively ^1^

	Unadjusted models (*n* = 405,126)				Minimally adjusted models (*n* = 405,126)				Fully adjusted models (*n* = 405,126)			
	HR	LCI	UCI	*P*	HR	LCI	UCI	*P*	HR	LCI	UCI	*P*
Risk of all-cause dementia												
*APOE Ɛ4* carriers vs. noncarriers	3.31	2.38	4.61	<0.001	3.59	2.48	5.19	<0.001	3.51	2.44	5.04	<0.001
Processed meat, 25 g/d												
Stratified analysis												
* APOE* Ɛ4 noncarriers	1.64	1.33	2.02	<0.001	1.36	1.09	1.70	0.007	1.46	1.15	1.84	0.002
* APOE* Ɛ4 carriers	1.18	0.96	1.45	0.112	1.09	0.88	1.36	0.436	1.47	1.16	1.85	0.001
*P* for interaction with *APOE Ɛ4 allele*				0.027				0.026				0.185
Unprocessed poultry, 25 g/d												
Stratified analysis												
* APOE* Ɛ4 noncarriers	0.84	0.74	0.96	0.009	0.92	0.79	1.07	0.261	0.93	0.79	1.09	0.379
*APOE* Ɛ4 carriers	0.82	0.73	0.93	0.002	0.89	0.77	1.03	0.111	0.94	0.81	1.09	0.435
*P*-interaction with *APOE Ɛ4* allele				0.787				0.329				0.765
Unprocessed red meat, 50 g/d												
Stratified analysis												
* APOE* Ɛ4 noncarriers	1.31	1.04	1.66	0.023	0.94	0.75	1.19	0.633	0.93	0.72	1.21	0.594
*APOE* Ɛ4 carriers	0.89	0.72	1.11	0.311	0.64	0.51	0.80	<0.001	0.64	0.50	0.82	<0.001
*P*-interaction with *APOE Ɛ4* allele				0.020				0.019				0.095
Total meat, 50 g/d												
Stratified analysis												
APOE Ɛ4 noncarriers	1.22	1.07	1.39	0.003	1.11	0.96	1.28	0.168	1.16	1.00	1.34	0.044
APOE Ɛ4 carriers	1.05	0.93	1.17	0.462	0.95	0.83	1.09	0.469	1.02	0.89	1.17	0.816
*P*-interaction with APOE Ɛ4 allele				0.091				0.062				0.054

1 Minimally adjusted models: Cox proportional-hazards regression adjusted for age, gender, ethnicity, education, socioeconomic status. Fully adjusted models: Cox proportional-hazards regression additionally adjusted for region, smoking status, physical activity, BMI, sleep duration, stroke history, family history of dementia, genetic kinship to other participants, dietary covariates including vegetables and fruits, total fish, tea and coffee, alcohol drinking, processed meat, unprocessed poultry, and unprocessed red meat were also mutually adjusted for. Mean daily intakes per increment calculated from the multiple 24-h dietary assessments were used as continuous variables in Cox models. *APOE*, apolipoprotein E; LCI, lower CI (95%); UCI, upper CI (95%).

When we additionally excluded dementia cases occurring within the first 3-y follow-up (*n* = 329) for more rigorous controls of potential reverse causality, the HRs were of similar magnitude (**Supplemental Figure 2, 3**, and **4, Supplemental Table 7**). When we conducted a sensitivity analysis in participants with complete data on all covariates (*n* = 381,809), the HRs were very similar to the main results (**Supplemental Figure 5, 6**, and **7, Supplemental Table 8**). Exclusion of participants aged <60 y at baseline also did not significantly change these associations (**Supplemental Figure 8, 9**, and **10, Supplemental Table 9**).

## Discussion

In this population-based, nationwide UK Biobank cohort study our results showed that consumption of processed meat was associated with increased risks of incident all-cause dementia and AD while unprocessed red meat was associated with lower risks. Related cohort studies remain few and inconsistent, and detailed knowledge of which type and amount of meat consumption would be the most influential is not clear. The Three-City (3C) cohort study took meat consumption of high frequency (≥4 times/wk) as the reference and found that low frequency (≤1 times/wk) was related to an increased risk of incident dementia and AD over 10 y of follow-up (28), which is inconsistent with our findings; however, the methods of collapsing data and reference selection are different. In addition, excessive category combination may have attenuated the study power and specific meat types were not explored in that study. A cohort study conducted in French citizens aged 68 and over showed that compared with daily meat consumers, weekly or less consumers had a higher incidence rate of all-cause dementia and AD after 7 y of follow-up; however, those associations were not significant probably because of small sample sizes (170 incident dementia including 135 AD among 1674 participants) (29). Longitudinal analysis among 2622 elderly German participants suggested no significant association between risk of incident AD and consumption frequency of meat and sausage after 4 y of follow-up (30); however, this study only investigated single meat items.

Our results also showed that presence of the *APOE* ε4 allele increased the risk of incident dementia, especially AD; however, there were only minor differences in associations between meat consumption and dementia risk among *APOE* ε4 noncarriers and carriers, and all *P* values for interaction were nonsignificant. Currently, evidence on the interaction between *APOE* genotype and dietary factors with dementia has mostly focused on dietary patterns and dietary fat intake; those studies found older individuals (aged ≥60 y) who had a diet high in fatty fish or higher polyunsaturated fat intake were associated with a decreased risk of all-cause dementia, especially among *APOE* ε4 noncarriers (31, 32). In contrast, studies conducted at midlife found that moderate to high intake of saturated fats in relation to an increased risk of dementia/AD was only detected or more pronounced among *APOE* ε4 carriers (33, 34). A German cohort study of individuals aged 75 + found there was no difference in the association of meat and sausage consumption with incident AD risk between *APOE* ε4 noncarriers and carriers (30). In addition, a cohort study from eastern Finland showed that the APOE ε4 genotype did not modify associations of egg and cholesterol intakes with risk of incident dementia and AD over ∼22 y of follow-up (35). Inconsistency in these and our study results may reflect particular cohort characteristics; in particular our participants were younger (50–68 y) and this may have led to our insignificant interactions between *APOE* genotype and meat intake with dementia risk in this population. It is also possible that *APOE* ε4 carriage is an independent process from dietary aspects in relation to dementia risk.

The underlying reasons for the inconsistent associations between different meat types in relation to dementia risk are not understood. High levels of protein in meat may potentially explain the link between unprocessed meat intake and a lower risk of dementia; adequate protein intake has been linked to a reduced risk of mild cognitive impairment and dementia in the elderly (36). High iron levels in unprocessed red meat may be protective, with iron deficiency being associated with decreased cognitive and attentional processes. Studies in animals have shown a negative impact of iron deficiency on myelination (37). On the other hand, as people age, iron deposits in the brain may impair normal cognitive function. Abnormal iron metabolism triggers oxidative stress, a major contributor to neurodegeneration (38). Processed meat contains nitrites and *N*-nitroso compounds, which may result in oxidative stress, lipid peroxidation, and activation of proinflammatory cytokines or other mechanisms potentially involved in the development of dementia (39). In addition, as meat consumption increases, intake of saturated fatty acids increases, which has been associated with a higher risk of dementia (40). Processed meat is often high in sodium, and rats fed a long-term high-salt diet had a marked increase in systolic blood pressure linked to reduced regional cerebral blood flow, and potentially linked to cognitive deficit (41). These differences in nutritional composition may explain why consumption of processed meat was associated with a higher risk of dementia rather than unprocessed poultry and unprocessed red meat. These potentially beneficial and negative effects of different meat types on risk of dementia may exist simultaneously, leading to the inconsistent associations seen with meat in this study.

A major strength of the current study is that the prospective study with large sample sizes ensured sufficient statistical power. To our knowledge, this is the first study to estimate specific meat types in relation to several dementia outcomes with additional exploration of interactions with the *APOE* ε4 allele. Other strengths include use of multiple data linkages to maximize capture of incident dementia outcomes, and consideration of reverse causation in analyses. Nevertheless, our study has several limitations. Firstly, the baseline touchscreen brief FFQ only covered some commonly consumed foods and was not suitable to assess total energy or nutrient intakes; systematic bias from self-reported measures at recruitment and low responses to the more detailed repeated 24-h dietary assessments with less than half participants may limit generalizability. Secondly, the UK Biobank cohort study does not have a long follow-up (∼8 y). This will limit our ability to distinguish between reverse causation and causality for risk factors for dementia, as indicated in the Whitehall II cohort study (42). Thirdly, use of linkages to electronic health records may be high in specificity but low in sensitivity; moreover, without linkage to primary care data in our study, milder cases of dementia may have been missed (43). The percentage of AD out of all-cause dementia cases was low in our study (35%) compared with the report of the WHO (50–70%) (2); it is possible that some cases had not been clinically classified by type of dementia, which may attenuate associations between meat consumption and risk of AD. In addition, taking dates of hospital admission and death registry as proxy of diagnosis dates of incident dementia could have resulted in measurement errors; some incident cases might actually be prevalent cases diagnosed prior to hospital admission. Therefore, electronic linkages to accurate primary-care data should be taken into consideration for dementia ascertainment in future research.

Our findings suggest that consumption of processed meat may increase risk of incident dementia, and unprocessed red meat intake may be associated with lower risks, independent of *APOE* ε4 carriage. On the basis of the findings of this study, more specific public health guidance could be indicated differentiating between types of meat. However further research is recommended to confirm these results. Overall, the research adds to the growing body of evidence linking meat, especially processed meat consumption, to increased risk of a range of noncommunicable diseases.

## Supplementary Material

nqab028_Supplemental_File

## Data Availability

The data sets described in the manuscript are not publicly available because the UK Biobank has proprietary rights of the data. External investigators can request the data and approval of use on application to the UK Biobank (www.ukbiobank.ac.uk/).

## Notes

This work was supported by the China Scholarship Council and the University of Leeds to HZ (201806010423). The sponsors had no role in the design and conduct of the study; collection, management, analysis, and interpretation of the data; and preparation, review, or approval of this manuscript.

Supplemental Methods 1–3, Supplemental Tables 1–9, and Supplemental Figures 1–10 are available from the “Supplementary data” link in the online posting of the article and from the same link in the online table of contents at https://academic.oup.com/ajcn/.

Abbreviations used: AD, Alzheimer disease; *APOE*, apolipoprotein E; ICD, International Classification of Diseases; VD, vascular dementia.
