# Supplementary material for: Meat consumption and risk of incident dementia: cohort study of 493,888 UK Biobank participants
Source: Am J Clin Nutr. 2021 Mar 22;114(1):175–84. doi: 10.1093/ajcn/nqab028 (PMC8246598; doi:10.1093/ajcn/nqab028)
Supplement: nqab028_Supplemental_File [file nqab028_supplemental_file.docx]

**Meat consumption and risk of incident dementia: cohort study of 493888 UK Biobank participants**

Huifeng Zhang ^1*^, Darren C Greenwood ^2^, Harvey A Risch ^3^, David Bunce ^4^, Laura J Hardie ^5^, Janet E Cade ^1^

^1^ Nutritional Epidemiology Group, School of Food Science and Nutrition, University of Leeds, Leeds LS2 9JT, UK.

^2^ Division of Biostatistics, Faculty of Medicine and Health, University of Leeds, Leeds LS2 9JT, UK

^3^ Department of Chronic Disease Epidemiology, Yale School of Public Health, New Haven CT USA

^4^ School of Psychology, Faculty of Medicine and Health, University of Leeds, Leeds LS2 9JT, UK

^5^ Division of Clinical and Population Sciences, Leeds Institute of Cardiovascular and Metabolic Medicine, School of Medicine, University of Leeds, Leeds LS2 9JT, UK

*Correspondence: Huifeng Zhang, School of Food Science and Nutrition, University of Leeds, Leeds LS2 9JT, UK.

Email: [fshz@leeds.ac.uk](mailto:fshz@leeds.ac.uk), Tel: +44(0)113 343 7769, ORCID: 0000-0002-9818-7904

**Supplementary materials**

[Supplementary Figures 4](#_Toc60845554)

[**Supplementary Figure 1** **Flowchart of participants in the UK Biobank cohort study** 4](#_Toc60845555)

[**Supplementary Figure 2** **Hazard ratios (95% CIs) for the associations between incident all-cause dementia and meat consumption with excluding cases arising in first 3 years of follow-up (n=493559)** 5](#_Toc60845556)

[**Supplementary Figure 3 Hazard ratios (95% CIs) for the associations between incident Alzheimer’s disease and meat consumption with excluding cases arising in first 3 years of follow-up (n=493559)** 6](#_Toc60845557)

[**Supplementary Figure 4 Hazard ratios (95% CIs) for the associations between incident vascular dementia and meat consumption with excluding cases arising in first 3 years of follow-up (n=493559)** 7](#_Toc60845558)

[**Supplementary Figure 5 Hazard ratios (95% CIs) for the associations between incident all-cause dementia and meat consumption in participants with complete data on covariates (n=381809)** 8](#_Toc60845559)

[**Supplementary Figure 6 Hazard ratios (95% CIs) for the associations between incident Alzheimer’s disease and meat consumption in participants with complete data on covariates (n=381809)** 9](#_Toc60845560)

[**Supplementary Figure 7 Hazard ratios (95% CIs) for the associations between incident vascular dementia and meat consumption in participants with complete data on covariates (n=381809)** 10](#_Toc60845561)

[**Supplementary Figure 8 Hazard ratios (95% CIs) for the associations between incident all-cause dementia and meat consumption in participants aged 60 or more (n=213668)** 11](#_Toc60845562)

[**Supplementary Figure 9 Hazard ratios (95% CIs) for the associations between incident Alzheimer’s disease and meat consumption in participants aged 60 or more (n=213668)** 12](#_Toc60845563)

[**Supplementary Figure 10 Hazard ratios (95% CIs) for the associations between incident vascular dementia and meat consumption in participants aged 60 or more (n=213668)** 13](#_Toc60845564)

[Supplementary Tables 14](#_Toc60845565)

[**Supplementary Table 1 Baseline characteristics of participants with various numbers of completions of 24-h dietary assessment in UK Biobank cohort study** 14](#_Toc60845566)

[**Supplementary Table 2 Baseline characteristics of participants across categories of processed meat intakes in UK Biobank cohort study** 18](#_Toc60845567)

[**Supplementary Table 3 Baseline characteristics of participants across categories of unprocessed poultry intakes in UK Biobank cohort study** 21](#_Toc60845568)

[**Supplementary Table 4 Baseline characteristics of participants across categories of unprocessed red meat intakes in UK Biobank cohort study** 24](#_Toc60845569)

[**Supplementary Table 5 Baseline characteristics of participants across categories of total meat intakes in UK Biobank cohort study** 27](#_Toc60845570)

[**Supplementary Table 6 Risks of Alzheimer’s disease and vascular dementia under different meat types among APOE Ɛ4 non-carriers (n=289 589) and carriers (n=115 537) respectively** 30](#_Toc60845571)

[**Supplementary Table 7 Risks of all-cause dementia, Alzheimer’s disease and vascular dementia under different meat types among APOE Ɛ4 non-carriers (n=289 441) and carriers (n=115 421) respectively in sensitivity analysis excluding dementia cases within first 3 years of follow-up** 32](#_Toc60845572)

[**Supplementary Table 8 Risks of all-cause dementia, Alzheimer’s disease and vascular dementia under different meat types among APOE Ɛ4 non-carriers (n=225 130) and carriers (n=90 072) respectively in sensitivity analysis excluding participants with missing data of covariates** 35](#_Toc60845573)

[**Supplementary Table 9 Risks of all-cause dementia, Alzheimer’s disease and vascular dementia under different meat types among APOE Ɛ4 non-carriers (n=125 229) and carriers (n=49 440) respectively in participants aged 60 or more** 38](#_Toc60845574)

[Supplementary Methods 41](#_Toc60845575)

[**1. Assessment of dietary meat consumption 41**](#_Toc60845576)

[**1.1 The baseline touchscreen questionnaire 41**](#_Toc60845577)

[**1.2 The Oxford WebQ questionnaire for 24-h dietary assessments 41**](#_Toc60845578)

[**2. Determination of the minimal adjustment set 42**](#_Toc60845579)

[**3. Covariates 43**](#_Toc60845580)

[**3.1 Dietary variables 43**](#_Toc60845581)

[**3.2 Socio-demographics 44**](#_Toc60845582)

[**3.3 Lifestyle related and other covariates 45**](#_Toc60845583)

[Supplementary References 46](#_Toc60845584)

# Supplementary Figures

Total participants at baseline recruitment (2006-2010) in our study released from the UK Biobank (n=502493)
(n = 7)

Sensitivity analysis in participants aged 60 or more (n = 213668)

Sensitivity analysis in participants with complete data on covariates (n = 381809)

Sensitivity analysis excluding cases arising in first 3 years of follow-up
(n = 493559)

Subgroup of *APOE* genotyping eligible
(n = 405126) including Ɛ4 allele non-carriers (n=289 589) and carriers (n=115 537)

Participants in analyses
(n = 493888)

Participants excluded:

- Prevalent dementia cases (n = 564)
- Individuals with missing data on meat intake (n = 7964)
- Incident cases over the first year of follow-up (n = 77)

Subgroup of *APOE* genotyping eligible
(n = 404862) including Ɛ4 allele non-carriers (n=289441) and carriers (n=115421)

Subgroup of *APOE* genotyping eligible
(n = 315202) including Ɛ4 allele non-carriers (n=225130) and carriers (n=90072)

Subgroup of *APOE* genotyping eligible
(n = 174669) including Ɛ4 allele non-carriers (n=125229) and carriers (n=49440)

**Supplementary Figure 1** **Flowchart of participants in the UK Biobank cohort study**


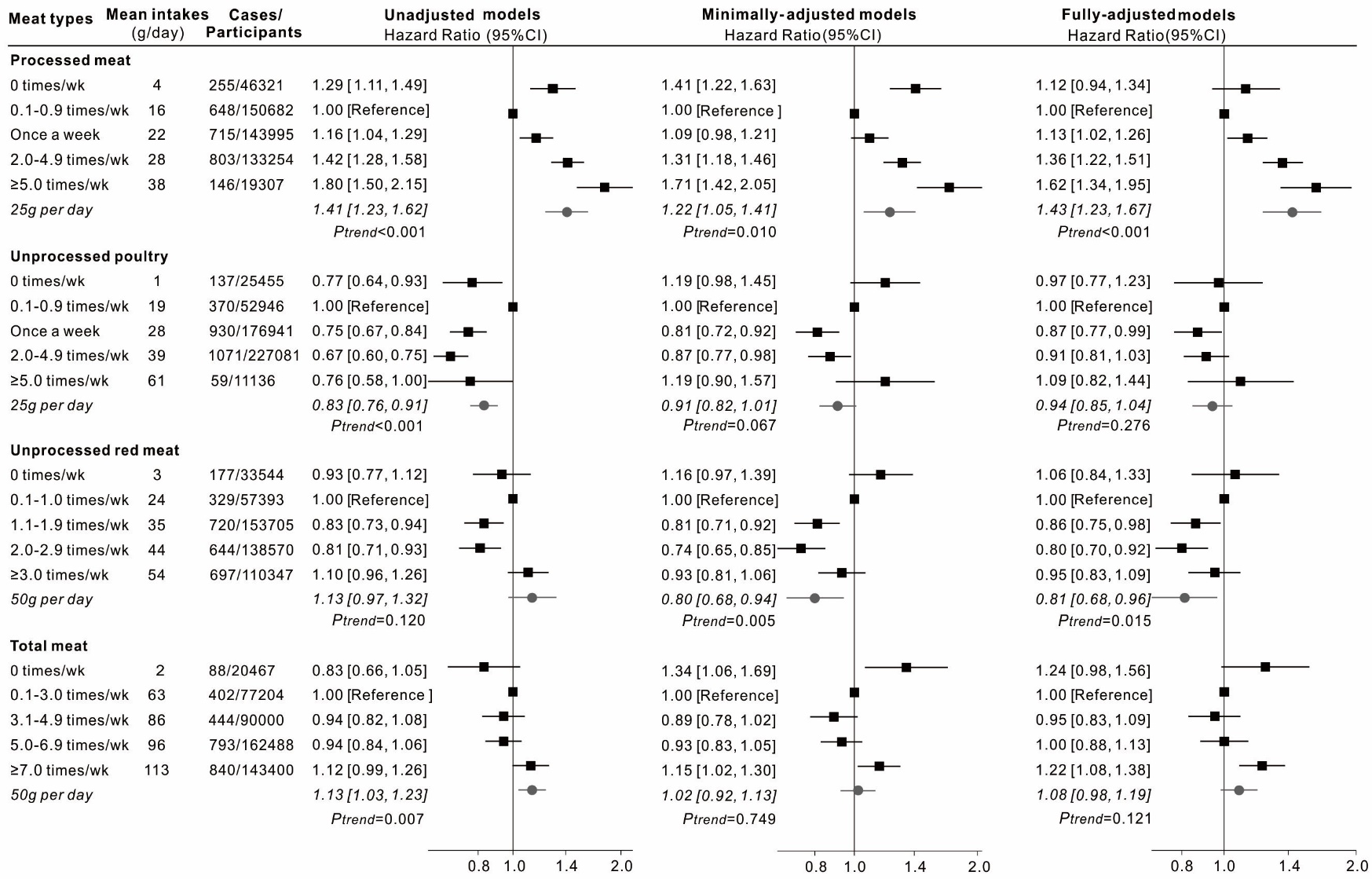


**Supplementary Figure 2** **Hazard ratios (95% CIs) for the associations between incident all-cause dementia and meat consumption with excluding cases arising in first 3 years of follow-up (n=493559)** The black squares and horizontal lines represent hazard ratios and 95% confidence intervals respectively in Cox proportional-hazards regressions. The distribution of ticks on the x axis is exponential. Participants were categorized based on the data distribution of baseline meat intakes. Mean daily intakes in each category is calculated from the multiple 24-h dietary assessments which were used to test the linear trend per increment. Minimally-adjusted models adjusted for age, gender, ethnicity, education, socioeconomic status. Fully-adjusted models additionally adjusted for region, smoking status, physical activity, body mass index, sleep duration, stroke history, family history of dementia, dietary covariates including vegetables and fruits, total fish, tea and coffee, alcohol drinking; processed meat, unprocessed poultry, and unprocessed red meat were also mutually adjusted for.


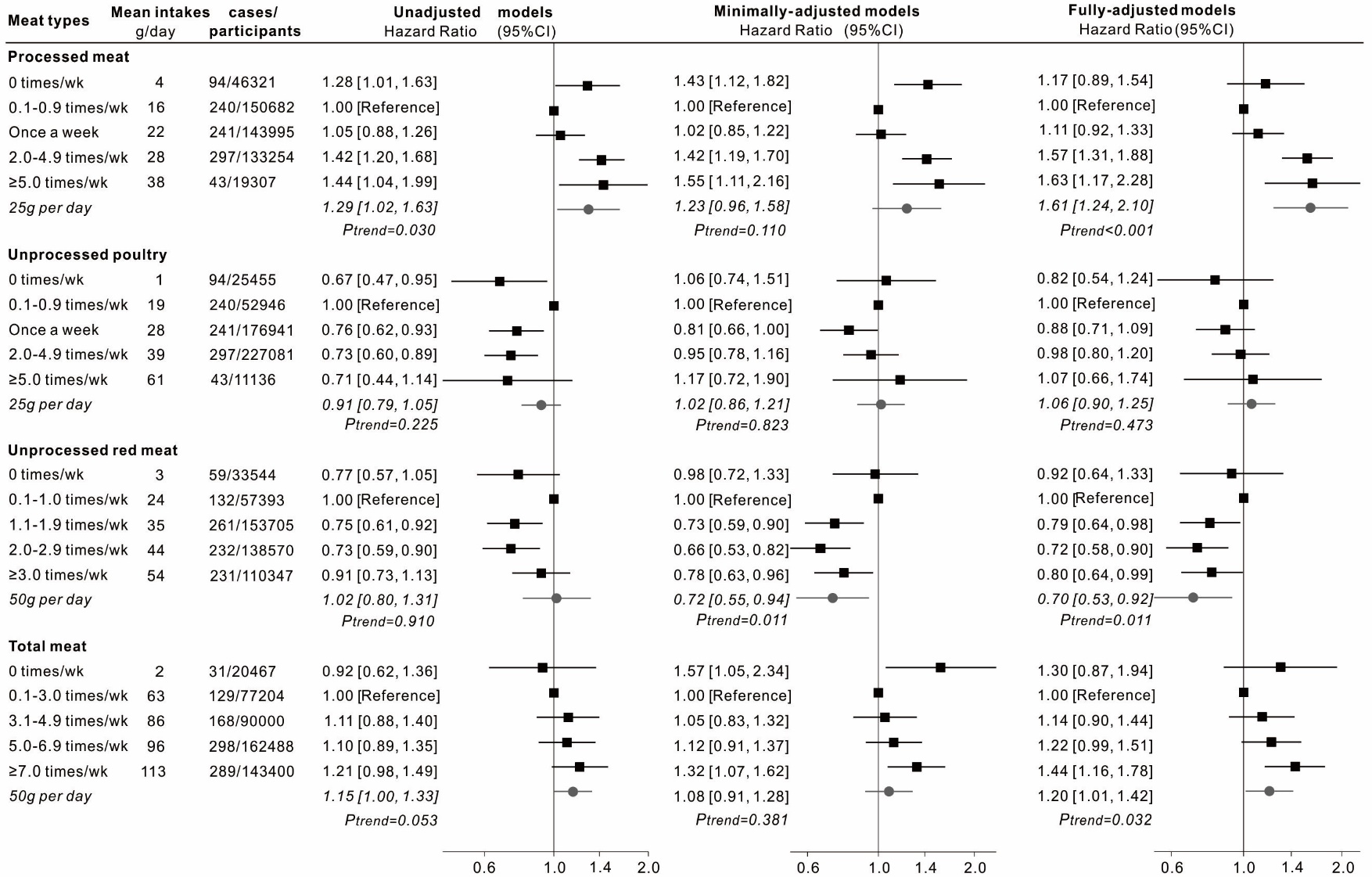


**Supplementary Figure 3 Hazard ratios (95% CIs) for the associations between incident Alzheimer’s disease and meat consumption with excluding cases arising in first 3 years of follow-up (n=493559)** The black squares and horizontal lines represent hazard ratios and 95% confidence intervals respectively in Cox proportional-hazards regressions. The distribution of ticks on the x axis is exponential. Participants were categorized based on the data distribution of baseline meat intakes. Mean daily intakes in each category is calculated from the multiple 24-h dietary assessments which were used to test the linear trend per increment. Minimally-adjusted models adjusted for age, gender, ethnicity, education, socioeconomic status. Fully-adjusted models additionally adjusted for region, smoking status, physical activity, body mass index, sleep duration, stroke history, family history of dementia, dietary covariates including vegetables and fruits, total fish, tea and coffee, alcohol drinking; processed meat, unprocessed poultry, and unprocessed red meat were also mutually adjusted for.


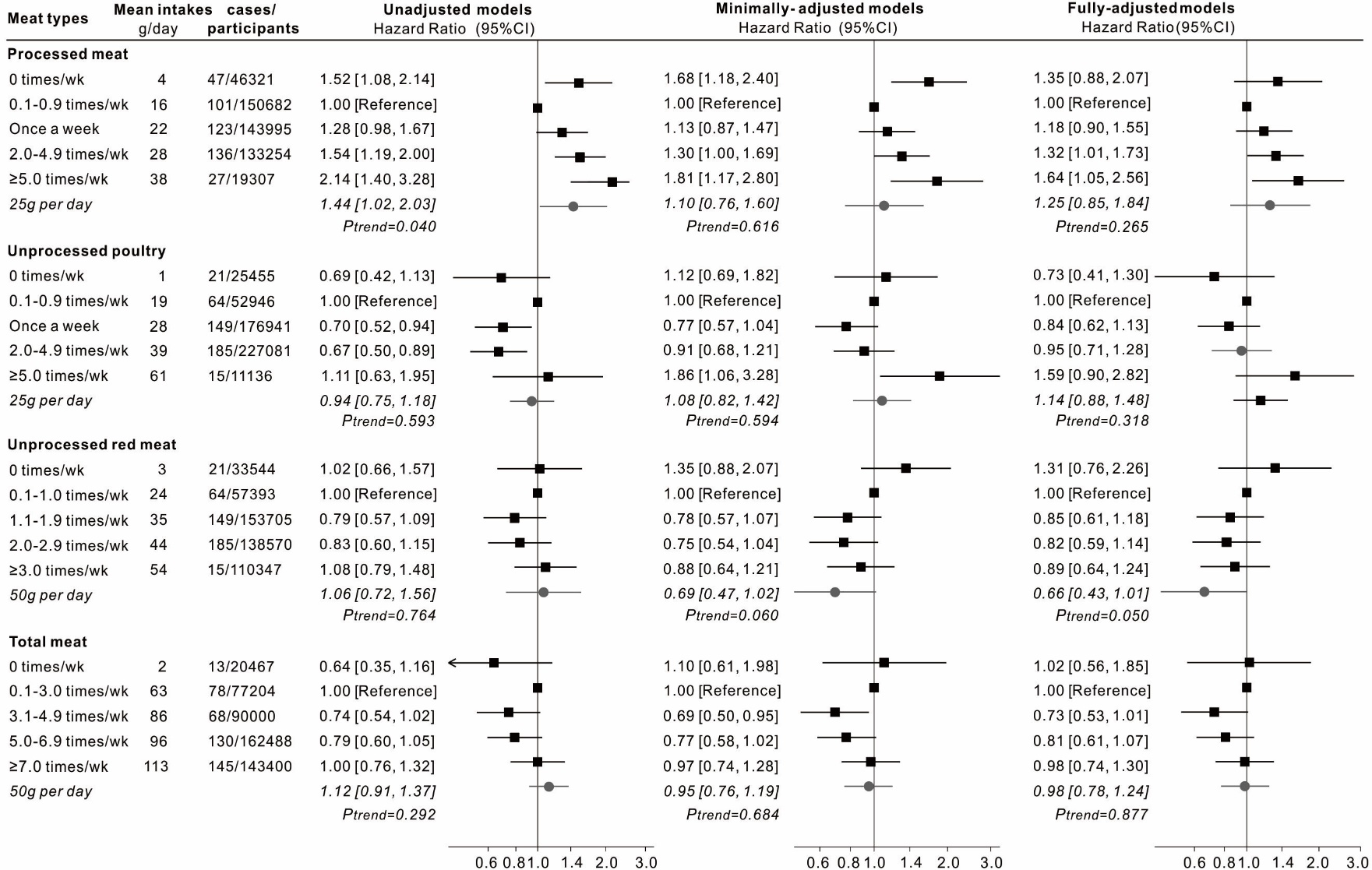


**Supplementary Figure 4 Hazard ratios (95% CIs) for the associations between incident vascular dementia and meat consumption with excluding cases arising in first 3 years of follow-up (n=493559)** The black squares and horizontal lines represent hazard ratios and 95% confidence intervals respectively in Cox proportional-hazards regressions. The distribution of ticks on the x axis is exponential. Participants were categorized based on the data distribution of baseline meat intakes. Mean daily intakes in each category is calculated from the multiple 24-h dietary assessments which were used to test the linear trend per increment. Minimally-adjusted models adjusted for age, gender, ethnicity, education, socioeconomic status. Fully-adjusted models additionally adjusted for region, smoking status, physical activity, body mass index, sleep duration, stroke history, family history of dementia, dietary covariates including vegetables and fruits, total fish, tea and coffee, alcohol drinking; processed meat, unprocessed poultry, and unprocessed red meat were also mutually adjusted for.


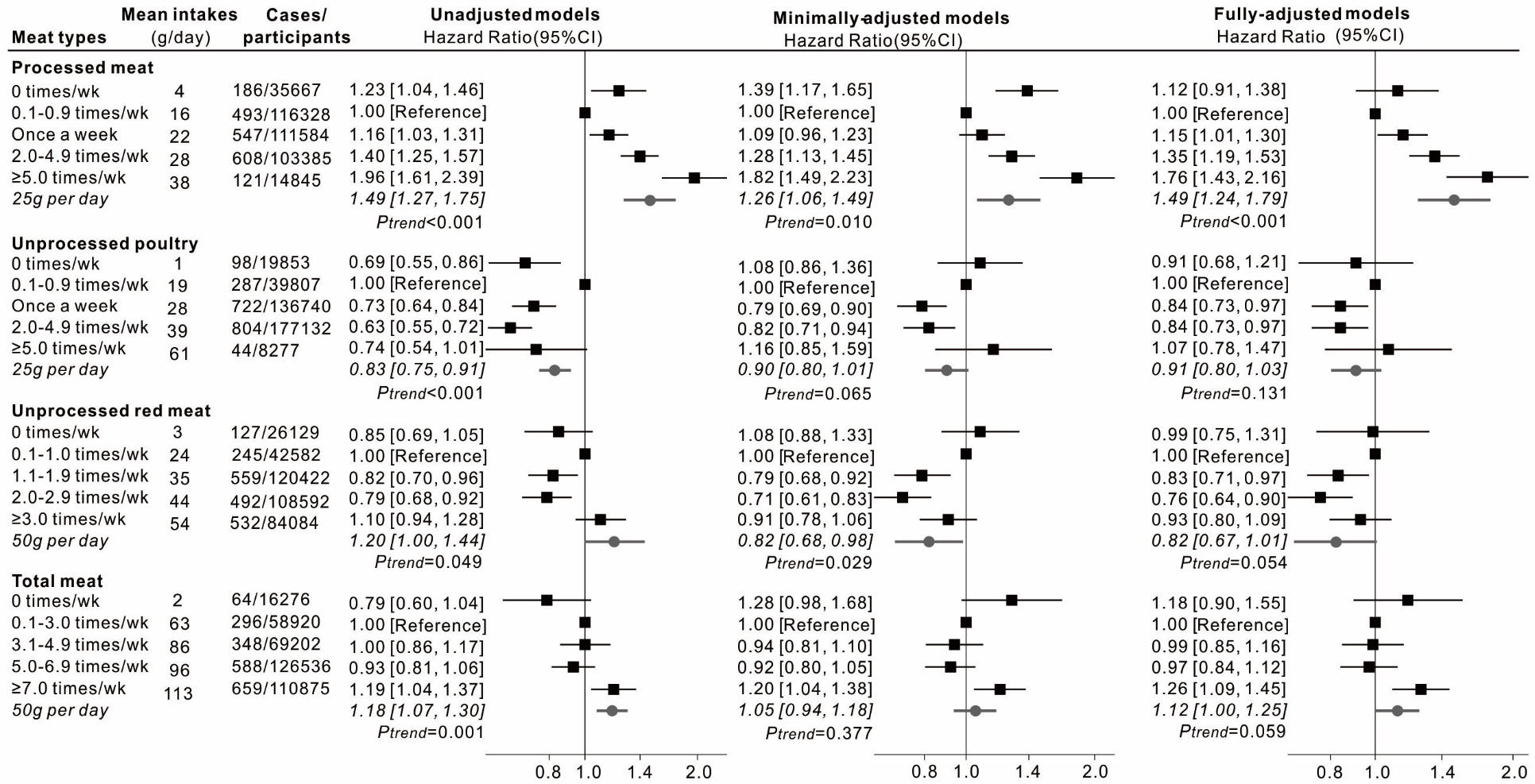


**Supplementary Figure 5 Hazard ratios (95% CIs) for the associations between incident all-cause dementia and meat consumption in participants with complete data on covariates (n=381809)** The black squares and horizontal lines represent hazard ratios and 95% confidence intervals respectively in Cox proportional-hazards regressions. The distribution of ticks on the x axis is exponential. Participants were categorized based on the data distribution of baseline meat intakes. Mean daily intakes in each category is calculated from the multiple 24-h dietary assessments which were used to test the linear trend per increment. Minimally-adjusted models adjusted for age, gender, ethnicity, education, socioeconomic status. Fully-adjusted models additionally adjusted for region, smoking status, physical activity, body mass index, sleep duration, stroke history, family history of dementia, dietary covariates including vegetables and fruits, total fish, tea and coffee, alcohol drinking; processed meat, unprocessed poultry, and unprocessed red meat were also mutually adjusted for.


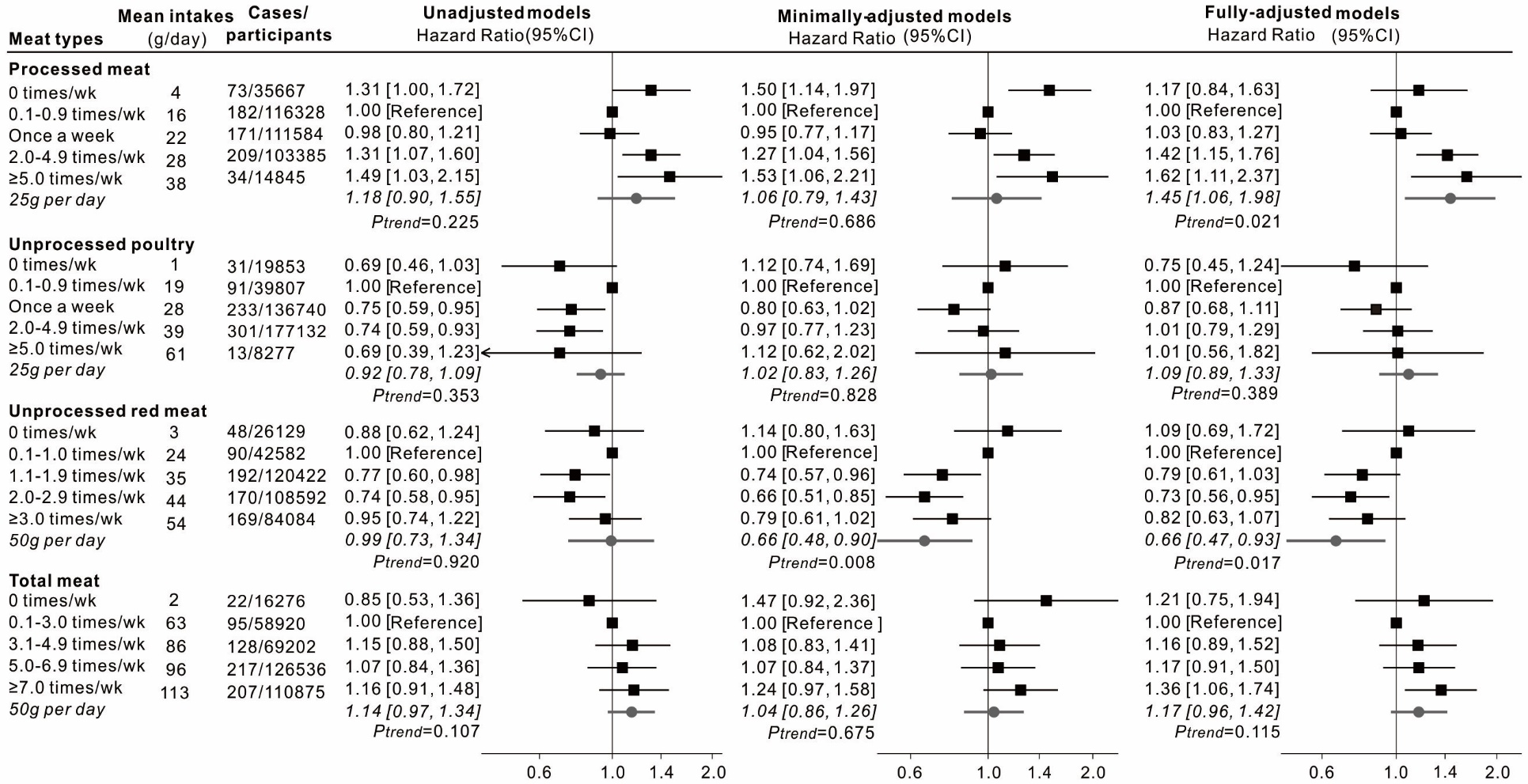


**Supplementary Figure 6 Hazard ratios (95% CIs) for the associations between incident Alzheimer’s disease and meat consumption in participants with complete data on covariates (n=381809)** The black squares and horizontal lines represent hazard ratios and 95% confidence intervals respectively in Cox proportional-hazards regressions. The distribution of ticks on the x axis is exponential. Participants were categorized based on the data distribution of baseline meat intakes. Mean daily intakes in each category is calculated from the multiple 24-h dietary assessments which were used to test the linear trend per increment. Minimally-adjusted models adjusted for age, gender, ethnicity, education, socioeconomic status. Fully-adjusted models additionally adjusted for region, smoking status, physical activity, body mass index, sleep duration, stroke history, family history of dementia, dietary covariates including vegetables and fruits, total fish, tea and coffee, alcohol drinking; processed meat, unprocessed poultry, and unprocessed red meat were also mutually adjusted for.


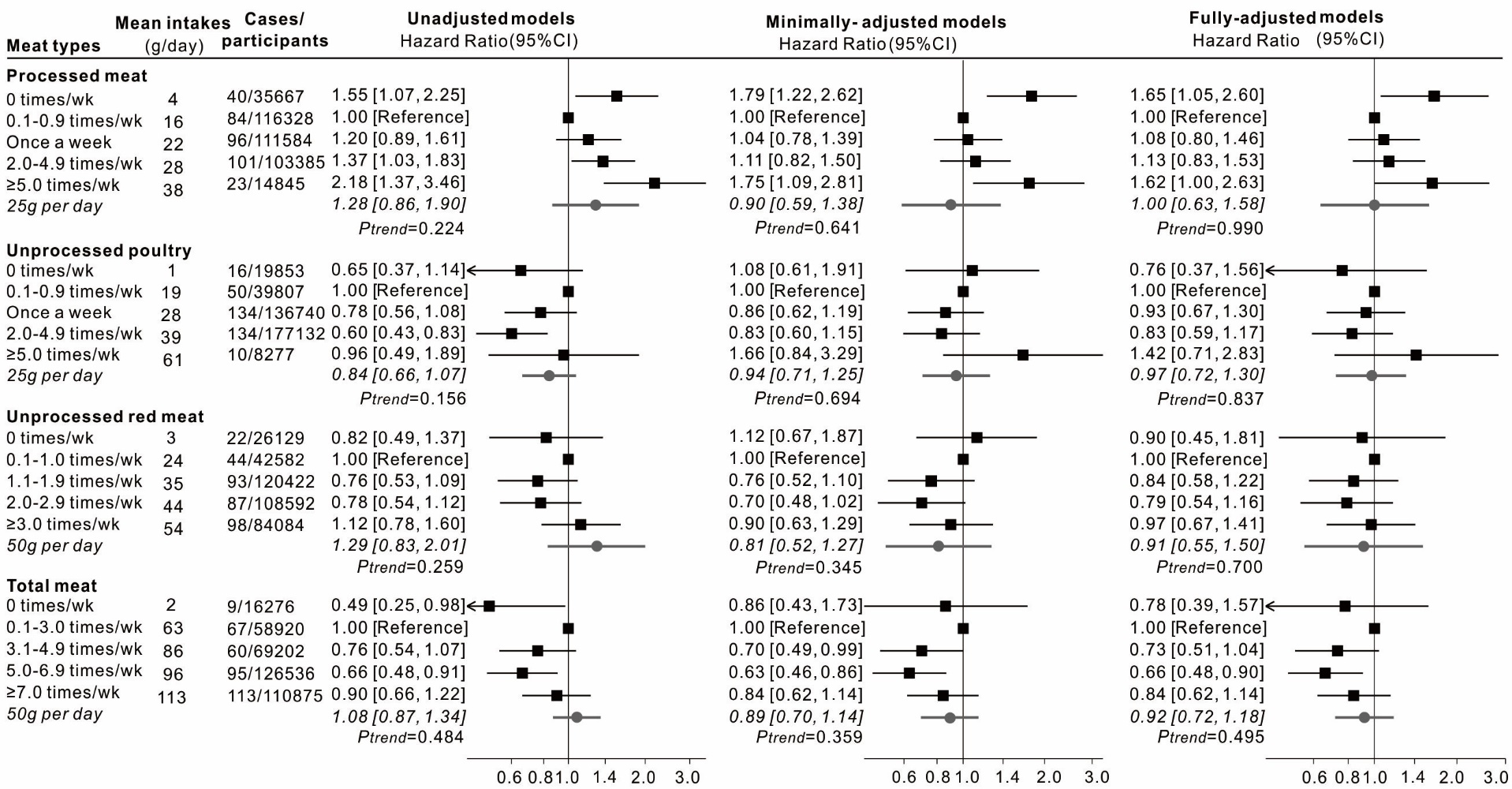


**Supplementary Figure 7 Hazard ratios (95% CIs) for the associations between incident vascular dementia and meat consumption in participants with complete data on covariates (n=381809)** The black squares and horizontal lines represent hazard ratios and 95% confidence intervals respectively in Cox proportional-hazards regressions. The distribution of ticks on the x axis is exponential. Participants were categorized based on the data distribution of baseline meat intakes. Mean daily intakes in each category is calculated from the multiple 24-h dietary assessments which were used to test the linear trend per increment. Minimally-adjusted models adjusted for age, gender, ethnicity, education, socioeconomic status. Fully-adjusted models additionally adjusted for region, smoking status, physical activity, body mass index, sleep duration, stroke history, family history of dementia, dietary covariates including vegetables and fruits, total fish, tea and coffee, alcohol drinking; processed meat, unprocessed poultry, and unprocessed red meat were also mutually adjusted for.


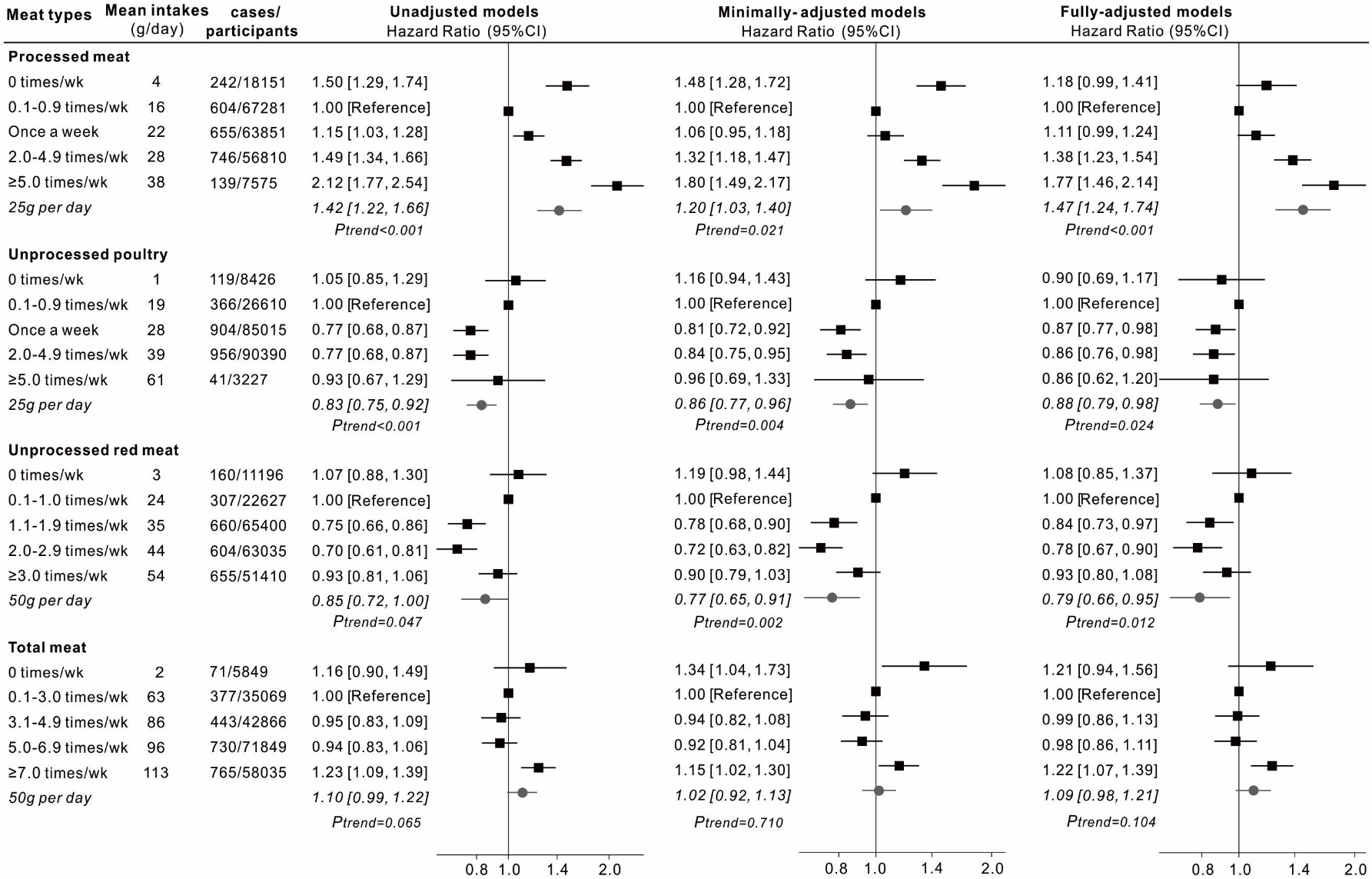


**Supplementary Figure 8 Hazard ratios (95% CIs) for the associations between incident all-cause dementia and meat consumption in participants aged 60 or more (n=213668)** The black squares and horizontal lines represent hazard ratios and 95% confidence intervals respectively in Cox proportional-hazards regressions. The distribution of ticks on the x axis is exponential. Participants were categorized based on the data distribution of baseline meat intakes. Mean daily intakes in each category is calculated from the multiple 24-h dietary assessments which were used to test the linear trend per increment. Minimally-adjusted models adjusted for age, gender, ethnicity, education, socioeconomic status. Fully-adjusted models additionally adjusted for region, smoking status, physical activity, body mass index, sleep duration, stroke history, family history of dementia, dietary covariates including vegetables and fruits, total fish, tea and coffee, alcohol drinking; processed meat, unprocessed poultry, and unprocessed red meat were also mutually adjusted for.


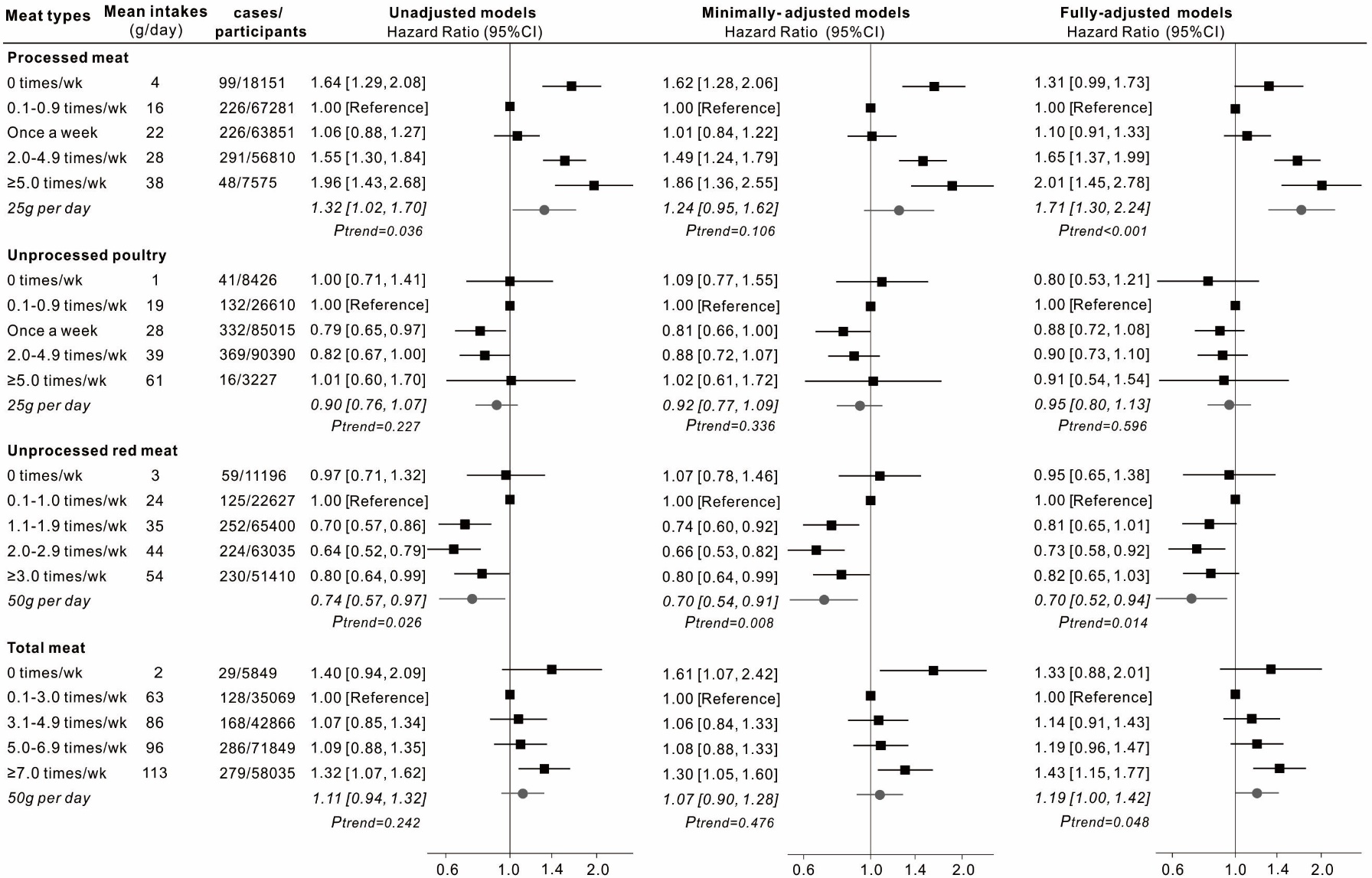


**Supplementary Figure 9 Hazard ratios (95% CIs) for the associations between incident Alzheimer’s disease and meat consumption** **in participants aged 60 or more (n=213668)** The black squares and horizontal lines represent hazard ratios and 95% confidence intervals respectively in Cox proportional-hazards regressions. The distribution of ticks on the x axis is exponential. Participants were categorized based on the data distribution of baseline meat intakes. Mean daily intakes in each category is calculated from the multiple 24-h dietary assessments which were used to test the linear trend per increment. Minimally-adjusted models adjusted for age, gender, ethnicity, education, socioeconomic status. Fully-adjusted models additionally adjusted for region, smoking status, physical activity, body mass index, sleep duration, stroke history, family history of dementia, dietary covariates including vegetables and fruits, total fish, tea and coffee, alcohol drinking; processed meat, unprocessed poultry, and unprocessed red meat were also mutually adjusted for.


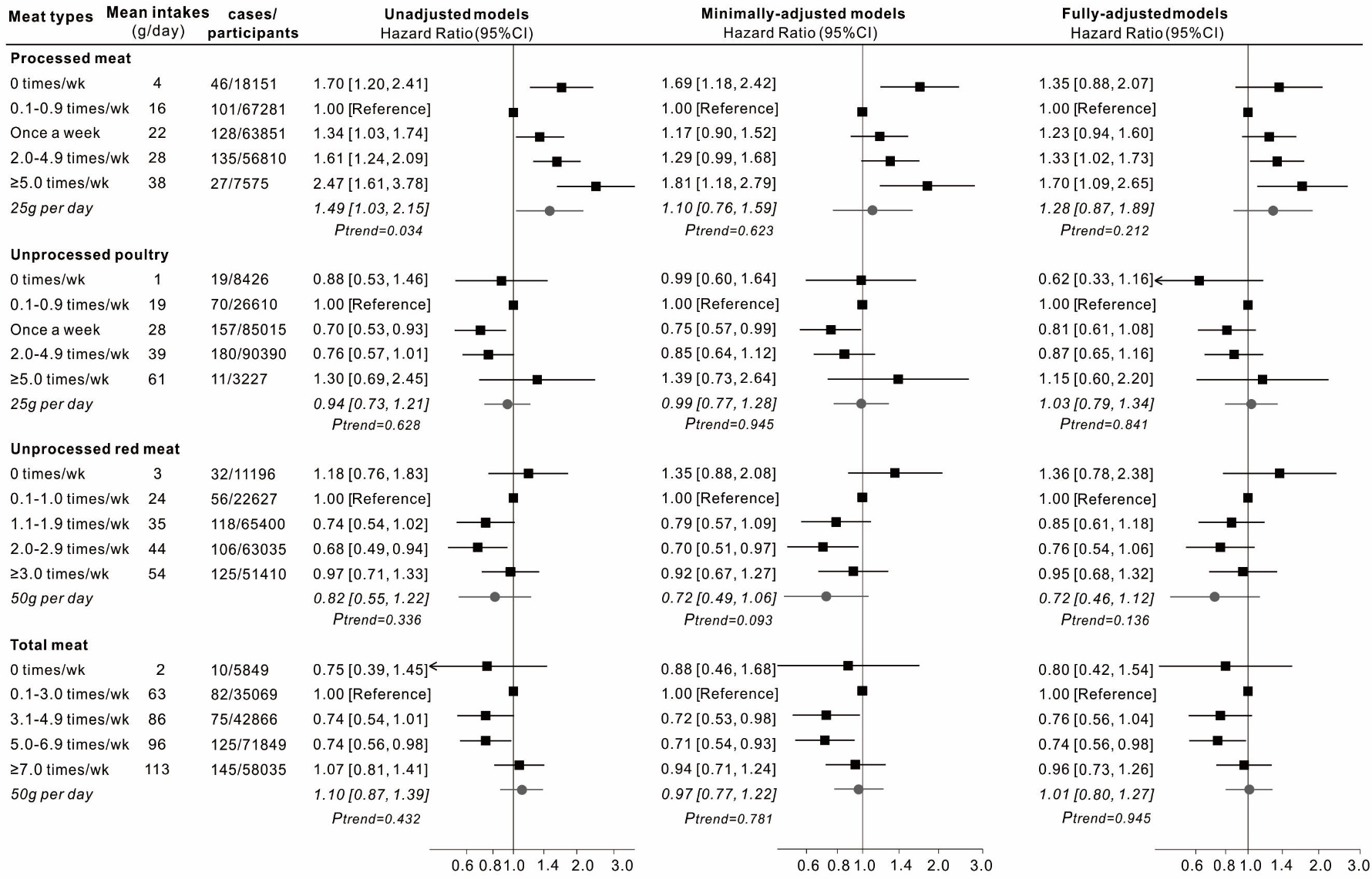


**Supplementary Figure 10 Hazard ratios (95% CIs) for the associations between incident vascular dementia and meat consumption in participants aged 60 or more (n=213668)** The black squares and horizontal lines represent hazard ratios and 95% confidence intervals respectively in Cox proportional-hazards regressions. The distribution of ticks on the x axis is exponential. Participants were categorized based on the data distribution of baseline meat intakes. Mean daily intakes in each category is calculated from the multiple 24-h dietary assessments which were used to test the linear trend per increment. Minimally-adjusted models adjusted for age, gender, ethnicity, education, socioeconomic status. Fully-adjusted models additionally adjusted for region, smoking status, physical activity, body mass index, sleep duration, stroke history, family history of dementia, dietary covariates including vegetables and fruits, total fish, tea and coffee, alcohol drinking; processed meat, unprocessed poultry, and unprocessed red meat were also mutually adjusted for.

# Supplementary Tables

## **Supplementary Table 1 Baseline characteristics of participants with various numbers of completions of 24-h dietary assessment in UK Biobank cohort study**

|  | **Response times to the Oxford WebQ** | | | |
| --- | --- | --- | --- | --- |
|  | Non responders | Once or more | Twice or more | At least three |
| Participants (n) | 291487 | 211006 | 126844 | 78725 |
| Age at baseline (years) | 56.9 (8.2) | 56.1 (7.9) | 56.1 (7.8) | 56.3 (7.7) |
| Gender |  |  |  |  |
| Men | 134348 (46.1%) | 94767 (44.9%) | 56128 (44.2%) | 34605 (44.0%) |
| Women | 157139 (53.9%) | 116239 (55.1%) | 70716 (55.8%) | 44120 (56.0%) |
| Ethnicity |  |  |  |  |
| White | 271408 (93.1%) | 201282 (95.4%) | 122439 (96.5%) | 76273 (96.9%) |
| Asian | 7881 (2.7%) | 3532 (1.7%) | 1519 (1.2%) | 796 (1.0%) |
| Black | 5423 (1.9%) | 2611 (1.2%) | 1018 (0.8%) | 510 (0.6%) |
| Mixed | 1757 (0.6%) | 1271 (0.6%) | 693 (0.5%) | 429 (0.5%) |
| Others/unknown | 1757 (0.6%) | 2310 (1.1%) | 1175 (0.9%) | 717 (0.9%) |
| Region |  |  |  |  |
| England | 251349 (86.2%) | 194496 (92.2%) | 116168 (91.6%) | 72185 (91.7%) |
| Wales | 14635 (5.0%) | 6172 (2.9%) | 3971 (3.1%) | 2403 (3.1%) |
| Scotland | 25503 (8.7%) | 10338 (4.9%) | 6705 (5.3%) | 4137 (5.3%) |
| Townsend deprivation index |  |  |  |  |
| Low deprivation | 92708 (31.8%) | 74668 (35.4%) | 45681 (36.0%) | 28198 (35.8%) |
| Moderate deprivation | 94685 (32.5%) | 72520 (34.4%) | 43777 (34.5%) | 27160 (34.5%) |
| High deprivation | 103736 (35.6%) | 63553 (30.1%) | 37237 (29.4%) | 23272 (29.6%) |
| Unknown | 358 (0.1%) | 265 (0.1%) | 149 (0.1%) | 95 (0.1%) |
| Educational level |  |  |  |  |
| Without college/university degree | 213440 (73.2%) | 120278 (57.0%) | 67026 (52.8%) | 40492 (51.4%) |
| With college/university degree | 72803 (25.0%) | 89759 (42.5%) | 59474 (46.9%) | 38047 (48.3%) |
| Unknown | 5244 (1.8%) | 969 (0.5%) | 344 (0.3%) | 186 (0.2%) |
| Smoking status |  |  |  |  |
| Never | 154521 (53.0%) | 119027 (56.4%) | 72401 (57.1%) | 45239 (57.5%) |
| Past | 98210 (33.7%) | 74923 (35.5%) | 45412 (35.8%) | 28225 (35.9%) |
| Current | 36427 (12.5%) | 16556 (7.8%) | 8808 (6.9%) | 5140 (6.5%) |
| Unknown | 2329 (0.8%) | 500 (0.2%) | 223 (0.2%) | 121 (0.2%) |
| Physical activity |  |  |  |  |
| Low level | 43279 (14.8%) | 32937 (15.6%) | 20147 (15.9%) | 12438 (15.8%) |
| Moderate level | 88401 (30.3%) | 75617 (35.8%) | 46909 (37.0%) | 29469 (37.4%) |
| High level | 91909 (31.5%) | 70229 (33.3%) | 41849 (33.0%) | 25971 (33.0%) |
| Unknown | 67898 (23.3%) | 32223 (15.3%) | 17939 (14.1%) | 10847 (13.8%) |
| Body mass index (BMI) |  |  |  |  |
| Normal/underweight (<25 Kg/m^2^) | 86527 (29.7%) | 78541 (37.2%) | 50079 (39.5%) | 32081 (40.8%) |
| Overweight (25-29.9 Kg/m^2^) | 124782 (42.8%) | 87365 (41.4%) | 51658 (40.7%) | 31593 (40.1%) |
| Obese (≥30 Kg/m^2^) | 77717 (26.7%) | 44556 (21.1%) | 24857 (19.6%) | 14899 (18.9%) |
| Unknown | 2461 (0.8%) | 544 (0.3%) | 250 (0.2%) | 152 (0.2%) |
| Sleep duration |  |  |  |  |
| <7 hours/day | 75349 (25.8%) | 47962 (22.7%) | 27446 (21.6%) | 16605 (21.1%) |
| 7-8 hours/day | 188033 (64.5%) | 148721 (70.5%) | 91224 (71.9%) | 57006 (72.4%) |
| >8 hours/day | 24687 (8.5%) | 13669 (6.5%) | 7911 (6.2%) | 4968 (6.3%) |
| Unknown | 3418 (1.2%) | 654 (0.3%) | 263 (0.2%) | 146 (0.2%) |
| With stroke history | 5360 (1.8%) | 2308 (1.1%) | 1262 (1.0%) | 741 (0.9%) |
| With family history of dementia | 30868 (10.6%) | 27558 (13.1%) | 16908 (13.3%) | 10820 (13.7%) |
| *APOE* ε4 carrying status |  |  |  |  |
| Non-carriers | 168917 (58.0%) | 126338 (59.9%) | 76229 (60.1%) | 47411 (60.2%) |
| Carriers | 68315 (23.4%) | 49541 (23.5%) | 29630 (23.4%) | 18381 (23.3%) |
| Missing | 54255 (18.6%) | 35127 (16.6%) | 20985 (16.5%) | 12933 (16.4%) |
| Total meat |  |  |  |  |
| Never | 9693 (3.3%) | 10808 (5.1%) | 7088 (5.6%) | 4647 (5.9%) |
| ≤3 times/week | 43414 (14.9%) | 33938 (16.1%) | 21116 (16.6%) | 13499 (17.1%) |
| 3-5 times/week | 52667 (18.1%) | 37502 (17.8%) | 22439 (17.7%) | 13941 (17.7%) |
| ≥5 times/week | 93630 (32.1%) | 69112 (32.8%) | 41204 (32.5%) | 25334 (32.2%) |
| ≥7 times/week | 85383 (29.3%) | 58354 (27.7%) | 34429 (27.1%) | 20996 (26.7%) |
| Vegetables/Fruits |  |  |  |  |
| <2 serving/day | 19660 (6.7%) | 10017 (4.7%) | 5464 (4.3%) | 3330 (4.2%) |
| <4 servings/day | 78883 (27.1%) | 56009 (26.5%) | 33116 (26.1%) | 20188 (25.6%) |
| 4-6 servings/day | 107799 (37.0%) | 84799 (40.2%) | 51790 (40.8%) | 32256 (41.0%) |
| >6 servings/day | 73135 (25.1%) | 56776 (26.9%) | 34868 (27.5%) | 22007 (28.0%) |
| Unknown | 12010 (4.1%) | 3405 (1.6%) | 1606 (1.3%) | 944 (1.2%) |
| Total fish |  |  |  |  |
| ≤1 times/week | 76434 (26.2%) | 52474 (24.9%) | 31052 (24.5%) | 19234 (24.4%) |
| 1-2 times/week | 59857 (20.5%) | 48445 (23.0%) | 29720 (23.4%) | 18574 (23.6%) |
| ≥2 times/week | 87150 (29.9%) | 64762 (30.7%) | 39078 (30.8%) | 24313 (30.9%) |
| ≥4 times/week | 63233 (21.7%) | 44389 (21.0%) | 26621 (21.0%) | 16405 (20.8%) |
| Unknown | 4813 (1.7%) | 936 (0.4%) | 373 (0.3%) | 199 (0.3%) |
| Alcohol |  |  |  |  |
| Less than once a week | 96892 (33.2%) | 57608 (27.3%) | 32830 (25.9%) | 20419 (25.9%) |
| Once or twice a week | 76840 (26.4%) | 52449 (24.9%) | 31114 (24.5%) | 19122 (24.3%) |
| Three or four times a week | 62811 (21.5%) | 52624 (24.9%) | 32608 (25.7%) | 20203 (25.7%) |
| Daily or almost daily | 53620 (18.4%) | 48148 (22.8%) | 30216 (23.8%) | 18942 (24.1%) |
| Unknown | 1324 (0.5%) | 177 (0.1%) | 76 (0.1%) | 39 (0.0%) |
| Tea/Coffee |  |  |  |  |
| ≤3 cups/day | 63286 (21.7%) | 47480 (22.5%) | 28897 (22.8%) | 18141 (23.0%) |
| ≤5 cups/day | 93364 (32.0%) | 70742 (33.5%) | 42869 (33.8%) | 26855 (34.1%) |
| ≤7 cups/day | 77355 (26.5%) | 56871 (27.0%) | 34301 (27.0%) | 21137 (26.8%) |
| >7 cups/day | 54850 (18.8%) | 35521 (16.8%) | 20616 (16.3%) | 12509 (15.9%) |
| Unknown | 2632 (0.9%) | 392 (0.2%) | 161 (0.1%) | 83 (0.1%) |
| **Dietary consumption from 24h WebQ** |  |  |  |  |
| **Processed meat** (g/d) | ─ | 20.5 (32.2) | 20.6 (26.5) | 20.6 (24.5) |
| Non-consumers | ─ | 109870 (52.1%) | 51029 (40.2%) | 27037 (34.3%) |
| Consumers | ─ |  |  |  |
| Mean (SD) | ─ | 42.7 (34.9) | 34.5 (26.3) | 31.4 (24) |
| Median (IQR) | ─ | 30 (20-60) | 27.5 (15-45) | 26.7 (13.3-40) |
| **Unprocessed poultry** (g/d) | ─ | 28.5 (46.3) | 28.3 (36.6) | 27.9 (33.1) |
| Non-consumers | ─ | 127844 (60.6%) | 63292 (49.9%) | 34703 (44.1%) |
| Consumers | ─ |  |  |  |
| Mean (SD) | ─ | 72.2 (47.6) | 56.5 (32.9) | 49.9 (29.2) |
| Median (IQR) | ─ | 60 (40-120) | 60 (30-60) | 40 (30-60) |
| **Unprocessed red meat** (g/d) | ─ | 38 (52.4) | 37.4 (41.6) | 37.2 (37.9) |
| Non-consumers | ─ | 110335 (52.3%) | 51844 (40.9%) | 27421 (34.8%) |
| Consumers | ─ |  |  |  |
| Mean (SD) | ─ | 79.6 (49.3) | 63.3 (36) | 57 (32.8) |
| Median (IQR) | ─ | 60 (40-120) | 60 (40-80) | 40 (30-80) |
| **Total meat** (g/d) | ─ | 86.9 (68.9) | 86.4 (57.9) | 85.7 (54.4) |
| Non-consumers | ─ | 40483 (19.2%) | 14347 (11.3%) | 7424 (9.4%) |
| Consumers | ─ |  |  |  |
| Mean (SD) | ─ | 107.6 (60.4) | 97.4 (52) | 94.6 (49.2) |
| Median (IQR) | ─ | 107.5 (60-125) | 90 (60-120) | 90 (60-120) |

## **Supplementary Table 2 Baseline characteristics of participants across categories of processed meat intakes in UK Biobank cohort study**

|  |  |  | **Processed meat** | (n=493,888) |  |
| --- | --- | --- | --- | --- | --- |
|  | 0 times/wk | 0.1-0.9 times/wk | Once a week | 2.0-4.9 times/wk | ≥5.0 times/wk |
| Participants (%) | 46358 (9%) | 150758 (31%) | 144076 (29%) | 133365 (27%) | 19331 (4%) |
| Age at baseline (years) | 55.9 (8.1) | 56.9 (7.9) | 56.7 (8.1) | 56.3 (8.2) | 55.6 (8.4) |
| Gender |  |  |  |  |  |
| Men | 12265 (26.5%) | 48180 (32.0%) | 66897 (46.4%) | 82866 (62.1%) | 14483 (74.9%) |
| Women | 34093 (73.5%) | 102578 (68.0%) | 77179 (53.6%) | 50499 (37.9%) | 4848 (25.1%) |
| Ethnicity |  |  |  |  |  |
| White | 40430 (87.2%) | 141456 (93.8%) | 138166 (95.9%) | 128433 (96.3%) | 18350 (94.9%) |
| Asian | 3281 (7.1%) | 3362 (2.2%) | 2060 (1.4%) | 1681 (1.3%) | 353 (1.8%) |
| Black | 1160 (2.5%) | 2900 (1.9%) | 1710 (1.2%) | 1414 (1.1%) | 270 (1.4%) |
| Mixed | 392 (0.8%) | 1012 (0.7%) | 778 (0.5%) | 668 (0.5%) | 101 (0.5%) |
| Others/unknown | 1095 (2.4%) | 2028 (1.3%) | 1362 (0.9%) | 1169 (0.9%) | 257 (1.3%) |
| Region |  |  |  |  |  |
| England | 41014 (88.5%) | 132308 (87.8%) | 127804 (88.7%) | 119691 (89.7%) | 17361 (89.8%) |
| Wales | 1832 (4.0%) | 6178 (4.1%) | 6190 (4.3%) | 5526 (4.1%) | 779 (4.0%) |
| Scotland | 3512 (7.6%) | 12272 (8.1%) | 10082 (7.0%) | 8148 (6.1%) | 1191 (6.2%) |
| Townsend deprivation index |  |  |  |  |  |
| Low deprivation | 13110 (28.3%) | 52041 (34.5%) | 50965 (35.4%) | 44112 (33.1%) | 5692 (29.4%) |
| Moderate deprivation | 14797 (31.9%) | 51043 (33.9%) | 48873 (33.9%) | 44413 (33.3%) | 5997 (31.0%) |
| High deprivation | 18393 (39.7%) | 47488 (31.5%) | 44051 (30.6%) | 44687 (33.5%) | 7616 (39.4%) |
| Unknown | 58 (0.1%) | 186 (0.1%) | 187 (0.1%) | 153 (0.1%) | 26 (0.1%) |
| Educational level |  |  |  |  |  |
| Without college/university degree | 26170 (56.5%) | 98085 (65.1%) | 97847 (67.9%) | 92379 (69.3%) | 13157 (68.1%) |
| With college/university degree | 19559 (42.2%) | 51341 (34.1%) | 44851 (31.1%) | 39763 (29.8%) | 5982 (30.9%) |
| Unknown | 629 (1.4%) | 1332 (0.9%) | 1378 (1.0%) | 1223 (0.9%) | 192 (1.0%) |
| Smoking status |  |  |  |  |  |
| Never | 27327 (58.9%) | 85447 (56.7%) | 78802 (54.7%) | 68768 (51.6%) | 9255 (47.9%) |
| Past | 15124 (32.6%) | 51769 (34.3%) | 50196 (34.8%) | 47240 (35.4%) | 6612 (34.2%) |
| Current | 3712 (8.0%) | 13100 (8.7%) | 14547 (10.1%) | 16967 (12.7%) | 3408 (17.6%) |
| Unknown | 195 (0.4%) | 442 (0.3%) | 531 (0.4%) | 390 (0.3%) | 56 (0.3%) |
| Physical activity |  |  |  |  |  |
| Low level | 5965 (12.9%) | 22016 (14.6%) | 22324 (15.5%) | 21634 (16.2%) | 3396 (17.6%) |
| Moderate level | 14709 (31.7%) | 49888 (33.1%) | 48102 (33.4%) | 43813 (32.9%) | 6076 (31.4%) |
| High level | 17232 (37.2%) | 49608 (32.9%) | 45666 (31.7%) | 42155 (31.6%) | 6123 (31.7%) |
| Unknown | 8452 (18.2%) | 29246 (19.4%) | 27984 (19.4%) | 25763 (19.3%) | 3736 (19.3%) |
| Body mass index (BMI) |  |  |  |  |  |
| Normal/underweight (<25 Kg/m2) | 22042 (47.5%) | 54846 (36.4%) | 44773 (31.1%) | 35917 (26.9%) | 5328 (27.6%) |
| Overweight (25-29.9 Kg/m2) | 16470 (35.5%) | 62408 (41.4%) | 62952 (43.7%) | 58688 (44.0%) | 8294 (42.9%) |
| Obese (≥30 Kg/m2) | 7462 (16.1%) | 32841 (21.8%) | 35747 (24.8%) | 38070 (28.5%) | 5582 (28.9%) |
| Unknown | 384 (0.8%) | 663 (0.4%) | 604 (0.4%) | 690 (0.5%) | 127 (0.7%) |
| Sleep duration |  |  |  |  |  |
| <7 hours/day | 12150 (26.2%) | 36468 (24.2%) | 33777 (23.4%) | 33146 (24.9%) | 5446 (28.2%) |
| 7-8 hours/day | 30352 (65.5%) | 102400 (67.9%) | 98689 (68.5%) | 89221 (66.9%) | 12190 (63.1%) |
| >8 hours/day | 3553 (7.7%) | 11142 (7.4%) | 10953 (7.6%) | 10338 (7.8%) | 1578 (8.2%) |
| Unknown | 303 (0.7%) | 748 (0.5%) | 657 (0.5%) | 660 (0.5%) | 117 (0.6%) |
| With stroke history | 622 (1.3%) | 1992 (1.3%) | 2182 (1.5%) | 2215 (1.7%) | 386 (2.0%) |
| With family history of dementia | 5386 (11.6%) | 18136 (12.0%) | 16637 (11.5%) | 15310 (11.5%) | 2259 (11.7%) |
| *APOE* ε4 carrying status |  |  |  |  |  |
| Non-carriers | 27495 (59.3%) | 88948 (59.0%) | 84889 (58.9%) | 77768 (58.3%) | 11282 (58.4%) |
| Carriers | 11264 (24.3%) | 35666 (23.7%) | 33624 (23.3%) | 30966 (23.2%) | 4353 (22.5%) |
| Missing | 7599 (16.4%) | 26144 (17.3%) | 25563 (17.7%) | 24631 (18.5%) | 3696 (19.1%) |
| Total meat |  |  |  |  |  |
| Never | 20473 (44.2%) | 0 (0.0%) | 0 (0.0%) | 0 (0.0%) | 0 (0.0%) |
| ≤3 times/week | 13873 (29.9%) | 49740 (33.0%) | 13489 (9.4%) | 159 (0.1%) | 0 (0.0%) |
| 3-5 times/week | 6861 (14.8%) | 35096 (23.3%) | 45028 (31.3%) | 3080 (2.3%) | 0 (0.0%) |
| ≥5 times/week | 3542 (7.6%) | 52064 (34.5%) | 62328 (43.3%) | 44422 (33.3%) | 214 (1.1%) |
| ≥7 times/week | 1609 (3.5%) | 13858 (9.2%) | 23231 (16.1%) | 85704 (64.3%) | 19117 (98.9%) |
| Vegetables/Fruits |  |  |  |  |  |
| <2 serving/day | 1309 (2.8%) | 5869 (3.9%) | 7983 (5.5%) | 11066 (8.3%) | 2733 (14.1%) |
| <4 servings/day | 7290 (15.7%) | 33820 (22.4%) | 41863 (29.1%) | 43716 (32.8%) | 6661 (34.5%) |
| 4-6 servings/day | 16410 (35.4%) | 61263 (40.6%) | 57479 (39.9%) | 49684 (37.3%) | 6017 (31.1%) |
| >6 servings/day | 20177 (43.5%) | 46296 (30.7%) | 33345 (23.1%) | 25349 (19.0%) | 3320 (17.2%) |
| Unknown | 1172 (2.5%) | 3510 (2.3%) | 3406 (2.4%) | 3550 (2.7%) | 600 (3.1%) |
| Total fish |  |  |  |  |  |
| ≤1 times/week | 16525 (35.6%) | 36704 (24.3%) | 33181 (23.0%) | 33810 (25.4%) | 6760 (35.0%) |
| 1-2 times/week | 5053 (10.9%) | 33321 (22.1%) | 32970 (22.9%) | 31312 (23.5%) | 4563 (23.6%) |
| ≥2 times/week | 10749 (23.2%) | 45380 (30.1%) | 47628 (33.1%) | 41619 (31.2%) | 4824 (25.0%) |
| ≥4 times/week | 13730 (29.6%) | 34469 (22.9%) | 29413 (20.4%) | 25737 (19.3%) | 2982 (15.4%) |
| Unknown | 301 (0.6%) | 884 (0.6%) | 884 (0.6%) | 887 (0.7%) | 202 (1.0%) |
| Alcohol |  |  |  |  |  |
| Less than once a week | 20274 (43.7%) | 49730 (33.0%) | 40575 (28.2%) | 34602 (25.9%) | 5394 (27.9%) |
| Once or twice a week | 10267 (22.1%) | 39529 (26.2%) | 39233 (27.2%) | 33966 (25.5%) | 4534 (23.5%) |
| Three or four times a week | 8605 (18.6%) | 33208 (22.0%) | 34745 (24.1%) | 33712 (25.3%) | 4231 (21.9%) |
| Daily or almost daily | 7166 (15.5%) | 28199 (18.7%) | 29436 (20.4%) | 30998 (23.2%) | 5145 (26.6%) |
| Unknown | 46 (0.1%) | 92 (0.1%) | 87 (0.1%) | 87 (0.1%) | 27 (0.1%) |
| Tea/Coffee |  |  |  |  |  |
| ≤3 cups/day | 14568 (31.4%) | 36280 (24.1%) | 29135 (20.2%) | 24986 (18.7%) | 3867 (20.0%) |
| ≤5 cups/day | 14184 (30.6%) | 50759 (33.7%) | 48588 (33.7%) | 42842 (32.1%) | 5592 (28.9%) |
| ≤7 cups/day | 10186 (22.0%) | 39242 (26.0%) | 40272 (28.0%) | 37896 (28.4%) | 5064 (26.2%) |
| >7 cups/day | 7216 (15.6%) | 24102 (16.0%) | 25714 (17.8%) | 27224 (20.4%) | 4731 (24.5%) |
| Unknown | 204 (0.4%) | 375 (0.2%) | 367 (0.3%) | 417 (0.3%) | 77 (0.4%) |
| Energy intakes (KJ/day) | 8389 (2466) | 8404 (2163) | 8852 (2197) | 9417 (2326) | 9889 (2611) |
| Protein intakes (g/day) | 72.6 (22) | 80 (20.7) | 83.5 (21) | 87.3 (21.9) | 91.7 (24.8) |
| Fat intakes (g/day) | 72.4 (27.1) | 73 (24.5) | 78.8 (25.4) | 85.2 (27.1) | 91.4 (31.7) |
| Saturated fat intakes (g/day) | 27.1 (12.3) | 28 (10.8) | 30.4 (11.2) | 33 (11.9) | 35.4 (13.7) |
| Carbohydrate intakes (g/day) | 257.5 (89.9) | 244.9 (75.5) | 253.3 (75.1) | 263.4 (76.8) | 273 (87.1) |
| Iron intakes (mg/day) | 14 (4.2) | 13.4 (3.7) | 13.7 (3.7) | 14.1 (3.8) | 14.5 (4.1) |

## **Supplementary Table 3 Baseline characteristics of participants across categories of unprocessed poultry intakes in UK Biobank cohort study**

|  |  | **Unprocessed poultry** (n=493,888) | | |  |
| --- | --- | --- | --- | --- | --- |
|  | 0 times/wk | 0.1-0.9 times/wk | Once a week | 2.0-4.9 times/wk | ≥5.0 times/wk |
| Participants (%) | 25471 (5%) | 53001 (11%) | 177074 (36%) | 227200 (46%) | 11142 (2%) |
| Age at baseline (years) | 54.6 (8.2) | 57.9 (7.9) | 57.4 (7.9) | 55.9 (8.1) | 53.6 (8.3) |
| Gender |  |  |  |  |  |
| Men | 8778 (34.5%) | 25422 (48.0%) | 83126 (46.9%) | 102311 (45.0%) | 5054 (45.4%) |
| Women | 16693 (65.5%) | 27579 (52.0%) | 93948 (53.1%) | 124889 (55.0%) | 6088 (54.6%) |
| Ethnicity |  |  |  |  |  |
| White | 22507 (88.4%) | 50301 (94.9%) | 169706 (95.8%) | 214932 (94.6%) | 9389 (84.3%) |
| Asian | 2048 (8.0%) | 1161 (2.2%) | 2896 (1.6%) | 4131 (1.8%) | 501 (4.5%) |
| Black | 303 (1.2%) | 577 (1.1%) | 1808 (1.0%) | 3974 (1.7%) | 792 (7.1%) |
| Mixed | 218 (0.9%) | 300 (0.6%) | 876 (0.5%) | 1444 (0.6%) | 113 (1.0%) |
| Others/unknown | 395 (1.6%) | 662 (1.2%) | 1788 (1.0%) | 2719 (1.2%) | 347 (3.1%) |
| Region |  |  |  |  |  |
| England | 22943 (90.1%) | 47232 (89.1%) | 157485 (88.9%) | 200654 (88.3%) | 9864 (88.5%) |
| Wales | 994 (3.9%) | 2215 (4.2%) | 7255 (4.1%) | 9593 (4.2%) | 448 (4.0%) |
| Scotland | 1534 (6.0%) | 3554 (6.7%) | 12334 (7.0%) | 16953 (7.5%) | 830 (7.4%) |
| Townsend deprivation index |  |  |  |  |  |
| Low deprivation | 6601 (25.9%) | 15112 (28.5%) | 61500 (34.7%) | 79624 (35.0%) | 3083 (27.7%) |
| Moderate deprivation | 8092 (31.8%) | 17320 (32.7%) | 59620 (33.7%) | 76760 (33.8%) | 3331 (29.9%) |
| High deprivation | 10743 (42.2%) | 20516 (38.7%) | 55774 (31.5%) | 70494 (31.0%) | 4708 (42.3%) |
| Unknown | 35 (0.1%) | 53 (0.1%) | 180 (0.1%) | 322 (0.1%) | 20 (0.2%) |
| Educational level |  |  |  |  |  |
| Without college/university degree | 13175 (51.7%) | 33257 (62.7%) | 118624 (67.0%) | 154906 (68.2%) | 7676 (68.9%) |
| With college/university degree | 11999 (47.1%) | 19180 (36.2%) | 56700 (32.0%) | 70283 (30.9%) | 3334 (29.9%) |
| Unknown | 297 (1.2%) | 564 (1.1%) | 1750 (1.0%) | 2011 (0.9%) | 132 (1.2%) |
| Smoking status |  |  |  |  |  |
| Never | 14526 (57.0%) | 26978 (50.9%) | 95483 (53.9%) | 126322 (55.6%) | 6290 (56.5%) |
| Past | 8454 (33.2%) | 18845 (35.6%) | 61639 (34.8%) | 78497 (34.5%) | 3506 (31.5%) |
| Current | 2383 (9.4%) | 6984 (13.2%) | 19342 (10.9%) | 21716 (9.6%) | 1309 (11.7%) |
| Unknown | 108 (0.4%) | 194 (0.4%) | 610 (0.3%) | 665 (0.3%) | 37 (0.3%) |
| Physical activity |  |  |  |  |  |
| Low level | 3407 (13.4%) | 8243 (15.6%) | 27348 (15.4%) | 34567 (15.2%) | 1770 (15.9%) |
| Moderate level | 8314 (32.6%) | 17200 (32.5%) | 59429 (33.6%) | 74428 (32.8%) | 3217 (28.9%) |
| High level | 9171 (36.0%) | 16531 (31.2%) | 55834 (31.5%) | 75345 (33.2%) | 3903 (35.0%) |
| Unknown | 4579 (18.0%) | 11027 (20.8%) | 34463 (19.5%) | 42860 (18.9%) | 2252 (20.2%) |
| Body mass index (BMI) |  |  |  |  |  |
| Normal/underweight (<25 Kg/m2) | 12273 (48.2%) | 20050 (37.8%) | 59814 (33.8%) | 67975 (29.9%) | 2794 (25.1%) |
| Overweight (25-29.9 Kg/m2) | 8884 (34.9%) | 21389 (40.4%) | 76158 (43.0%) | 97745 (43.0%) | 4636 (41.6%) |
| Obese (≥30 Kg/m2) | 4094 (16.1%) | 11250 (21.2%) | 40216 (22.7%) | 60495 (26.6%) | 3647 (32.7%) |
| Unknown | 220 (0.9%) | 312 (0.6%) | 886 (0.5%) | 985 (0.4%) | 65 (0.6%) |
| Sleep duration |  |  |  |  |  |
| <7 hours/day | 6613 (26.0%) | 13269 (25.0%) | 41643 (23.5%) | 56144 (24.7%) | 3318 (29.8%) |
| 7-8 hours/day | 16861 (66.2%) | 34827 (65.7%) | 120782 (68.2%) | 153532 (67.6%) | 6850 (61.5%) |
| >8 hours/day | 1818 (7.1%) | 4505 (8.5%) | 13797 (7.8%) | 16556 (7.3%) | 888 (8.0%) |
| Unknown | 179 (0.7%) | 400 (0.8%) | 852 (0.5%) | 968 (0.4%) | 86 (0.8%) |
| With stroke history | 300 (1.2%) | 952 (1.8%) | 2731 (1.5%) | 3227 (1.4%) | 187 (1.7%) |
| With family history of dementia | 2834 (11.1%) | 6551 (12.4%) | 21148 (11.9%) | 26093 (11.5%) | 1102 (9.9%) |
| *APOE* ε4 carrying status |  |  |  |  |  |
| Non-carriers | 15154 (59.5%) | 31155 (58.8%) | 104224 (58.9%) | 133469 (58.7%) | 6380 (57.3%) |
| Carriers | 6144 (24.1%) | 12300 (23.2%) | 41215 (23.3%) | 53452 (23.5%) | 2762 (24.8%) |
| Missing | 4173 (16.4%) | 9546 (18.0%) | 31635 (17.9%) | 40279 (17.7%) | 2000 (18.0%) |
| Total meat |  |  |  |  |  |
| Never | 20473 (80.4%) | 0 (0.0%) | 0 (0.0%) | 0 (0.0%) | 0 (0.0%) |
| ≤3 times/week | 2775 (10.9%) | 29906 (56.4%) | 42602 (24.1%) | 1978 (0.9%) | 0 (0.0%) |
| 3-5 times/week | 755 (3.0%) | 7173 (13.5%) | 62541 (35.3%) | 19596 (8.6%) | 0 (0.0%) |
| ≥5 times/week | 745 (2.9%) | 11088 (20.9%) | 49764 (28.1%) | 100019 (44.0%) | 954 (8.6%) |
| ≥7 times/week | 723 (2.8%) | 4834 (9.1%) | 22167 (12.5%) | 105607 (46.5%) | 10188 (91.4%) |
| Vegetables/Fruits |  |  |  |  |  |
| <2 serving/day | 1132 (4.4%) | 4834 (9.1%) | 10793 (6.1%) | 11402 (5.0%) | 799 (7.2%) |
| <4 servings/day | 4666 (18.3%) | 14509 (27.4%) | 51523 (29.1%) | 59971 (26.4%) | 2681 (24.1%) |
| 4-6 servings/day | 9104 (35.7%) | 18638 (35.2%) | 68968 (38.9%) | 90387 (39.8%) | 3756 (33.7%) |
| >6 servings/day | 9894 (38.8%) | 13229 (25.0%) | 41199 (23.3%) | 60631 (26.7%) | 3534 (31.7%) |
| Unknown | 675 (2.7%) | 1791 (3.4%) | 4591 (2.6%) | 4809 (2.1%) | 372 (3.3%) |
| Total fish |  |  |  |  |  |
| ≤1 times/week | 13348 (52.4%) | 17732 (33.5%) | 42158 (23.8%) | 50307 (22.1%) | 3435 (30.8%) |
| 1-2 times/week | 2095 (8.2%) | 11885 (22.4%) | 42485 (24.0%) | 48782 (21.5%) | 1972 (17.7%) |
| ≥2 times/week | 4126 (16.2%) | 13475 (25.4%) | 58044 (32.8%) | 71784 (31.6%) | 2771 (24.9%) |
| ≥4 times/week | 5771 (22.7%) | 9400 (17.7%) | 33254 (18.8%) | 55067 (24.2%) | 2839 (25.5%) |
| Unknown | 131 (0.5%) | 509 (1.0%) | 1133 (0.6%) | 1260 (0.6%) | 125 (1.1%) |
| Alcohol |  |  |  |  |  |
| Less than once a week | 10728 (42.1%) | 18209 (34.4%) | 50816 (28.7%) | 66163 (29.1%) | 4659 (41.8%) |
| Once or twice a week | 5420 (21.3%) | 12615 (23.8%) | 46648 (26.3%) | 60264 (26.5%) | 2582 (23.2%) |
| Three or four times a week | 5039 (19.8%) | 10991 (20.7%) | 41588 (23.5%) | 54871 (24.2%) | 2012 (18.1%) |
| Daily or almost daily | 4249 (16.7%) | 11140 (21.0%) | 37898 (21.4%) | 45781 (20.2%) | 1876 (16.8%) |
| Unknown | 35 (0.1%) | 46 (0.1%) | 124 (0.1%) | 121 (0.1%) | 13 (0.1%) |
| Tea/Coffee |  |  |  |  |  |
| ≤3 cups/day | 7616 (29.9%) | 12195 (23.0%) | 37062 (20.9%) | 48860 (21.5%) | 3103 (27.8%) |
| ≤5 cups/day | 7516 (29.5%) | 16847 (31.8%) | 59803 (33.8%) | 74574 (32.8%) | 3225 (28.9%) |
| ≤7 cups/day | 5894 (23.1%) | 13548 (25.6%) | 48399 (27.3%) | 62318 (27.4%) | 2501 (22.4%) |
| >7 cups/day | 4311 (16.9%) | 10224 (19.3%) | 31267 (17.7%) | 40937 (18.0%) | 2248 (20.2%) |
| Unknown | 134 (0.5%) | 187 (0.4%) | 543 (0.3%) | 511 (0.2%) | 65 (0.6%) |
| Energy intakes (KJ/day) | 8669 (2482) | 8831 (2355) | 8842 (2223) | 8861 (2322) | 9122 (2732) |
| Protein intakes (g/day) | 69.2 (20.8) | 78.4 (21.4) | 82.2 (20.8) | 85.3 (21.7) | 90.9 (29.5) |
| Fat intakes (g/day) | 76.2 (28) | 78.6 (26.8) | 78.8 (26.2) | 78.2 (26.5) | 78 (31) |
| Saturated fat intakes (g/day) | 28.7 (12.9) | 30.6 (12.1) | 30.5 (11.6) | 29.9 (11.5) | 29.4 (13) |
| Carbohydrate intakes (g/day) | 268.8 (91.5) | 255.9 (78.5) | 253.3 (76.3) | 252.6 (76.8) | 252.4 (90.1) |
| Iron intakes (mg/day) | 14.3 (4.3) | 13.7 (3.9) | 13.8 (3.7) | 13.7 (3.8) | 13.5 (4.4) |

## **Supplementary Table 4 Baseline characteristics of participants across categories of unprocessed red meat intakes in UK Biobank cohort study**

|  |  | **Unprocessed red meat** (n=493,888) | | |  |
| --- | --- | --- | --- | --- | --- |
|  | 0 times/wk | 0.1-1.0 times/wk | 1.1-1.9 times/wk | 2.0-2.9 times/wk | ≥3.0 times/wk |
| Participants (%) | 33569 (7%) | 57433 (12%) | 153797 (31%) | 138648 (28%) | 110441 (22%) |
| Age at baseline (years) | 54.6 (8.2) | 55.7 (8.3) | 56.5 (8.0) | 56.9 (8.0) | 57.1 (8.0) |
| Gender |  |  |  |  |  |
| Men | 10359 (30.9%) | 21133 (36.8%) | 69590 (45.2%) | 65875 (47.5%) | 57734 (52.3%) |
| Women | 23210 (69.1%) | 36300 (63.2%) | 84207 (54.8%) | 72773 (52.5%) | 52707 (47.7%) |
| Ethnicity |  |  |  |  |  |
| White | 29744 (88.6%) | 51815 (90.2%) | 148348 (96.5%) | 133918 (96.6%) | 103010 (93.3%) |
| Asian | 2547 (7.6%) | 2949 (5.1%) | 1483 (1.0%) | 1224 (0.9%) | 2534 (2.3%) |
| Black | 501 (1.5%) | 1246 (2.2%) | 1806 (1.2%) | 1561 (1.1%) | 2340 (2.1%) |
| Mixed | 266 (0.8%) | 382 (0.7%) | 827 (0.5%) | 729 (0.5%) | 747 (0.7%) |
| Others/unknown | 511 (1.5%) | 1041 (1.8%) | 1333 (0.9%) | 1216 (0.9%) | 1810 (1.6%) |
| Region |  |  |  |  |  |
| England | 30127 (89.7%) | 50451 (87.8%) | 139093 (90.4%) | 123268 (88.9%) | 95239 (86.2%) |
| Wales | 1379 (4.1%) | 2395 (4.2%) | 7181 (4.7%) | 5818 (4.2%) | 3732 (3.4%) |
| Scotland | 2063 (6.1%) | 4587 (8.0%) | 7523 (4.9%) | 9562 (6.9%) | 11470 (10.4%) |
| Townsend deprivation index |  |  |  |  |  |
| Low deprivation | 8951 (26.7%) | 16721 (29.1%) | 53261 (34.6%) | 49819 (35.9%) | 37168 (33.7%) |
| Moderate deprivation | 10734 (32.0%) | 18562 (32.3%) | 52317 (34.0%) | 47243 (34.1%) | 36267 (32.8%) |
| High deprivation | 13837 (41.2%) | 22061 (38.4%) | 48041 (31.2%) | 41425 (29.9%) | 36871 (33.4%) |
| Unknown | 47 (0.1%) | 89 (0.2%) | 178 (0.1%) | 161 (0.1%) | 135 (0.1%) |
| Educational level |  |  |  |  |  |
| Without college/university degree | 18104 (53.9%) | 40318 (70.2%) | 100637 (65.4%) | 92968 (67.1%) | 75611 (68.5%) |
| With college/university degree | 15055 (44.8%) | 16363 (28.5%) | 52016 (33.8%) | 44513 (32.1%) | 33549 (30.4%) |
| Unknown | 410 (1.2%) | 752 (1.3%) | 1144 (0.7%) | 1167 (0.8%) | 1281 (1.2%) |
| Smoking status |  |  |  |  |  |
| Never | 19810 (59.0%) | 33242 (57.9%) | 84230 (54.8%) | 75212 (54.2%) | 57105 (51.7%) |
| Past | 10905 (32.5%) | 18035 (31.4%) | 54149 (35.2%) | 49053 (35.4%) | 38799 (35.1%) |
| Current | 2723 (8.1%) | 5941 (10.3%) | 14976 (9.7%) | 13973 (10.1%) | 14121 (12.8%) |
| Unknown | 131 (0.4%) | 215 (0.4%) | 442 (0.3%) | 410 (0.3%) | 416 (0.4%) |
| Physical activity |  |  |  |  |  |
| Low level | 4304 (12.8%) | 8484 (14.8%) | 23659 (15.4%) | 21719 (15.7%) | 17169 (15.5%) |
| Moderate level | 10886 (32.4%) | 17989 (31.3%) | 51569 (33.5%) | 46831 (33.8%) | 35313 (32.0%) |
| High level | 12366 (36.8%) | 18715 (32.6%) | 49677 (32.3%) | 44022 (31.8%) | 36004 (32.6%) |
| Unknown | 6013 (17.9%) | 12245 (21.3%) | 28892 (18.8%) | 26076 (18.8%) | 21955 (19.9%) |
| Body mass index (BMI) |  |  |  |  |  |
| Normal/underweight (<25 Kg/m2) | 16440 (49.0%) | 20794 (36.2%) | 52206 (33.9%) | 43128 (31.1%) | 30338 (27.5%) |
| Overweight (25-29.9 Kg/m2) | 11674 (34.8%) | 23171 (40.3%) | 65796 (42.8%) | 60294 (43.5%) | 47877 (43.4%) |
| Obese (≥30 Kg/m2) | 5178 (15.4%) | 13086 (22.8%) | 35192 (22.9%) | 34667 (25.0%) | 31579 (28.6%) |
| Unknown | 277 (0.8%) | 382 (0.7%) | 603 (0.4%) | 559 (0.4%) | 647 (0.6%) |
| Sleep duration |  |  |  |  |  |
| <7 hours/day | 8747 (26.1%) | 15546 (27.1%) | 37424 (24.3%) | 32232 (23.2%) | 27038 (24.5%) |
| 7-8 hours/day | 22212 (66.2%) | 36961 (64.4%) | 105031 (68.3%) | 95417 (68.8%) | 73231 (66.3%) |
| >8 hours/day | 2384 (7.1%) | 4528 (7.9%) | 10646 (6.9%) | 10404 (7.5%) | 9602 (8.7%) |
| Unknown | 226 (0.7%) | 398 (0.7%) | 696 (0.5%) | 595 (0.4%) | 570 (0.5%) |
| With stroke history | 384 (1.1%) | 920 (1.6%) | 2105 (1.4%) | 2009 (1.4%) | 1979 (1.8%) |
| With family history of dementia | 3771 (11.2%) | 6201 (10.8%) | 135520 (88.1%) | 16383 (11.8%) | 13096 (11.9%) |
| *APOE* ε4 carrying status |  |  |  |  |  |
| Non-carriers | 19975 (59.5%) | 33576 (58.5%) | 90340 (58.7%) | 81709 (58.9%) | 64782 (58.7%) |
| Carriers | 8199 (24.4%) | 13633 (23.7%) | 36476 (23.7%) | 32335 (23.3%) | 25230 (22.8%) |
| Missing | 5395 (16.1%) | 10224 (17.8%) | 26981 (17.5%) | 24604 (17.7%) | 20429 (18.5%) |
| Total meat |  |  |  |  |  |
| Never | 20473 (61.0%) | 0 (0.0%) | 0 (0.0%) | 0 (0.0%) | 0 (0.0%) |
| ≤3 times/week | 9109 (27.1%) | 23310 (40.6%) | 40945 (26.6%) | 3861 (2.8%) | 36 (0.0%) |
| 3-5 times/week | 2611 (7.8%) | 17337 (30.2%) | 20870 (13.6%) | 42201 (30.4%) | 7046 (6.4%) |
| ≥5 times/week | 1087 (3.2%) | 9826 (17.1%) | 64548 (42.0%) | 62867 (45.3%) | 24242 (22.0%) |
| ≥7 times/week | 289 (0.9%) | 6960 (12.1%) | 27434 (17.8%) | 29719 (21.4%) | 79117 (71.6%) |
| Vegetables/Fruits |  |  |  |  |  |
| <2 serving/day | 1383 (4.1%) | 4276 (7.4%) | 8865 (5.8%) | 7414 (5.3%) | 7022 (6.4%) |
| <4 servings/day | 5930 (17.7%) | 14093 (24.5%) | 41202 (26.8%) | 40224 (29.0%) | 31901 (28.9%) |
| 4-6 servings/day | 12000 (35.7%) | 20579 (35.8%) | 60431 (39.3%) | 55699 (40.2%) | 42144 (38.2%) |
| >6 servings/day | 13332 (39.7%) | 16726 (29.1%) | 39715 (25.8%) | 32347 (23.3%) | 26367 (23.9%) |
| Unknown | 924 (2.8%) | 1759 (3.1%) | 3584 (2.3%) | 2964 (2.1%) | 3007 (2.7%) |
| Total fish |  |  |  |  |  |
| ≤1 times/week | 14683 (43.7%) | 16265 (28.3%) | 37577 (24.4%) | 30233 (21.8%) | 28222 (25.6%) |
| 1-2 times/week | 3067 (9.1%) | 10412 (18.1%) | 35170 (22.9%) | 33537 (24.2%) | 25033 (22.7%) |
| ≥2 times/week | 6343 (18.9%) | 16183 (28.2%) | 47173 (30.7%) | 46068 (33.2%) | 34433 (31.2%) |
| ≥4 times/week | 9278 (27.6%) | 14021 (24.4%) | 33068 (21.5%) | 28120 (20.3%) | 21844 (19.8%) |
| Unknown | 198 (0.6%) | 552 (1.0%) | 809 (0.5%) | 690 (0.5%) | 909 (0.8%) |
| Alcohol |  |  |  |  |  |
| Less than once a week | 14567 (43.4%) | 24467 (42.6%) | 45648 (29.7%) | 35940 (25.9%) | 29953 (27.1%) |
| Once or twice a week | 7338 (21.9%) | 14693 (25.6%) | 41645 (27.1%) | 36803 (26.5%) | 27050 (24.5%) |
| Three or four times a week | 6449 (19.2%) | 10281 (17.9%) | 36515 (23.7%) | 34792 (25.1%) | 26464 (24.0%) |
| Daily or almost daily | 5179 (15.4%) | 7946 (13.8%) | 29898 (19.4%) | 31039 (22.4%) | 26882 (24.3%) |
| Unknown | 36 (0.1%) | 46 (0.1%) | 91 (0.1%) | 74 (0.1%) | 92 (0.1%) |
| Tea/Coffee |  |  |  |  |  |
| ≤3 cups/day | 10451 (31.1%) | 15556 (27.1%) | 32947 (21.4%) | 27303 (19.7%) | 22579 (20.4%) |
| ≤5 cups/day | 10058 (30.0%) | 18254 (31.8%) | 51484 (33.5%) | 47067 (33.9%) | 35102 (31.8%) |
| ≤7 cups/day | 7542 (22.5%) | 13900 (24.2%) | 42064 (27.4%) | 39137 (28.2%) | 30017 (27.2%) |
| >7 cups/day | 5368 (16.0%) | 9457 (16.5%) | 26947 (17.5%) | 24866 (17.9%) | 22349 (20.2%) |
| Unknown | 150 (0.4%) | 266 (0.5%) | 355 (0.2%) | 275 (0.2%) | 394 (0.4%) |
| Energy intakes (KJ/day) | 8566 (2459) | 8469 (2333) | 8791 (2283) | 8907 (2205) | 9151 (2394) |
| Protein intakes (g/day) | 70.5 (21.2) | 78.8 (22.1) | 81.7 (21) | 84.5 (20.8) | 87.5 (22.6) |
| Fat intakes (g/day) | 75 (27.5) | 74.1 (26.4) | 77.6 (26) | 79.3 (26) | 81.6 (27.7) |
| Saturated fat intakes (g/day) | 28.1 (12.6) | 28.2 (11.7) | 29.8 (11.4) | 30.6 (11.3) | 31.8 (12.2) |
| Carbohydrate intakes (g/day) | 265.9 (90.3) | 254 (82.7) | 253.4 (76.5) | 252.5 (73.5) | 253.6 (79.3) |
| Iron intakes (mg/day) | 14.1 (4.3) | 13.2 (3.9) | 13.6 (3.8) | 13.8 (3.7) | 14 (3.9) |

## **Supplementary Table 5 Baseline characteristics of participants across categories of total meat intakes in UK Biobank cohort study**

|  |  |  | **Total meat** | (n=493,888) |  |
| --- | --- | --- | --- | --- | --- |
|  | 0 times/wk | 0.1-3.0 times/wk | 3.1-4.9 times/wk | 5.0-6.9 times/wk | ≥7.0 times/wk |
| Participants (%) | 20473 (4%) | 77261 (16%) | 90065 (18%) | 162570 (33%) | 143519 (29%) |
| Age at baseline (years) | 53.8 (8.0) | 57.1 (7.9) | 57.3 (7.9) | 56.7 (8.0) | 55.9 (8.2) |
| Gender |  |  |  |  |  |
| Men | 6213 (30.3%) | 26124 (33.8%) | 36297 (40.3%) | 73034 (44.9%) | 83023 (57.8%) |
| Women | 14260 (69.7%) | 51137 (66.2%) | 53768 (59.7%) | 89536 (55.1%) | 60496 (42.2%) |
| Ethnicity |  |  |  |  |  |
| White | 17804 (87.0%) | 71872 (93.0%) | 85921 (95.4%) | 156026 (96.0%) | 135212 (94.2%) |
| Asian | 1957 (9.6%) | 2533 (3.3%) | 1497 (1.7%) | 2099 (1.3%) | 2651 (1.8%) |
| Black | 225 (1.1%) | 1195 (1.5%) | 1170 (1.3%) | 2046 (1.3%) | 2818 (2.0%) |
| Mixed | 174 (0.8%) | 520 (0.7%) | 488 (0.5%) | 857 (0.5%) | 912 (0.6%) |
| Others/unknown | 313 (1.5%) | 1141 (1.5%) | 989 (1.1%) | 1542 (0.9%) | 1926 (1.3%) |
| Region |  |  |  |  |  |
| England | 18529 (90.5%) | 68784 (89.0%) | 79888 (88.7%) | 144459 (88.9%) | 126518 (88.2%) |
| Wales | 816 (4.0%) | 3221 (4.2%) | 3890 (4.3%) | 7067 (4.3%) | 5511 (3.8%) |
| Scotland | 1128 (5.5%) | 5256 (6.8%) | 6287 (7.0%) | 11044 (6.8%) | 11490 (8.0%) |
| Townsend deprivation index |  |  |  |  |  |
| Low deprivation | 5463 (26.7%) | 23531 (30.5%) | 31533 (35.0%) | 57671 (35.5%) | 47722 (33.3%) |
| Moderate deprivation | 6647 (32.5%) | 25654 (33.2%) | 30331 (33.7%) | 55141 (33.9%) | 47350 (33.0%) |
| High deprivation | 8336 (40.7%) | 27985 (36.2%) | 28096 (31.2%) | 49558 (30.5%) | 48260 (33.6%) |
| Unknown | 27 (0.1%) | 91 (0.1%) | 105 (0.1%) | 200 (0.1%) | 187 (0.1%) |
| Educational level |  |  |  |  |  |
| Without college/university degree | 9633 (47.1%) | 48094 (62.2%) | 60315 (67.0%) | 111382 (68.5%) | 98214 (68.4%) |
| With college/university degree | 10622 (51.9%) | 28312 (36.6%) | 28795 (32.0%) | 49883 (30.7%) | 43884 (30.6%) |
| Unknown | 218 (1.1%) | 855 (1.1%) | 955 (1.1%) | 1305 (0.8%) | 1421 (1.0%) |
| Smoking status |  |  |  |  |  |
| Never | 12240 (59.8%) | 43313 (56.1%) | 49895 (55.4%) | 89228 (54.9%) | 74923 (52.2%) |
| Past | 6706 (32.8%) | 26113 (33.8%) | 31008 (34.4%) | 56932 (35.0%) | 50182 (35.0%) |
| Current | 1447 (7.1%) | 7550 (9.8%) | 8836 (9.8%) | 15933 (9.8%) | 17968 (12.5%) |
| Unknown | 80 (0.4%) | 285 (0.4%) | 326 (0.4%) | 477 (0.3%) | 446 (0.3%) |
| Physical activity |  |  |  |  |  |
| Low level | 2615 (12.8%) | 11063 (14.3%) | 13650 (15.2%) | 25159 (15.5%) | 22848 (15.9%) |
| Moderate level | 6809 (33.3%) | 25502 (33.0%) | 29913 (33.2%) | 54014 (33.2%) | 46350 (32.3%) |
| High level | 7633 (37.3%) | 25482 (33.0%) | 28706 (31.9%) | 52242 (32.1%) | 46721 (32.6%) |
| Unknown | 3416 (16.7%) | 15214 (19.7%) | 17796 (19.8%) | 31155 (19.2%) | 27600 (19.2%) |
| Body mass index (BMI) |  |  |  |  |  |
| Normal/underweight (<25 Kg/m2) | 10609 (51.8%) | 32136 (41.6%) | 31555 (35.0%) | 50771 (31.2%) | 37835 (26.4%) |
| Overweight (25-29.9 Kg/m2) | 6946 (33.9%) | 30439 (39.4%) | 38361 (42.6%) | 70506 (43.4%) | 62560 (43.6%) |
| Obese (≥30 Kg/m2) | 2751 (13.4%) | 14234 (18.4%) | 19737 (21.9%) | 40578 (25.0%) | 42402 (29.5%) |
| Unknown | 167 (0.8%) | 452 (0.6%) | 412 (0.5%) | 715 (0.4%) | 722 (0.5%) |
| Sleep duration |  |  |  |  |  |
| <7 hours/day | 5161 (25.2%) | 19466 (25.2%) | 21174 (23.5%) | 38792 (23.9%) | 36394 (25.4%) |
| 7-8 hours/day | 13923 (68.0%) | 51233 (66.3%) | 61594 (68.4%) | 111073 (68.3%) | 95029 (66.2%) |
| >8 hours/day | 1284 (6.3%) | 6012 (7.8%) | 6876 (7.6%) | 12008 (7.4%) | 11384 (7.9%) |
| Unknown | 105 (0.5%) | 550 (0.7%) | 421 (0.5%) | 697 (0.4%) | 712 (0.5%) |
| With stroke history | 178 (0.9%) | 1129 (1.5%) | 1356 (1.5%) | 2317 (1.4%) | 2417 (1.7%) |
| With family history of dementia | 2251 (11.0%) | 9207 (11.9%) | 10747 (11.9%) | 19084 (11.7%) | 16439 (11.5%) |
| *APOE* ε4 carrying status |  |  |  |  |  |
| Non-carriers | 12293 (60.0%) | 45649 (59.1%) | 53255 (59.1%) | 95343 (58.6%) | 83842 (58.4%) |
| Carriers | 5018 (24.5%) | 18437 (23.9%) | 20944 (23.3%) | 38129 (23.5%) | 33345 (23.2%) |
| Missing | 3162 (15.4%) | 13175 (17.1%) | 15866 (17.6%) | 29098 (17.9%) | 26332 (18.3%) |
| Total meat |  |  |  |  |  |
| Never | 20473 (100%) | 0 (0.0%) | 0 (0.0%) | 0 (0.0%) | 0 (0.0%) |
| ≤3 times/week | 0 (0.0%) | 77261 (100%) | 0 (0.0%) | 0 (0.0%) | 0 (0.0%) |
| 3-5 times/week | 0 (0.0%) | 0 (0.0%) | 90065 (100%) | 0 (0.0%) | 0 (0.0%) |
| ≥5 times/week | 0 (0.0%) | 0 (0.0%) | 0 (0.0%) | 162570 (100%) | 0 (0.0%) |
| ≥7 times/week | 0 (0.0%) | 0 (0.0%) | 0 (0.0%) | 0 (0.0%) | 143519 (100%) |
| Vegetables/Fruits |  |  |  |  |  |
| <2 serving/day | 512 (2.5%) | 4173 (5.4%) | 4763 (5.3%) | 8769 (5.4%) | 10743 (7.5%) |
| <4 servings/day | 3392 (16.6%) | 17244 (22.3%) | 24354 (27.0%) | 44694 (27.5%) | 43666 (30.4%) |
| 4-6 servings/day | 7485 (36.6%) | 29257 (37.9%) | 35502 (39.4%) | 64908 (39.9%) | 53701 (37.4%) |
| >6 servings/day | 8586 (41.9%) | 24349 (31.5%) | 23220 (25.8%) | 40581 (25.0%) | 31751 (22.1%) |
| Unknown | 498 (2.4%) | 2238 (2.9%) | 2226 (2.5%) | 3618 (2.2%) | 3658 (2.5%) |
| Total fish |  |  |  |  |  |
| ≤1 times/week | 11325 (55.3%) | 20475 (26.5%) | 20968 (23.3%) | 37181 (22.9%) | 37031 (25.8%) |
| 1-2 times/week | 1413 (6.9%) | 15411 (19.9%) | 20724 (23.0%) | 37238 (22.9%) | 32433 (22.6%) |
| ≥2 times/week | 2989 (14.6%) | 22160 (28.7%) | 29221 (32.4%) | 52294 (32.2%) | 43536 (30.3%) |
| ≥4 times/week | 4673 (22.8%) | 18606 (24.1%) | 18616 (20.7%) | 34954 (21.5%) | 29482 (20.5%) |
| Unknown | 73 (0.4%) | 609 (0.8%) | 536 (0.6%) | 903 (0.6%) | 1037 (0.7%) |
| Alcohol |  |  |  |  |  |
| Less than once a week | 8534 (41.7%) | 28750 (37.2%) | 28290 (31.4%) | 46693 (28.7%) | 38308 (26.7%) |
| Once or twice a week | 4332 (21.2%) | 19609 (25.4%) | 23924 (26.6%) | 43736 (26.9%) | 35928 (25.0%) |
| Three or four times a week | 4224 (20.6%) | 15711 (20.3%) | 20030 (22.2%) | 39165 (24.1%) | 35371 (24.6%) |
| Daily or almost daily | 3360 (16.4%) | 13124 (17.0%) | 17768 (19.7%) | 32889 (20.2%) | 33803 (23.6%) |
| Unknown | 23 (0.1%) | 67 (0.1%) | 53 (0.1%) | 87 (0.1%) | 109 (0.1%) |
| Tea/Coffee |  |  |  |  |  |
| ≤3 cups/day | 6464 (31.6%) | 20176 (26.1%) | 19863 (22.1%) | 33512 (20.6%) | 28821 (20.1%) |
| ≤5 cups/day | 6081 (29.7%) | 25440 (32.9%) | 30735 (34.1%) | 54393 (33.5%) | 45316 (31.6%) |
| ≤7 cups/day | 4603 (22.5%) | 18972 (24.6%) | 24176 (26.8%) | 45508 (28.0%) | 39401 (27.5%) |
| >7 cups/day | 3231 (15.8%) | 12395 (16.0%) | 15037 (16.7%) | 28812 (17.7%) | 29512 (20.6%) |
| Unknown | 94 (0.5%) | 278 (0.4%) | 254 (0.3%) | 345 (0.2%) | 469 (0.3%) |
| Energy intakes (KJ/day) | 8670 (2519) | 8409 (2185) | 8606 (2193) | 8811 (2212) | 9341 (2443) |
| Protein intakes (g/day) | 68.3 (20.2) | 76.4 (20.6) | 80.8 (20.3) | 83.7 (20.6) | 88.7 (23) |
| Fat intakes (g/day) | 76 (27.7) | 73.5 (25.4) | 75.9 (25.1) | 77.9 (25.7) | 83.8 (28.2) |
| Saturated fat intakes (g/day) | 28.4 (12.8) | 28.2 (11.5) | 29.3 (11.1) | 30 (11.2) | 32.4 (12.3) |
| Carbohydrate intakes (g/day) | 269.7 (91) | 250.3 (80) | 248.6 (74.9) | 252.1 (74.4) | 259.8 (80) |
| Iron intakes (mg/day) | 14.4 (4.3) | 13.5 (3.9) | 13.6 (3.7) | 13.7 (3.7) | 14.1 (3.9) |

## **Supplementary Table 6 Risks of Alzheimer’s disease and vascular dementia under different meat types among APOE Ɛ4 non-carriers (n=289 589) and carriers (n=115 537) respectively**

|  |  | **Unadjusted models**  **(n = 405 126)** | | | |  | **Minimally-adjusted**  **Models ^1^ (n = 405 126)** | | | |  | **Fully-adjusted models ^2^**  **(n = 405 126)** | | | |
| --- | --- | --- | --- | --- | --- | --- | --- | --- | --- | --- | --- | --- | --- | --- | --- |
|  |  | HR | LCI | UCI | *P* |  | HR | LCI | UCI | *P* |  | HR | LCI | UCI | *P* |
| **Risk of Alzheimer’s disease** |  |  |  |  |  |  |  |  |  |  |  |  |  |  |  |
| *APOE Ɛ4 carriers vs. non-carriers* |  | 5.74 | 3.24 | 10.2 | <0.001 |  | 6.30 | 3.24 | 12.2 | <0.001 |  | 5.87 | 3.06 | 11.2 | <0.001 |
| **Processed meat (**25 g per day**)** |  |  |  |  |  |  |  |  |  |  |  |  |  |  |  |
| *Stratified analysis* |  |  |  |  |  |  |  |  |  |  |  |  |  |  |  |
| *APOE* Ɛ4 non-carriers |  | 1.41 | 0.94 | 2.12 | 0.100 |  | 1.28 | 0.82 | 2.00 | 0.270 |  | 1.56 | 0.96 | 2.52 | 0.071 |
| *APOE* Ɛ4 carriers |  | 0.97 | 0.72 | 1.31 | 0.828 |  | 0.92 | 0.66 | 1.28 | 0.624 |  | 1.37 | 0.96 | 1.93 | 0.079 |
| *P for interaction with APOE Ɛ4 allele* |  |  |  |  | 0.148 |  |  |  |  | 0.137 |  |  |  |  | 0.342 |
| **Unprocessed poultry (**25 g per day**)** |  |  |  |  |  |  |  |  |  |  |  |  |  |  |  |
| *Stratified analysis* |  |  |  |  |  |  |  |  |  |  |  |  |  |  |  |
| *APOE* Ɛ4 non-carriers |  | 0.90 | 0.71 | 1.14 | 0.377 |  | 1.02 | 0.77 | 1.35 | 0.884 |  | 1.02 | 0.77 | 1.35 | 0.883 |
| *APOE* Ɛ4 carriers |  | 0.84 | 0.70 | 1.01 | 0.069 |  | 0.91 | 0.73 | 1.13 | 0.391 |  | 0.98 | 0.79 | 1.23 | 0.886 |
| *P for interaction with APOE Ɛ4 allele* |  |  |  |  | 0.681 |  |  |  |  | 0.601 |  |  |  |  | 0.975 |
| **Unprocessed red meat (**50 g per day**)** |  |  |  |  |  |  |  |  |  |  |  |  |  |  |  |
| *Stratified analysis* |  |  |  |  |  |  |  |  |  |  |  |  |  |  |  |
| *APOE* Ɛ4 non-carriers |  | 1.20 | 0.77 | 1.88 | 0.422 |  | 0.88 | 0.56 | 1.38 | 0.579 |  | 0.87 | 0.52 | 1.44 | 0.577 |
| *APOE* Ɛ4 carriers |  | 0.82 | 0.60 | 1.13 | 0.221 |  | 0.58 | 0.42 | 0.81 | 0.001 |  | 0.61 | 0.42 | 0.87 | 0.007 |
| *P for interaction with APOE Ɛ4 allele* |  |  |  |  | 0.174 |  |  |  |  | 0.174 |  |  |  |  | 0.392 |
| **Total meat (**50 g per day**)** |  |  |  |  |  |  |  |  |  |  |  |  |  |  |  |
| *Stratified analysis* |  |  |  |  |  |  |  |  |  |  |  |  |  |  |  |
| *APOE* Ɛ4 non-carriers |  | 1.19 | 0.93 | 1.52 | 0.179 |  | 1.13 | 0.85 | 1.49 | 0.412 |  | 1.23 | 0.93 | 1.63 | 0.147 |
| *APOE* Ɛ4 carriers |  | 1.04 | 0.87 | 1.24 | 0.672 |  | 0.95 | 0.77 | 1.18 | 0.646 |  | 1.05 | 0.85 | 1.30 | 0.649 |
| *P for interaction with APOE Ɛ4 allele* |  |  |  |  | 0.395 |  |  |  |  | 0.335 |  |  |  |  | 0.329 |
| **Risk of Vascular dementia** |  |  |  |  |  |  |  |  |  |  |  |  |  |  |  |
| *APOE Ɛ4 carriers vs. non-carriers* |  | 4.85 | 2.48 | 9.46 | <0.001 |  | 5.04 | 2.57 | 9.92 | <0.001 |  | 4.65 | 1.88 | 11.5 | 0.001 |
| **Processed meat (**25 g per day**)** |  |  |  |  |  |  |  |  |  |  |  |  |  |  |  |
| *Stratified analysis* |  |  |  |  |  |  |  |  |  |  |  |  |  |  |  |
| *APOE* Ɛ4 non-carriers |  | 1.97 | 1.12 | 3.45 | 0.018 |  | 1.52 | 0.85 | 2.74 | 0.161 |  | 1.57 | 0.85 | 2.89 | 0.149 |
| *APOE* Ɛ4 carriers |  | 1.11 | 0.69 | 1.80 | 0.673 |  | 0.92 | 0.54 | 1.54 | 0.738 |  | 1.10 | 0.62 | 1.95 | 0.749 |
| *P for interaction with APOE Ɛ4 allele* |  |  |  |  | 0.128 |  |  |  |  | 0.112 |  |  |  |  | 0.198 |
| **Unprocessed poultry (**25 g per day**)** |  |  |  |  |  |  |  |  |  |  |  |  |  |  |  |
| *Stratified analysis* |  |  |  |  |  |  |  |  |  |  |  |  |  |  |  |
| *APOE* Ɛ4 non-carriers |  | 0.96 | 0.65 | 1.40 | 0.820 |  | 1.10 | 0.71 | 1.72 | 0.665 |  | 1.11 | 0.72 | 1.73 | 0.632 |
| *APOE* Ɛ4 carriers |  | 1.00 | 0.75 | 1.33 | 0.990 |  | 1.17 | 0.83 | 1.66 | 0.362 |  | 1.20 | 0.83 | 1.73 | 0.325 |
| *P for interaction with APOE Ɛ4 allele* |  |  |  |  | 0.863 |  |  |  |  | 0.968 |  |  |  |  | 0.617 |
| **Unprocessed red meat (**50 g per day**)** |  |  |  |  |  |  |  |  |  |  |  |  |  |  |  |
| *Stratified analysis* |  |  |  |  |  |  |  |  |  |  |  |  |  |  |  |
| *APOE* Ɛ4 non-carriers |  | 1.42 | 0.76 | 2.68 | 0.275 |  | 0.99 | 0.53 | 1.84 | 0.971 |  | 0.88 | 0.45 | 1.73 | 0.706 |
| *APOE* Ɛ4 carriers |  | 0.96 | 0.57 | 1.62 | 0.876 |  | 0.66 | 0.39 | 1.11 | 0.118 |  | 0.64 | 0.36 | 1.14 | 0.129 |
| *P for interaction with APOE Ɛ4 allele* |  |  |  |  | 0.346 |  |  |  |  | 0.305 |  |  |  |  | 0.563 |
| **Total meat (**50 g per day**)** |  |  |  |  |  |  |  |  |  |  |  |  |  |  |  |
| *Stratified analysis* |  |  |  |  |  |  |  |  |  |  |  |  |  |  |  |
| *APOE* Ɛ4 non-carriers |  | 1.33 | 0.91 | 1.93 | 0.138 |  | 1.19 | 0.80 | 1.76 | 0.393 |  | 1.23 | 0.83 | 1.83 | 0.298 |
| *APOE* Ɛ4 carriers |  | 1.08 | 0.85 | 1.39 | 0.526 |  | 0.96 | 0.72 | 1.29 | 0.791 |  | 0.98 | 0.73 | 1.32 | 0.906 |
| *P for interaction with APOE Ɛ4 allele* |  |  |  |  | 0.374 |  |  |  |  | 0.289 |  |  |  |  | 0.263 |

^1^ Minimally-adjusted models: Cox proportional-hazards regression adjusted for age, gender, ethnicity, education, socioeconomic status. **^2^** Fully-adjusted models: Cox proportional-hazards regression additionally adjusted for region, smoking status, physical activity, body mass index, sleep duration, stroke history, family history of dementia, genetic kinship to other participants, dietary covariates including vegetables and fruits, total fish, tea and coffee, alcohol drinking; processed meat, unprocessed poultry, and unprocessed red meat were also mutually adjusted for. Mean daily intakes per increment calculated from the multiple 24-h dietary assessments were used as continuous variables in Cox models. Abbreviations: *APOE,* apolipoprotein E; HR, hazard ratio; LCI, lower confidence interval (95%); UCI, upper confidence interval (95%).

## **Supplementary Table 7 Risks of all-cause dementia, Alzheimer’s disease and vascular dementia under different meat types among APOE Ɛ4 non-carriers (n=289 441) and carriers (n=115 421) respectively in sensitivity analysis excluding dementia cases within first 3 years of follow-up**

|  |  | **Unadjusted models**  **(n = 404 862)** | | | |  | **Minimally-adjusted**  **Models ^1^ (n = 404 862)** | | | |  | **Fully-adjusted models ^2^**  **(n = 404 862)** | | | |
| --- | --- | --- | --- | --- | --- | --- | --- | --- | --- | --- | --- | --- | --- | --- | --- |
|  |  | HR | LCI | UCI | *P* |  | HR | LCI | UCI | *P* |  | HR | LCI | UCI | *P* |
| **Risk of All-cause dementia** |  |  |  |  |  |  |  |  |  |  |  |  |  |  |  |
| *APOE Ɛ4 carriers vs. non-carriers* |  | 3.29 | 2.32 | 4.65 | <0.001 |  | 3.55 | 2.40 | 5.24 | <0.001 |  | 3.46 | 2.35 | 5.08 | <0.001 |
| **Processed meat (**25 g per day**)** |  |  |  |  |  |  |  |  |  |  |  |  |  |  |  |
| *Stratified analysis* |  |  |  |  |  |  |  |  |  |  |  |  |  |  |  |
| *APOE* Ɛ4 non-carriers |  | 1.60 | 1.28 | 2.00 | <0.001 |  | 1.34 | 1.05 | 1.70 | 0.018 |  | 1.45 | 1.13 | 1.86 | 0.004 |
| *APOE* Ɛ4 carriers |  | 1.18 | 0.95 | 1.45 | 0.128 |  | 1.11 | 0.89 | 1.40 | 0.352 |  | 1.46 | 1.15 | 1.85 | 0.002 |
| *P for interaction with APOE Ɛ4 allele* |  |  |  |  | 0.049 |  |  |  |  | 0.048 |  |  |  |  | 0.176 |
| **Unprocessed poultry (**25 g per day**)** |  |  |  |  |  |  |  |  |  |  |  |  |  |  |  |
| *Stratified analysis* |  |  |  |  |  |  |  |  |  |  |  |  |  |  |  |
| *APOE* Ɛ4 non-carriers |  | 0.85 | 0.74 | 0.98 | 0.026 |  | 0.93 | 0.79 | 1.10 | 0.392 |  | 0.96 | 0.81 | 1.14 | 0.670 |
| *APOE* Ɛ4 carriers |  | 0.84 | 0.73 | 0.95 | 0.007 |  | 0.91 | 0.78 | 1.06 | 0.226 |  | 0.95 | 0.82 | 1.11 | 0.546 |
| *P for interaction with APOE Ɛ4 allele* |  |  |  |  | 0.847 |  |  |  |  | 0.698 |  |  |  |  | 0.880 |
| **Unprocessed red meat (**50 g per day**)** |  |  |  |  |  |  |  |  |  |  |  |  |  |  |  |
| *Stratified analysis* |  |  |  |  |  |  |  |  |  |  |  |  |  |  |  |
| *APOE* Ɛ4 non-carriers |  | 1.24 | 0.96 | 1.58 | 0.095 |  | 0.89 | 0.69 | 1.14 | 0.336 |  | 0.87 | 0.65 | 1.15 | 0.311 |
| *APOE* Ɛ4 carriers |  | 0.93 | 0.74 | 1.18 | 0.558 |  | 0.68 | 0.54 | 0.86 | 0.001 |  | 0.68 | 0.52 | 0.88 | 0.003 |
| *P for interaction with APOE Ɛ4 allele* |  |  |  |  | 0.108 |  |  |  |  | 0.104 |  |  |  |  | 0.333 |
| **Total meat (**50 g per day**)** |  |  |  |  |  |  |  |  |  |  |  |  |  |  |  |
| *Stratified analysis* |  |  |  |  |  |  |  |  |  |  |  |  |  |  |  |
| *APOE* Ɛ4 non-carriers |  | 1.18 | 1.03 | 1.36 | 0.017 |  | 1.07 | 0.92 | 1.25 | 0.368 |  | 1.13 | 0.97 | 1.32 | 0.122 |
| *APOE* Ɛ4 carriers |  | 1.04 | 0.92 | 1.18 | 0.541 |  | 0.95 | 0.82 | 1.10 | 0.513 |  | 1.02 | 0.88 | 1.18 | 0.793 |
| *P for interaction with APOE Ɛ4 allele* |  |  |  |  | 0.170 |  |  |  |  | 0.123 |  |  |  |  | 0.109 |
| **Risk of Alzheimer’s disease** |  |  |  |  |  |  |  |  |  |  |  |  |  |  |  |
| *APOE Ɛ4 carriers vs. non-carriers* |  | 6.53 | 3.54 | 12.1 | <0.001 |  | 7.37 | 3.62 | 15.0 | <0.001 |  | 6.78 | 3.39 | 13.6 | <0.001 |
| **Processed meat (**25 g per day**)** |  |  |  |  |  |  |  |  |  |  |  |  |  |  |  |
| *Stratified analysis* |  |  |  |  |  |  |  |  |  |  |  |  |  |  |  |
| *APOE* Ɛ4 non-carriers |  | 1.58 | 1.04 | 2.42 | 0.034 |  | 1.48 | 0.94 | 2.35 | 0.093 |  | 1.75 | 1.05 | 2.90 | 0.031 |
| *APOE* Ɛ4 carriers |  | 1.04 | 0.76 | 1.42 | 0.798 |  | 1.01 | 0.72 | 1.42 | 0.959 |  | 1.49 | 1.05 | 2.12 | 0.025 |
| *P for interaction with APOE Ɛ4 allele* |  |  |  |  | 0.120 |  |  |  |  | 0.110 |  |  |  |  | 0.305 |
| **Unprocessed poultry (**25 g per day**)** |  |  |  |  |  |  |  |  |  |  |  |  |  |  |  |
| *Stratified analysis* |  |  |  |  |  |  |  |  |  |  |  |  |  |  |  |
| *APOE* Ɛ4 non-carriers |  | 0.97 | 0.75 | 1.25 | 0.789 |  | 1.11 | 0.83 | 1.50 | 0.484 |  | 1.10 | 0.82 | 1.48 | 0.541 |
| *APOE* Ɛ4 carriers |  | 0.85 | 0.70 | 1.03 | 0.102 |  | 0.91 | 0.72 | 1.16 | 0.458 |  | 0.98 | 0.78 | 1.24 | 0.864 |
| *P for interaction with APOE Ɛ4 allele* |  |  |  |  | 0.432 |  |  |  |  | 0.370 |  |  |  |  | 0.690 |
| **Unprocessed red meat (**50 g per day**)** |  |  |  |  |  |  |  |  |  |  |  |  |  |  |  |
| *Stratified analysis* |  |  |  |  |  |  |  |  |  |  |  |  |  |  |  |
| *APOE* Ɛ4 non-carriers |  | 1.24 | 0.78 | 1.97 | 0.370 |  | 0.91 | 0.57 | 1.45 | 0.701 |  | 0.82 | 0.48 | 1.40 | 0.459 |
| *APOE* Ɛ4 carriers |  | 0.85 | 0.61 | 1.18 | 0.329 |  | 0.60 | 0.43 | 0.85 | 0.004 |  | 0.61 | 0.42 | 0.90 | 0.011 |
| *P for interaction with APOE Ɛ4 allele* |  |  |  |  | 0.196 |  |  |  |  | 0.193 |  |  |  |  | 0.508 |
| **Total meat (**50 g per day**)** |  |  |  |  |  |  |  |  |  |  |  |  |  |  |  |
| *Stratified analysis* |  |  |  |  |  |  |  |  |  |  |  |  |  |  |  |
| *APOE* Ɛ4 non-carriers |  | 1.28 | 0.98 | 1.67 | 0.071 |  | 1.24 | 0.92 | 1.67 | 0.168 |  | 1.34 | 0.99 | 1.80 | 0.059 |
| *APOE* Ɛ4 carriers |  | 1.04 | 0.86 | 1.25 | 0.687 |  | 0.95 | 0.76 | 1.20 | 0.684 |  | 1.06 | 0.85 | 1.33 | 0.614 |
| *P for interaction with APOE Ɛ4 allele* |  |  |  |  | 0.213 |  |  |  |  | 0.174 |  |  |  |  | 0.174 |
| **Risk of Vascular dementia** |  |  |  |  |  |  |  |  |  |  |  |  |  |  |  |
| *APOE Ɛ4 carriers vs. non-carriers* |  | 3.95 | 1.59 | 9.83 | 0.003 |  | 4.42 | 1.60 | 12.2 | 0.004 |  | 4.34 | 1.62 | 11.6 | 0.004 |
| **Processed meat (**25 g per day**)** |  |  |  |  |  |  |  |  |  |  |  |  |  |  |  |
| *Stratified analysis* |  |  |  |  |  |  |  |  |  |  |  |  |  |  |  |
| *APOE* Ɛ4 non-carriers |  | 1.78 | 0.98 | 3.24 | 0.057 |  | 1.35 | 0.72 | 2.54 | 0.346 |  | 1.37 | 0.71 | 2.65 | 0.346 |
| *APOE* Ɛ4 carriers |  | 1.11 | 0.68 | 1.83 | 0.677 |  | 0.94 | 0.55 | 1.62 | 0.833 |  | 1.09 | 0.61 | 1.96 | 0.773 |
| *P for interaction with APOE Ɛ4 allele* |  |  |  |  | 0.231 |  |  |  |  | 0.220 |  |  |  |  | 0.367 |
| **Unprocessed poultry (**25 g per day**)** |  |  |  |  |  |  |  |  |  |  |  |  |  |  |  |
| *Stratified analysis* |  |  |  |  |  |  |  |  |  |  |  |  |  |  |  |
| *APOE* Ɛ4 non-carriers |  | 1.02 | 0.67 | 1.54 | 0.937 |  | 1.16 | 0.72 | 1.89 | 0.543 |  | 1.21 | 0.76 | 1.92 | 0.427 |
| *APOE* Ɛ4 carriers |  | 1.10 | 0.79 | 1.52 | 0.582 |  | 1.30 | 0.89 | 1.90 | 0.184 |  | 1.35 | 0.92 | 1.99 | 0.124 |
| *P for interaction with APOE Ɛ4 allele* |  |  |  |  | 0.784 |  |  |  |  | 0.876 |  |  |  |  | 0.590 |
| **Unprocessed red meat (**50 g per day**)** |  |  |  |  |  |  |  |  |  |  |  |  |  |  |  |
| *Stratified analysis* |  |  |  |  |  |  |  |  |  |  |  |  |  |  |  |
| *APOE* Ɛ4 non-carriers |  | 1.41 | 0.71 | 2.79 | 0.324 |  | 0.94 | 0.48 | 1.82 | 0.853 |  | 0.87 | 0.42 | 1.78 | 0.698 |
| *APOE* Ɛ4 carriers |  | 0.92 | 0.53 | 1.58 | 0.758 |  | 0.64 | 0.37 | 1.11 | 0.115 |  | 0.58 | 0.31 | 1.06 | 0.075 |
| *P for interaction with APOE Ɛ4 allele* |  |  |  |  | 0.335 |  |  |  |  | 0.311 |  |  |  |  | 0.465 |
| **Total meat (**50 g per day**)** |  |  |  |  |  |  |  |  |  |  |  |  |  |  |  |
| *Stratified analysis* |  |  |  |  |  |  |  |  |  |  |  |  |  |  |  |
| *APOE* Ɛ4 non-carriers |  | 1.30 | 0.87 | 1.94 | 0.200 |  | 1.13 | 0.74 | 1.73 | 0.561 |  | 1.18 | 0.78 | 1.81 | 0.435 |
| *APOE* Ɛ4 carriers |  | 1.09 | 0.84 | 1.43 | 0.515 |  | 0.98 | 0.72 | 1.34 | 0.892 |  | 0.98 | 0.72 | 1.34 | 0.915 |
| *P for interaction with APOE Ɛ4 allele* |  |  |  |  | 0.479 |  |  |  |  | 0.404 |  |  |  |  | 0.375 |

^1^Minimally-adjusted models: Cox proportional hazards regression adjusted for age, gender, ethnicity, education, socioeconomic status. **^2^**Fully-adjusted models: Cox proportional hazards regression additionally adjusted for region, smoking status, physical activity, body mass index, sleep duration, stroke history, family history of dementia, genetic kinship to other participants, dietary covariates including vegetables and fruits, total fish, tea and coffee, alcohol drinking; processed meat, unprocessed poultry, and unprocessed red meat were also mutually adjusted for. Mean daily intakes per increment calculated from the multiple 24-h dietary assessments were used as continuous variables in Cox models. Abbreviations: HR, hazard ratio; LCI, lower confidence interval (95%); UCI, upper confidence interval (95%); *APOE,* apolipoprotein E.

## **Supplementary Table 8 Risks of all-cause dementia, Alzheimer’s disease and vascular dementia under different meat types among APOE Ɛ4 non-carriers (n=225 130) and carriers (n=90 072) respectively in sensitivity analysis excluding participants with missing data of covariates**

|  |  | **Unadjusted models**  **(n = 315 202)** | | | |  | **Minimally-adjusted**  **Models ^1^ (n = 315 202)** | | | |  | **Fully-adjusted models ^2^**  **(n = 315 202)** | | | |
| --- | --- | --- | --- | --- | --- | --- | --- | --- | --- | --- | --- | --- | --- | --- | --- |
|  |  | HR | LCI | UCI | *P* |  | HR | LCI | UCI | *P* |  | HR | LCI | UCI | *P* |
| **Risk of All-cause dementia** |  |  |  |  |  |  |  |  |  |  |  |  |  |  |  |
| *APOE Ɛ4 carriers vs. non-carriers* |  | 3.25 | 2.16 | 4.89 | <0.001 |  | 3.47 | 2.20 | 5.48 | <0.001 |  | 3.34 | 2.13 | 5.24 | <0.001 |
| **Processed meat (**25 g per day**)** |  |  |  |  |  |  |  |  |  |  |  |  |  |  |  |
| *Stratified analysis* |  |  |  |  |  |  |  |  |  |  |  |  |  |  |  |
| *APOE* Ɛ4 non-carriers |  | 1.70 | 1.33 | 2.17 | <0.001 |  | 1.41 | 1.08 | 1.84 | 0.011 |  | 1.51 | 1.14 | 2.01 | 0.004 |
| *APOE* Ɛ4 carriers |  | 1.20 | 0.94 | 1.55 | 0.148 |  | 1.07 | 0.82 | 1.41 | 0.611 |  | 1.44 | 1.08 | 1.92 | 0.012 |
| *P for interaction with APOE Ɛ4 allele* |  |  |  |  | 0.056 |  |  |  |  | 0.048 |  |  |  |  | 0.205 |
| **Unprocessed poultry (**25 g per day**)** |  |  |  |  |  |  |  |  |  |  |  |  |  |  |  |
| *Stratified analysis* |  |  |  |  |  |  |  |  |  |  |  |  |  |  |  |
| *APOE* Ɛ4 non-carriers |  | 0.83 | 0.71 | 0.97 | 0.021 |  | 0.90 | 0.75 | 1.08 | 0.265 |  | 0.89 | 0.73 | 1.08 | 0.227 |
| *APOE* Ɛ4 carriers |  | 0.81 | 0.70 | 0.94 | 0.006 |  | 0.88 | 0.74 | 1.05 | 0.165 |  | 0.92 | 0.76 | 1.10 | 0.360 |
| *P for interaction with APOE Ɛ4 allele* |  |  |  |  | 0.816 |  |  |  |  | 0.763 |  |  |  |  | 0.674 |
| **Unprocessed red meat (**50 g per day**)** |  |  |  |  |  |  |  |  |  |  |  |  |  |  |  |
| *Stratified analysis* |  |  |  |  |  |  |  |  |  |  |  |  |  |  |  |
| *APOE* Ɛ4 non-carriers |  | 1.41 | 1.07 | 1.87 | 0.017 |  | 0.99 | 0.75 | 1.32 | 0.964 |  | 0.99 | 0.72 | 1.37 | 0.958 |
| *APOE* Ɛ4 carriers |  | 0.97 | 0.74 | 1.28 | 0.847 |  | 0.67 | 0.51 | 0.89 | 0.005 |  | 0.69 | 0.50 | 0.94 | 0.019 |
| *P for interaction with APOE Ɛ4 allele* |  |  |  |  | 0.065 |  |  |  |  | 0.063 |  |  |  |  | 0.228 |
| **Total meat (**50 g per day**)** |  |  |  |  |  |  |  |  |  |  |  |  |  |  |  |
| *Stratified analysis* |  |  |  |  |  |  |  |  |  |  |  |  |  |  |  |
| *APOE* Ɛ4 non-carriers |  | 1.25 | 1.06 | 1.47 | 0.008 |  | 1.13 | 0.94 | 1.35 | 0.187 |  | 1.18 | 0.98 | 1.42 | 0.075 |
| *APOE* Ɛ4 carriers |  | 1.07 | 0.93 | 1.24 | 0.344 |  | 0.97 | 0.82 | 1.15 | 0.738 |  | 1.04 | 0.88 | 1.23 | 0.635 |
| *P for interaction with APOE Ɛ4 allele* |  |  |  |  | 0.176 |  |  |  |  | 0.156 |  |  |  |  | 0.164 |
| **Risk of Alzheimer’s disease** |  |  |  |  |  |  |  |  |  |  |  |  |  |  |  |
| *APOE Ɛ4 carriers vs. non-carriers* |  | 6.89 | 3.34 | 14.2 | <0.001 |  | 7.69 | 3.31 | 17.9 | <0.001 |  | 7.05 | 3.10 | 16.0 | <0.001 |
| **Processed meat (**25 g per day**)** |  |  |  |  |  |  |  |  |  |  |  |  |  |  |  |
| *Stratified analysis* |  |  |  |  |  |  |  |  |  |  |  |  |  |  |  |
| *APOE* Ɛ4 non-carriers |  | 1.75 | 1.08 | 2.84 | 0.024 |  | 1.58 | 0.92 | 2.71 | 0.098 |  | 1.90 | 1.07 | 3.39 | 0.029 |
| *APOE* Ɛ4 carriers |  | 0.83 | 0.58 | 1.20 | 0.317 |  | 0.76 | 0.51 | 1.14 | 0.185 |  | 1.15 | 0.76 | 1.75 | 0.517 |
| *P for interaction with APOE Ɛ4 allele* |  |  |  |  | 0.017 |  |  |  |  | 0.016 |  |  |  |  | 0.086 |
| **Unprocessed poultry (**25 g per day**)** |  |  |  |  |  |  |  |  |  |  |  |  |  |  |  |
| *Stratified analysis* |  |  |  |  |  |  |  |  |  |  |  |  |  |  |  |
| *APOE* Ɛ4 non-carriers |  | 0.96 | 0.71 | 1.29 | 0.760 |  | 1.09 | 0.76 | 1.56 | 0.633 |  | 1.05 | 0.74 | 1.49 | 0.802 |
| *APOE* Ɛ4 carriers |  | 0.85 | 0.67 | 1.07 | 0.167 |  | 0.92 | 0.69 | 1.21 | 0.543 |  | 1.05 | 0.80 | 1.37 | 0.714 |
| *P for interaction with APOE Ɛ4 allele* |  |  |  |  | 0.541 |  |  |  |  | 0.519 |  |  |  |  | 0.872 |
| **Unprocessed red meat (**50 g per day**)** |  |  |  |  |  |  |  |  |  |  |  |  |  |  |  |
| *Stratified analysis* |  |  |  |  |  |  |  |  |  |  |  |  |  |  |  |
| *APOE* Ɛ4 non-carriers |  | 1.42 | 0.82 | 2.48 | 0.212 |  | 0.99 | 0.56 | 1.75 | 0.976 |  | 0.88 | 0.47 | 1.64 | 0.683 |
| *APOE* Ɛ4 carriers |  | 0.75 | 0.51 | 1.11 | 0.145 |  | 0.51 | 0.34 | 0.76 | 0.001 |  | 0.56 | 0.35 | 0.88 | 0.012 |
| *P for interaction with APOE Ɛ4 allele* |  |  |  |  | 0.063 |  |  |  |  | 0.064 |  |  |  |  | 0.317 |
| **Total meat (**50 g per day**)** |  |  |  |  |  |  |  |  |  |  |  |  |  |  |  |
| *Stratified analysis* |  |  |  |  |  |  |  |  |  |  |  |  |  |  |  |
| *APOE* Ɛ4 non-carriers |  | 1.29 | 0.94 | 1.79 | 0.120 |  | 1.22 | 0.84 | 1.77 | 0.306 |  | 1.33 | 0.92 | 1.93 | 0.132 |
| *APOE* Ɛ4 carriers |  | 1.00 | 0.81 | 1.22 | 0.969 |  | 0.90 | 0.70 | 1.16 | 0.419 |  | 1.00 | 0.78 | 1.28 | 0.997 |
| *P for interaction with APOE Ɛ4 allele* |  |  |  |  | 0.182 |  |  |  |  | 0.174 |  |  |  |  | 0.176 |
| **Risk of Vascular dementia** |  |  |  |  |  |  |  |  |  |  |  |  |  |  |  |
| *APOE Ɛ4 carriers vs. non-carriers* |  | 4.71 | 1.76 | 12.6 | 0.002 |  | 5.30 | 1.78 | 15.8 | 0.003 |  | 5.07 | 1.77 | 14.6 | 0.003 |
| **Processed meat (**25 g per day**)** |  |  |  |  |  |  |  |  |  |  |  |  |  |  |  |
| *Stratified analysis* |  |  |  |  |  |  |  |  |  |  |  |  |  |  |  |
| *APOE* Ɛ4 non-carriers |  | 1.84 | 0.96 | 3.51 | 0.066 |  | 1.32 | 0.66 | 2.64 | 0.432 |  | 1.23 | 0.59 | 2.56 | 0.576 |
| *APOE* Ɛ4 carriers |  | 0.96 | 0.53 | 1.72 | 0.881 |  | 0.74 | 0.40 | 1.40 | 0.357 |  | 0.86 | 0.43 | 1.72 | 0.667 |
| *P for interaction with APOE Ɛ4 allele* |  |  |  |  | 0.142 |  |  |  |  | 0.132 |  |  |  |  | 0.226 |
| **Unprocessed poultry (**25 g per day**)** |  |  |  |  |  |  |  |  |  |  |  |  |  |  |  |
| *Stratified analysis* |  |  |  |  |  |  |  |  |  |  |  |  |  |  |  |
| *APOE* Ɛ4 non-carriers |  | 0.95 | 0.62 | 1.46 | 0.816 |  | 1.08 | 0.65 | 1.79 | 0.762 |  | 1.07 | 0.65 | 1.77 | 0.797 |
| *APOE* Ɛ4 carriers |  | 0.84 | 0.61 | 1.18 | 0.318 |  | 0.96 | 0.63 | 1.45 | 0.828 |  | 0.90 | 0.57 | 1.43 | 0.669 |
| *P for interaction with APOE Ɛ4 allele* |  |  |  |  | 0.665 |  |  |  |  | 0.615 |  |  |  |  | 0.857 |
| **Unprocessed red meat (**50 g per day**)** |  |  |  |  |  |  |  |  |  |  |  |  |  |  |  |
| *Stratified analysis* |  |  |  |  |  |  |  |  |  |  |  |  |  |  |  |
| *APOE* Ɛ4 non-carriers |  | 2.00 | 0.95 | 4.24 | 0.070 |  | 1.31 | 0.64 | 2.70 | 0.464 |  | 1.31 | 0.60 | 2.83 | 0.499 |
| *APOE* Ɛ4 carriers |  | 1.26 | 0.65 | 2.44 | 0.487 |  | 0.82 | 0.42 | 1.61 | 0.559 |  | 1.02 | 0.50 | 2.11 | 0.952 |
| *P for interaction with APOE Ɛ4 allele* |  |  |  |  | 0.364 |  |  |  |  | 0.348 |  |  |  |  | 0.795 |
| **Total meat (**50 g per day**)** |  |  |  |  |  |  |  |  |  |  |  |  |  |  |  |
| *Stratified analysis* |  |  |  |  |  |  |  |  |  |  |  |  |  |  |  |
| *APOE* Ɛ4 non-carriers |  | 1.36 | 0.89 | 2.09 | 0.158 |  | 1.17 | 0.74 | 1.83 | 0.505 |  | 1.22 | 0.77 | 1.91 | 0.397 |
| *APOE* Ɛ4 carriers |  | 1.01 | 0.76 | 1.36 | 0.929 |  | 0.87 | 0.61 | 1.24 | 0.441 |  | 0.89 | 0.62 | 1.26 | 0.504 |
| *P for interaction with APOE Ɛ4 allele* |  |  |  |  | 0.261 |  |  |  |  | 0.236 |  |  |  |  | 0.230 |

^1^Minimally-adjusted models: Cox proportional hazards regression adjusted for age, gender, ethnicity, education, socioeconomic status. **^2^**Fully-adjusted models: Cox proportional hazards regression additionally adjusted for region, smoking status, physical activity, body mass index, sleep duration, stroke history, family history of dementia, genetic kinship to other participants, dietary covariates including vegetables and fruits, total fish, tea and coffee, alcohol drinking; processed meat, unprocessed poultry, and unprocessed red meat were also mutually adjusted for. Mean daily intakes per increment calculated from the multiple 24-h dietary assessments were used as continuous variables in Cox models. Abbreviations: HR, hazard ratio; LCI, lower confidence interval (95%); UCI, upper confidence interval (95%); *APOE,* apolipoprotein E.

## **Supplementary Table 9 Risks of all-cause dementia, Alzheimer’s disease and vascular dementia under different meat types among APOE Ɛ4 non-carriers (n=125 229) and carriers (n=49 440) respectively in participants aged 60 or more**

|  |  | **Unadjusted models**  **(n = 174 669)** | | | |  | **Minimally-adjusted**  **Models ^1^ (n = 174 669)** | | | |  | **Fully-adjusted models ^2^**  **(n = 174 669)** | | | |
| --- | --- | --- | --- | --- | --- | --- | --- | --- | --- | --- | --- | --- | --- | --- | --- |
|  |  | HR | LCI | UCI | *P* |  | HR | LCI | UCI | *P* |  | HR | LCI | UCI | *P* |
| **Risk of All-cause dementia** |  |  |  |  |  |  |  |  |  |  |  |  |  |  |  |
| *APOE Ɛ4 carriers vs. non-carriers* |  | 3.85 | 2.52 | 5.88 | <0.001 |  | 3.99 | 2.62 | 6.08 | <0.001 |  | 3.89 | 2.57 | 5.89 | <0.001 |
| **Processed meat (**25 g per day**)** |  |  |  |  |  |  |  |  |  |  |  |  |  |  |  |
| *Stratified analysis* |  |  |  |  |  |  |  |  |  |  |  |  |  |  |  |
| *APOE* Ɛ4 non-carriers |  | 1.60 | 1.24 | 2.07 | <0.001 |  | 1.30 | 1.01 | 1.69 | 0.045 |  | 1.44 | 1.10 | 1.88 | 0.009 |
| *APOE* Ɛ4 carriers |  | 1.21 | 0.96 | 1.52 | 0.110 |  | 1.11 | 0.87 | 1.40 | 0.409 |  | 1.53 | 1.19 | 1.96 | 0.001 |
| *P for interaction with APOE Ɛ4 allele* |  |  |  |  | 0.107 |  |  |  |  | 0.098 |  |  |  |  | 0.471 |
| **Unprocessed poultry (**25 g per day**)** |  |  |  |  |  |  |  |  |  |  |  |  |  |  |  |
| *Stratified analysis* |  |  |  |  |  |  |  |  |  |  |  |  |  |  |  |
| *APOE* Ɛ4 non-carriers |  | 0.85 | 0.72 | 1.01 | 0.068 |  | 0.89 | 0.75 | 1.06 | 0.180 |  | 0.89 | 0.74 | 1.07 | 0.207 |
| *APOE* Ɛ4 carriers |  | 0.82 | 0.70 | 0.96 | 0.011 |  | 0.84 | 0.72 | 0.98 | 0.026 |  | 0.89 | 0.76 | 1.04 | 0.144 |
| *P for interaction with APOE Ɛ4 allele* |  |  |  |  | 0.747 |  |  |  |  | 0.669 |  |  |  |  | 0.726 |
| **Unprocessed red meat (**50 g per day**)** |  |  |  |  |  |  |  |  |  |  |  |  |  |  |  |
| *Stratified analysis* |  |  |  |  |  |  |  |  |  |  |  |  |  |  |  |
| *APOE* Ɛ4 non-carriers |  | 1.02 | 0.78 | 1.35 | 0.884 |  | 0.93 | 0.71 | 1.22 | 0.586 |  | 0.95 | 0.70 | 1.28 | 0.724 |
| *APOE* Ɛ4 carriers |  | 0.66 | 0.52 | 0.84 | 0.001 |  | 0.62 | 0.49 | 0.79 | <0.001 |  | 0.62 | 0.47 | 0.81 | <0.001 |
| *P for interaction with APOE Ɛ4 allele* |  |  |  |  | 0.021 |  |  |  |  | 0.016 |  |  |  |  | 0.044 |
| **Total meat (**50 g per day**)** |  |  |  |  |  |  |  |  |  |  |  |  |  |  |  |
| *Stratified analysis* |  |  |  |  |  |  |  |  |  |  |  |  |  |  |  |
| *APOE* Ɛ4 non-carriers |  | 1.18 | 0.99 | 1.40 | 0.068 |  | 1.09 | 0.92 | 1.29 | 0.339 |  | 1.14 | 0.96 | 1.36 | 0.123 |
| *APOE* Ɛ4 carriers |  | 1.00 | 0.87 | 1.16 | 0.962 |  | 0.95 | 0.82 | 1.10 | 0.506 |  | 1.02 | 0.88 | 1.18 | 0.835 |
| *P for interaction with APOE Ɛ4 allele* |  |  |  |  | 0.176 |  |  |  |  | 0.126 |  |  |  |  | 0.109 |
| **Risk of Alzheimer’s disease** |  |  |  |  |  |  |  |  |  |  |  |  |  |  |  |
| *APOE Ɛ4 carriers vs. non-carriers* |  | 6.32 | 3.05 | 13.1 | <0.001 |  | 6.59 | 3.16 | 13.7 | <0.001 |  | 6.14 | 3.00 | 12.6 | <0.001 |
| **Processed meat (**25 g per day**)** |  |  |  |  |  |  |  |  |  |  |  |  |  |  |  |
| *Stratified analysis* |  |  |  |  |  |  |  |  |  |  |  |  |  |  |  |
| *APOE* Ɛ4 non-carriers |  | 1.54 | 0.94 | 2.51 | 0.086 |  | 1.36 | 0.82 | 2.24 | 0.231 |  | 1.66 | 0.97 | 2.84 | 0.063 |
| *APOE* Ɛ4 carriers |  | 1.03 | 0.73 | 1.45 | 0.873 |  | 1.01 | 0.71 | 1.44 | 0.969 |  | 1.52 | 1.05 | 2.20 | 0.027 |
| *P for interaction with APOE Ɛ4 allele* |  |  |  |  | 0.190 |  |  |  |  | 0.176 |  |  |  |  | 0.422 |
| **Unprocessed poultry (**25 g per day**)** |  |  |  |  |  |  |  |  |  |  |  |  |  |  |  |
| *Stratified analysis* |  |  |  |  |  |  |  |  |  |  |  |  |  |  |  |
| *APOE* Ɛ4 non-carriers |  | 0.89 | 0.66 | 1.22 | 0.469 |  | 0.94 | 0.69 | 1.27 | 0.687 |  | 0.91 | 0.67 | 1.24 | 0.567 |
| *APOE* Ɛ4 carriers |  | 0.86 | 0.68 | 1.09 | 0.217 |  | 0.87 | 0.69 | 1.10 | 0.254 |  | 0.93 | 0.74 | 1.18 | 0.569 |
| *P for interaction with APOE Ɛ4 allele* |  |  |  |  | 0.872 |  |  |  |  | 0.827 |  |  |  |  | 0.720 |
| **Unprocessed red meat (**50 g per day**)** |  |  |  |  |  |  |  |  |  |  |  |  |  |  |  |
| *Stratified analysis* |  |  |  |  |  |  |  |  |  |  |  |  |  |  |  |
| *APOE* Ɛ4 non-carriers |  | 0.96 | 0.58 | 1.59 | 0.880 |  | 0.92 | 0.56 | 1.51 | 0.747 |  | 0.92 | 0.53 | 1.60 | 0.760 |
| *APOE* Ɛ4 carriers |  | 0.61 | 0.44 | 0.87 | 0.005 |  | 0.59 | 0.41 | 0.84 | 0.003 |  | 0.60 | 0.41 | 0.88 | 0.009 |
| *P for interaction with APOE Ɛ4 allele* |  |  |  |  | 0.152 |  |  |  |  | 0.137 |  |  |  |  | 0.257 |
| **Total meat (**50 g per day**)** |  |  |  |  |  |  |  |  |  |  |  |  |  |  |  |
| *Stratified analysis* |  |  |  |  |  |  |  |  |  |  |  |  |  |  |  |
| *APOE* Ɛ4 non-carriers |  | 1.15 | 0.84 | 1.59 | 0.386 |  | 1.11 | 0.81 | 1.52 | 0.522 |  | 1.21 | 0.88 | 1.65 | 0.240 |
| *APOE* Ɛ4 carriers |  | 1.00 | 0.81 | 1.25 | 0.976 |  | 0.97 | 0.78 | 1.22 | 0.818 |  | 1.08 | 0.86 | 1.35 | 0.528 |
| *P for interaction with APOE Ɛ4 allele* |  |  |  |  | 0.491 |  |  |  |  | 0.431 |  |  |  |  | 0.423 |
| **Risk of Vascular dementia** |  |  |  |  |  |  |  |  |  |  |  |  |  |  |  |
| *APOE Ɛ4 carriers vs. non-carriers* |  | 6.26 | 2.17 | 18.1 | <0.001 |  | 6.56 | 2.35 | 18.4 | <0.001 |  | 6.49 | 2.37 | 17.8 | <0.001 |
| **Processed meat (**25 g per day**)** |  |  |  |  |  |  |  |  |  |  |  |  |  |  |  |
| *Stratified analysis* |  |  |  |  |  |  |  |  |  |  |  |  |  |  |  |
| *APOE* Ɛ4 non-carriers |  | 2.02 | 1.06 | 3.84 | 0.033 |  | 1.49 | 0.79 | 2.82 | 0.216 |  | 1.55 | 0.79 | 3.06 | 0.201 |
| *APOE* Ɛ4 carriers |  | 1.05 | 0.62 | 1.76 | 0.861 |  | 0.85 | 0.50 | 1.43 | 0.529 |  | 1.08 | 0.60 | 1.93 | 0.799 |
| *P for interaction with APOE Ɛ4 allele* |  |  |  |  | 0.122 |  |  |  |  | 0.110 |  |  |  |  | 0.263 |
| **Unprocessed poultry (**25 g per day**)** |  |  |  |  |  |  |  |  |  |  |  |  |  |  |  |
| *Stratified analysis* |  |  |  |  |  |  |  |  |  |  |  |  |  |  |  |
| *APOE* Ɛ4 non-carriers |  | 1.02 | 0.62 | 1.66 | 0.940 |  | 1.07 | 0.66 | 1.74 | 0.780 |  | 1.05 | 0.64 | 1.72 | 0.855 |
| *APOE* Ɛ4 carriers |  | 0.98 | 0.69 | 1.38 | 0.886 |  | 1.04 | 0.73 | 1.47 | 0.831 |  | 1.08 | 0.75 | 1.58 | 0.673 |
| *P for interaction with APOE Ɛ4 allele* |  |  |  |  | 0.884 |  |  |  |  | 0.831 |  |  |  |  | 0.736 |
| **Unprocessed red meat (**50 g per day**)** |  |  |  |  |  |  |  |  |  |  |  |  |  |  |  |
| *Stratified analysis* |  |  |  |  |  |  |  |  |  |  |  |  |  |  |  |
| *APOE* Ɛ4 non-carriers |  | 1.16 | 0.57 | 2.36 | 0.687 |  | 1.03 | 0.53 | 2.01 | 0.924 |  | 0.94 | 0.44 | 2.01 | 0.881 |
| *APOE* Ɛ4 carriers |  | 0.63 | 0.37 | 1.08 | 0.094 |  | 0.57 | 0.33 | 0.98 | 0.042 |  | 0.59 | 0.32 | 1.08 | 0.088 |
| *P for interaction with APOE Ɛ4 allele* |  |  |  |  | 0.182 |  |  |  |  | 0.152 |  |  |  |  | 0.327 |
| **Total meat (**50 g per day**)** |  |  |  |  |  |  |  |  |  |  |  |  |  |  |  |
| *Stratified analysis* |  |  |  |  |  |  |  |  |  |  |  |  |  |  |  |
| *APOE* Ɛ4 non-carriers |  | 1.36 | 0.84 | 2.20 | 0.214 |  | 1.21 | 0.78 | 1.88 | 0.406 |  | 1.24 | 0.79 | 1.95 | 0.342 |
| *APOE* Ɛ4 carriers |  | 0.95 | 0.71 | 1.27 | 0.724 |  | 0.87 | 0.65 | 1.16 | 0.345 |  | 0.89 | 0.67 | 1.19 | 0.448 |
| *P for interaction with APOE Ɛ4 allele* |  |  |  |  | 0.212 |  |  |  |  | 0.168 |  |  |  |  | 0.148 |

^1^Minimally-adjusted models: Cox proportional hazards regression adjusted for age, gender, ethnicity, education, socioeconomic status. **^2^**Fully-adjusted models: Cox proportional hazards regression additionally adjusted for region, smoking status, physical activity, body mass index, sleep duration, stroke history, family history of dementia, genetic kinship to other participants, dietary covariates including vegetables and fruits, total fish, tea and coffee, alcohol drinking; processed meat, unprocessed poultry, and unprocessed red meat were also mutually adjusted for. Mean daily intakes per increment calculated from the multiple 24-h dietary assessments were used as continuous variables in Cox models. Abbreviations: HR, hazard ratio; LCI, lower confidence interval (95%); UCI, upper confidence interval (95%); *APOE,* apolipoprotein E.

# Supplementary Methods

# 1. Assessment of dietary meat consumption

# 1.1 The baseline touchscreen questionnaire

The baseline touchscreen questionnaire contained 29 food items and 18 alcohol and beverage items. There were 5 questions on meat (fish not included) including processed meat (such as bacon, ham, sausages, meat pies, kebabs, burgers, chicken nuggets), poultry (processed poultry not counted), beef (processed beef not counted), lamb/mutton (processed lamb/mutton not counted), and pork (processed pork not counted). Participants were asked how often each item was consumed with eight options to select being: ‘never’, ‘less than once a week’, ‘once a week’, ‘2-4 times a week’, ‘5-6 times a week’, ‘once or more daily’, ‘do not know’, ‘prefer not to answer’. We converted the responses on meat into weekly-based consumption frequencies as follows: 0, 0.5, 1, 3, 5.5, and 7 times per week, where responses like ‘do not know’, ‘prefer not to answer’ were converted into missing values. Then we summed unprocessed beef, lamb/mutton, and pork into one group titled ‘unprocessed red meat’, and then we combined processed meat, unprocessed poultry, and unprocessed red meat into one group titled ‘total meat’. To rank participants by weekly meat consumption according to the distribution of data, we categorized intake frequencies for each meat type into five groups: processed meat (0, 0.1-0.9, once, 2.0-4.9 and ≥5.0 times per week); unprocessed poultry (0, 0.1-0.9, once, 2.0-4.9 and ≥5.0 times per week); unprocessed red meat (0, 0.1-1.0, 1.1-1.9, 2.0-2.9 and ≥3.0 times per week); and total meat (0, 0.1-3.0, 3.1-4.9, 5.0-6.9 and ≥7.0 times per week). These categories were determined for similar-sized groups where data distribution allowed.

# 1.2 The Oxford WebQ questionnaire for 24-h dietary assessments

The Oxford WebQ questionnaire containing up to 206 food items and 32 alcohol and beverage items assessed dietary consumption information over the previous 24 hours. Participants were asked to select how many portions they consumed for each item with instructions specifying what one portion size is, such as one sausage, one rasher of bacon, one slice of ham, or one ‘serving’ for some specific foods. The food intake weight in grams for each item was calculated by multiplying amounts of portion size by standard portion sizes in grams [1]; then daily intakes of energy and nutrients were estimated by multiplying the food weight consumed by its nutrient composition based on McCance and Widdowson’s the Composition of Foods (Seventh Summary Edition) [2]. There were 10 items on meat (fish not included) asking the amount of portion size consumed for previous 24 hours with 7 options to select being ‘none’, ‘half’, ‘1’, ‘2’, ‘3’, ‘4’, ‘5+’, where the top open-ended category was coded as 5 in analyses. To match meat groups from the baseline touchscreen questionnaire as closely as possible, meat-related items from the Oxford WebQ 24-h dietary assessments were grouped as follows: processed meat (sum of sausage, bacon, ham, crumbed chicken or turkey), unprocessed poultry (chicken or turkey), unprocessed red meat (sum of beef, lamb/mutton, pork), and total meat (sum of processed meat, unprocessed poultry, and unprocessed red meat).

The Oxford WebQ questionnaire was introduced into the baseline recruitment visit as an enhancement to the baseline measures for the last 70724 participants. After recruitment, participants who agreed to be contacted by e-mails were invited to complete an online Oxford WebQ questionnaire for 24-hour dietary assessments. The Oxford WebQ questionnaire was administrated online once every 3–4 months for a total of four separate occasions between February 2011 and June 2012. A large sub-sample of participants (n = 175402) completed at least one online WebQ questionnaire. Taking respondents to the WebQ questionnaire at the baseline assessment visit into account, there were in total 211006 participants who completed at least one (1+) WebQ questionnaire, 126844 participants who completed at least two (2+) WebQ questionnaire, 78725 participants who completed at least three (3+) WebQ questionnaire. Meat intakes in grams were averaged for participants who completed more than one Oxford WebQ questionnaire. Comparisons of baseline characteristics and meat consumption from 24-h dietary assessment across participants with 1+, 2+ and 3+ completions of WebQ questionnaire were summarized in Supplementary table 1, showing that participants with more numbers of completion to the WebQ questionnaire were more likely to be women, older, of white ethnic background, less deprived, and more educated; however, the mean daily intakes of the meat groups were similar across participants with 1+, 2+ and 3+ completions with narrower 95% confidence intervals as number of completion increases. Although the more numbers of completion the more stable the mean consumption is, the restriction to 3+ completions of the WebQ questionnaire increased the risk of selection bias; so, we included participants who completed at least two Oxford WebQ questionnaires as indicated in previous study [3].

Within each category of meat groups (processed meat, unprocessed poultry, unprocessed red meat, and total meat) defined from the baseline touchscreen questionnaire, we calculated the mean intakes in grams of the same food group from participants who completed the Oxford WebQ questionnaires; then we assigned the mean intakes from the 24-h dietary assessment correspondingly to each category of the meat groups to indicate a level of meat consumption from low to high categories. Since there might be a disproportionate number of participants who reported meat consumption lower or higher than their ‘true intake’ in the lowest category and highest category respectively, regression dilution bias might have occurred in the baseline touchscreen dietary assessment [4,5]. To correct for the potential regression dilution bias, we generated a trend variable based on mean intakes in grams for each category of meat groups using the following increments: processed meat (25 g/day); unprocessed poultry (25 g/day); unprocessed red meat (50 g/day); and total meat (50 g/day). These increments were chosen based on average increases across category differences of each meat type consumed in this cohort and were used for estimation of risk of disease per increment (in g/d) in meat consumption.

# 2. Determination of the minimal adjustment set

We used the Directed acyclic graph (DAG) via the online tool DAGitty (<http://www.dagitty.net/>) to determine the minimal adjustment set. The DAG below shows the relationships among the exposure (Meat intake; represented by the green oval with the triangle), the outcome (Dementia; represented by the blue oval with the line), and related factors.


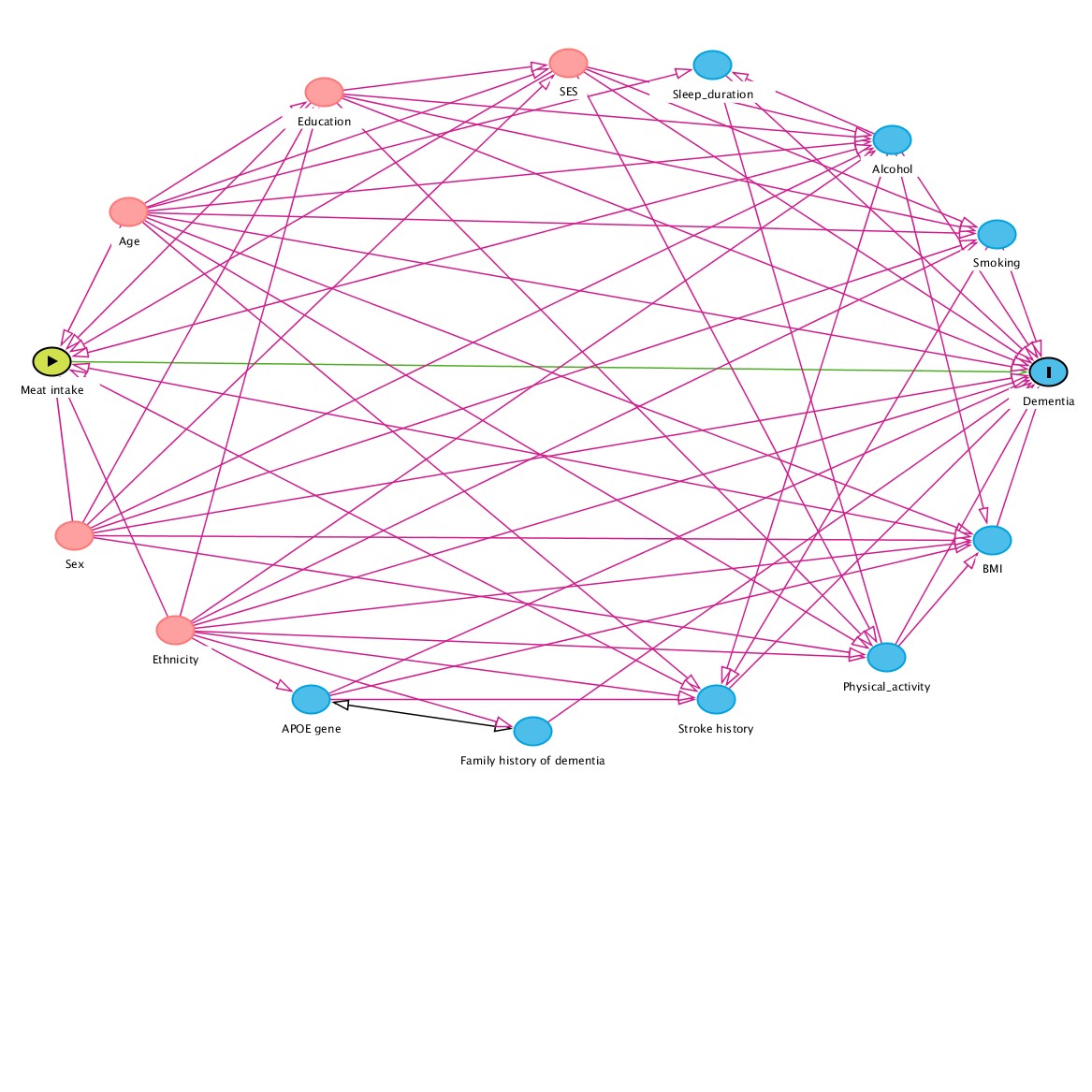


Variables represented as pink ovals are ancestors of exposure and outcome while variables represented as blue ovals are ancestors only of the outcome. Pink lines are biasing paths and the green line between the exposure and outcome is the causal path of interest. SES, social economic status; BMI, body mass index.

# 3. Covariates

# 3.1 Dietary variables

The baseline touchscreen questionnaire was designed to collect basic dietary information on some commonly consumed foods or food groups; thus, it was recognised that this questionnaire was not suitable to assess total energy or nutrients intakes. In general, we grouped participants into four categories for each food group according to the distribution of data to get approximately equal-sized categories. A ‘unknown’ category was additionally created to replace missing values for each covariate. Three food groups (total fish, fruits and vegetables, tea and coffee) that potentially confounds associations between meat consumption and risk of dementia were adjusted for in fully-adjusted models as categorical variables.

Fish

There were 2 questions on fish including oily fish (such as sardines, salmon, mackerel, herring), and other types of fish (such as cod, tinned tuna, haddock) with eight options to select. The same approach used for meat-related items was used to deal with fish items. We summed oily fish and other types of fish into ‘total fish’, and then grouped weekly-based consumption frequencies of total fish into four categories as follows: ≤1.0, 1-2, ≥2, and ≥3 times per week.

Fruit and vegetables

Participants were asked to either direct input the specific daily numbers of consumed pieces of fresh fruit (one apple, one banana, 10 grapes etc as one piece), pieces of dried fruit (one prune, one dried apricot, 10 raisins etc as one piece), heaped tablespoons of cooked vegetables and heaped tablespoons of salad/raw vegetables, or select ‘less than one’, ‘do not know’ or ‘prefer not to answer’ over four separate questions. One piece of fresh fruit, two ‘pieces’ of dried fruit, two heaped tablespoons of cooked vegetables, and two heaped tablespoons of salad/raw vegetables were counted as one serving respectively. We summed these four items into one ‘fruits and vegetables’, and then we grouped the daily servings of fruits and vegetables into four categories as follows: <2, <4, 4-6, and >6 servings per day.

Tea and coffee

Participants were asked to either direct input the specific numbers of cups of tea (including black and green tea), cups of coffee (including decaffeinated coffee) drunk per day, or select ‘less than one’, ‘do not know’ or ‘prefer not to answer’ over two separate questions. We summed cups of tea and coffee together, and grouped participants into four categories as follows: ≤3, ≤5, ≤7, and >7 cups per day.

Alcohol drinking

Participants were asked how often they drink alcohol with 7 options: daily or almost daily, 3-4 times a week, once or twice a week, 1-3 times a month, special occasions only, never, or prefer not to answer. We grouped these options into four categories as follows: less than once a week, once or twice a week, three or four times a week, and daily or almost daily.

# 3.2 Socio-demographics

Age at baseline

Age at baseline was calculated as year differences between birth dates and dates of assessment center visits and was treated as a continuous variable in adjustment sets.

Ethnicity

Participants were asked to select their ethnic group among ‘White’ (including British, Irish, any other White background), ‘Mixed’ (including White and Black Caribbean, White and Black African, White and Asian, any other mixed background), ‘Asian or Asian British’ (including Indian, Pakistani, Bangladeshi, any other Asian background), ‘Black or Black British’ (including Caribbean, African, any other Black background), ‘Chinese’, ‘Other ethnic group’, ‘Do not know’ or ‘Prefer not to answer’. We re-grouped ethnicity into 5 categories as follows: White (White, British, Irish or any other white background); Asian or Asian British (Asian or Asian British, Chinese, Indian, Pakistani, Bangladeshi or any other Asian background); Black or Black British (Black or Black British, Caribbean, African or any other Black background); Mixed Race or Other (any other ethnic groups or mixed ethnicity); and unknown (included participants who did not know or preferred not to answer)

Region

Participants were recruited via 22 assessment centers across UK. We grouped the centers into three regions as follows: England (St Bartholomew’s Hospital, Hounslow, Croydon, Stockport, Manchester, Liverpool, Bury, Newcastle, Middlesbrough, Leeds, Sheffield, Stoke, Birmingham, Nottingham, Oxford, Reading, Bristol), Wales (Swansea, Wrexham, Cardiff), Scotland (Glasgow, Edinburgh).

Townsend deprivation Index (TDI)

The Townsend deprivation index was calculated to reflect the socio-economic level of participants based on postcode-specific information on percentage of unemployment, percentage of overcrowded households, percentage of people with no car ownership, and percentage of non-home owners [6]. The higher the score was the more deprived the participants were. We then categorized the score into three equal-sized groups as follows: low deprivation, moderate deprivation, and high deprivation. Given the risk of over-adjustment, we only included TDI as a categorical variable in adjustment sets without extra adjusting for employment and home incomes.

Education

Participants were asked to select their acquired qualifications among ‘College or University degree’, ‘A levels/AS levels or equivalent’, ‘O levels/GCSEs or equivalent’, ‘CSEs or equivalent’, ‘NVQ or HND or HNC or equivalent’, ‘Other professional qualifications e.g.: nursing, teaching’, ‘None of the above’ and ‘Prefer not to answer’. We regrouped these qualifications into ‘with college/university degree’ and ‘without college/university degree’ based on higher education criteria in UK (<https://www.nidirect.gov.uk/articles/what-higher-education>).

# 3.3 Lifestyle related and other covariates

Body Mass Index (BMI)

Standing height and weight were measured at baseline according to standard protocol. BMI was calculated using the formular ‘BMI (kg/m^2^) = Weight (kg) / Height^2 (m^2^)’. We then categorized the BMI into three groups based on the data distribution as follows: normal or underweight <25 kg/m^2^, overweigh 25-29.9 kg/m^2^, and obese ≥30 kg/m^2^ according to the World Health Organisation (WHO) and the National Institute for Health and Clinical Excellence (NICE) [7].

Physical activity

Participants were asked a serial of questions about their usual daily activities at baseline that were taken from the International Physical activity questionnaire (IPAQ) short form [8]. Physical activity was calculated and categorized into three levels being low, moderate, and high, according to the official guidelines for data processing and analysis of the IPAQ Short Forms (<https://biobank.ctsu.ox.ac.uk/crystal/crystal/docs/ipaq_analysis.pdf>).

Smoking status

Participants were asked about specific smoking behaviours in current days and previous days separately. We regrouped these behaviours into ‘Current’ (meaning smoking in current days no matter what situations of previous days were), ‘Past’ (meaning smoking or alcohol drinking in previous days only but not in current days), and ‘Never’ (mean no smoking or alcohol drinking in either current days or previous days).

Sleep duration

Participants were asked about their sleep duration over a question "how many hours sleep do you get in every 24 hours? (include naps)" with rejection of <1 hour or >23 hours, and requests to confirmation of <3 hour or >12 hours. We categorized the sleep duration into <7, 7-8, >8 hours/day based on data distribution and used it as a categorical variable in adjustment sets.

Stroke history

Participants were asked to report whether they have suffered from the following illnesses including heart disease, stroke, diabetes etc. with multiple choices available. Participants who have selected stroke were considered to be having a stroke history.

Family history of dementia

Participants were asked to report whether their family members (father, adopted father, mother, adopted mother, brothers or sisters, adopted brothers or sisters) have suffered from any of listed illnesses (including heart disease, stroke, diabetes etc. with multiple choices available). Participants who have any biological family members with Alzheimer’s disease/dementia were considered to be having family history of dementia.

# Supplementary References

1. Ministry of Agriculture, Fisheries and Food (1993) Food Portion Sizes. London: HMSO.

2. Finglas PM, Roe MA, Pinchen HM, et al. (2015) McCance and Widdowson’s The Composition of Foods, Seventh Summary Edition. Cambridge: Royal Society of Chemistry.

3. Carter JL, Lewington S, Piernas C, Bradbury K, Key TJ, Jebb SA, Arnold M, Bennett D, Clarke R. Reproducibility of dietary intakes of macronutrients, specific food groups, and dietary patterns in 211 050 adults in the UK Biobank study. J Nutr Sci 2019;8:e34.

4. Bradbury KE, Young HJ, Guo W, Key TJ. Dietary assessment in UK Biobank: an evaluation of the performance of the touchscreen dietary questionnaire. J Nutr Sci. 2018;7:e6.

5. MacMahon S, Peto R, Cutler J, Collins R, Sorlie P, Neaton J, Abbott R, Godwin J, Dyer A, Stamler J. Blood pressure, stroke, and coronary heart disease. Part 1, Prolonged differences in blood pressure: prospective observational studies corrected for the regression dilution bias. Lancet 1990;335:765-74.

6. Townsend P, Phillimore P, Beattie A. Health and deprivation: inequality and the North. Routledge; 1988.

7. WHO Expert Committee on Physical Status: the Use and Interpretation of Anthropometry (‎1993: Geneva, Switzerland)‎ & World Health Organization. (‎1995)‎. Physical status: the use of and interpretation of anthropometry, report of a WHO expert committee. World Health Organization. https://apps.who.int/iris/handle/10665/37003

8. Sjöström M, Ainsworth B, Bauman A, Bull F, Craig C, Sallis J. Guidelines for data processing and analysis of the International Physical Activity Questionnaire (IPAQ)–short and long forms. IPAQ core group. 2005.
